# The quantitation of buffering action I. *A formal & general approach*

**DOI:** 10.1186/1742-4682-2-8

**Published:** 2005-03-15

**Authors:** Bernhard M Schmitt

**Affiliations:** 1 Department of Anatomy, University of Würzburg, 97070 Würzburg, Germany

## Abstract

**Background:**

Although "buffering" as a homeostatic mechanism is a universal phenomenon, the quantitation of buffering action remains controversial and problematic. Major shortcomings are: lack of a buffering strength unit for some buffering phenomena, multiple and mutually incommensurable units for others, and lack of a genuine ratio scale for buffering strength. Here, I present a concept of buffering that overcomes these shortcomings.

**Theory:**

Briefly, when, for instance, some "free" H^+ ^ions are added to a solution (e.g. in the form of strong acid), buffering is said to be present when not all H^+ ^ions remain "free" (i.e., bound to H_2_O), but some become "bound" (i.e., bound to molecules other than H_2_O). The greater the number of H^+ ^ions that become "bound" in this process, the greater the buffering action. This number can be expressed in two ways: *1*) With respect to the number of total free ions added as "buffering coefficient b", defined in differential form as b = d(bound)/d(total). This measure expresses buffering action from nil to complete by a dimensionless number between 0 and 1, analogous to probabilites. *2*) With respect to the complementary number of added ions that remain free as "buffering ratio B", defined as the differential B = d(bound)/d(free). The buffering ratio B provides an absolute ratio scale, where buffering action from nil to perfect corresponds to dimensionless numbers between 0 and infinity, and where equal differences of buffering action result in equal intervals on the scale. Formulated in purely mathematical, axiomatic form, the concept reveals striking overlap with the mathematical concept of probability. However, the concept also allows one to devise simple physical models capable of visualizing buffered systems and their behavior in an exact yet intuitive way.

**Conclusion:**

These two measures of buffering action can be generalized easily to any arbitrary quantity that partitions into two compartments or states, and are thus suited to serve as standard units for buffering action. Some exemplary treatments of classical and non-classical buffering phenomena are presented in the accompanying paper.

## Background

### Buffering: a paradigm with growing pains

Buffering is among the most important mechanisms that help to maintain homeostasis of various physiological parameters in living organisms. This article is concerned with the definition of an appropriate scientific unit, or scale, for the quantitation of buffering action – a quantity that has been termed "buffering strength", "buffering power", "buffer value", or similarly [1,2]. On the one hand, the concept of "buffering" is applied in a growing number of scientific and engineering disciplines. On the other hand, the units that are currently used to measure buffering – often created on an *ad hoc *basis – suffer from fundamental inconsistencies and shortcomings. Comparison with "mature" and standardized scientific units, e.g. those of the "Système International des Unités" ("SI"), highlights the extent of these shortcomings (*see below*). As a consequence, there are multiple "local" theoretical buffering concepts with limited power, and the practical treatment of buffering phenomena is complicated unnecessarily. Thus, rethinking the quantitation of buffering action is not an effort to reinvent the wheel; rather it seems that "the wheel" has not been invented yet. Our thesis is that buffering action can be quantitated in a better, simpler, and universal way when buffering is conceived as a purely formal, mathematical principle. In this article, we present such a formal concept of buffering. Compared to existing buffering concepts, its major achievements are formal rigor and scientific richness.

#### "Buffering" – a paradigm useful in many fields

A look at the current usage of the term "buffer" suggests that a corresponding fundamental principle is common to a great variety of disciplines. Buffering concept and terminology originated in acid-base physiology at the end of the 19^th ^century when it had become clear that several biological fluids "undergo much less change in their reaction after addition of acid or alkali than would ordinary salt solutions or pure water" [2]. Hubert and Fernbach had introduced the term "buffer"; Koppel and Spiro suggested the terms "moderation" and "moderators" instead [2].

The concept of buffering was soon adopted in an increasing number of different contexts, including buffering of other electrolytes (e.g. Ca^++ ^and Mg^++^), of non-electrolytes, of redox potential, and numerous other quantities inside and outside the realm of chemistry. Examples are presented in Additional file 1.

#### Expressing the magnitude of buffering action is problematic

The common idea behind these diverse phenomena is that "buffering" is present when a certain parameter changes less than expected in response to a given disturbance, i.e., the buffer absorbs or diverts a certain fraction of the disturbance. Very soon after the concept of "buffering" had emerged it became apparent that buffering is not just absent or present in a binary sense, but instead may be "strong" or "weak". In fact, this "buffering strength" could differ over a wide range. Moreover, chemists, physiologists, and clinicians realized the great practical importance of this quantitative aspect of buffering [3], and struggled to get a numerical grip on it with the aid of various units or scales. Researchers in other areas followed. By now, buffering strength units are available for some, but not all buffering phenomena. In some cases, e.g. the buffering of ions in aqueous solutions, there exist even multiple units that are used in parallel (Additional file 2, Table 1). One can thus certainly manage to "put numbers" on these buffering phenomena. For many other types of buffering, however, units do not exist at all. For instance, no such scales are available for "blood pressure buffering" and for "cognitive buffering". Without a buffering strength unit, however, it is obviously difficult to formulate and test quantitative hypotheses regarding buffering phenomena.

**Table 1:** Interconversions of units for H^+ ^buffering strength.

***Parameter***	***Definition***	***f(B)***	***f(β)***	***f(β***_*c*_)
**B**		(B)		β_c _- 1
β_H+_		(B+1) × 2.3 × 10^pH^	(β)	β_c _× 2.3 × [H^+^]_free_
β_c_		B + 1		(β_c_)

B: "buffering odds" according to this article, : "buffering value" according to Van Slyke [4]; : "buffering coefficient" according to Saleh et al. [6]. First row: given source units; first column: desired target units; intersection of particular row and particular column: transformation, i.e., functions of the respective source unit, that yields the target unit.

In the past, researchers have exhibited a surprisingly high degree of tolerance towards the shortcomings and ambiguities inherent to the current approaches to the quantitation of buffering action. However, these drawbacks have already caused problems and confusion, both on a theoretical and practical level, and will become even more problematic and disturbing as the buffering paradigm is applied more widely. The systematic analysis of the available concepts and scales of "buffering" presented in Additional file 2 substantiates this criticism and points to the features that would be required to make an ideal scale of buffering strength.

Briefly, this analysis of the available units of "buffering strength" reveals three major problems: *i*) Intrinsic deficiencies: Scales are second-rate inasmuch as only some of the mathematical operations can be applied to the measurements that would be applicable with different types of scales; *ii*) Limitedness, both conceptual and practical: Individual units can handle only selected special cases of buffering, whereas other types of buffering require different units or cannot be quantitated at all; *iii*) Confusion & inconsistencies: A motley multiplicity of units and definitions actually houses disparate things, thus obfuscating the simple, common principle behind the various buffering phenomena.

Accordingly, a quantitative measure of buffering would ideally provide *i*) a scale of the highest possible type, namely a "ratio scale". Ratio scales are scales with equal intervals and an absolute zero. For instance, when H^+ ^ion concentration is expressed in terms of moles per liter, this measure increases by the same amount irrespective of the initial concentration (equal intervals). In contrast, when H^+ ^ion concentration is expressed, for instance, in terms of pH, this measure of concentration will change only a little at low pH, but much at high pH (non-equal intervals). One example for a scale without an absolute zero, on the other hand, is provided by the Celsius and Fahrenheit scales for temperature where the position of 0° is arbitrary, whereas 0° on the Kelvin scale is an "absolute" zero (as would be a probability of zero, a capacitance of 0 Farad, a mass of 0 kg etc.); *ii*) a scale that is universal, allowing for adequate quantitation of buffering behavior in all its manifestations (i.e., irrespective of its particular physical dimension, and including moderation, amplification, and the complete absence of buffering); *iii*) a scale that could be used as a general standard, within a given discipline and across different disciplines. The first two properties mentioned (ratio scale and universal applicability) would automatically generate a scale that could serve as such an all-purpose yardstick of buffering strength. However, there is clearly no such scale available to date.

## A formal and general approach to the quantitation of buffering action

### An intuitive introduction of the approach

#### Buffering processes as partitioning processes

**Figure 1:**
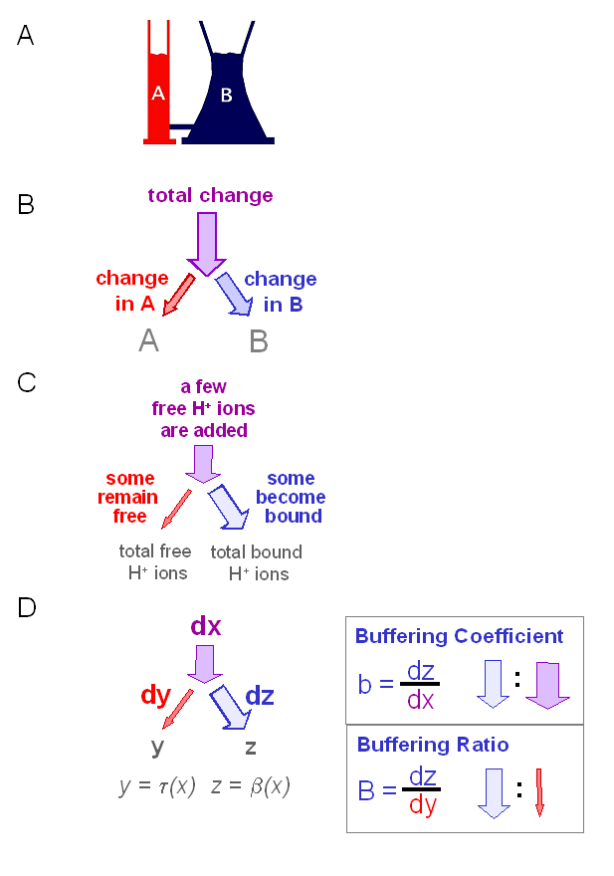
***A simple quantitative criterion of buffering action.***(See main text for detailed explanation) ***A, Communicating vessels model of partitioning processes. ***In a system of two communicating vessels (A and B), total fluid volume is the sum of the two partial volumes in A and B. In an equilibrated system, the partial volumes in the individual vessels can be described as functions of total fluid volume; these functions are termed "partitioning functions". The derivatives of the partitioning functions tell what fraction of a total volume change is conveyed to the respective vessel. **B, Partitioning of a quantity in a two-compartment system. **A given total change of quantity in the system produces two partial changes in compartments A and B. The greater the partial change in B, the smaller the change in A, and the greater the "buffering" of the quantity in A. **C, Partitioning of H^+ ^ions between water and buffer. **Free H^+ ^ions are added to an aqueous solution containing a weak acid (e.g. as strong acid). Some of the added H^+ ^ions remains free, some become bound to buffer molecules. ***C, General definition of measures of buffering action. ***The differential dz/dy, paraphrased as d(buffered)/d(total), is termed the buffering coefficient b. The differential, paraphrased as d(buffered)/d(unbuffered), is termed the buffering ratio B.

Universal measures of buffering action can be developed if one views the underlying process as a "partitioning" process. To explain what we mean by this, consider two arbitrarily shaped vessels that are filled with a fluid and connected via a small tube (Figure 1A). The fluid in such a system of communicating vessels will distribute in such a way that the two individual fluid levels become equal. By virtue of hydrostatic pressure, any given total fluid volume is thus associated with a unique partial volume in the first vessel, and with another unique partial volume in the second one.

Now, let us add a small volume of extra fluid into the system. When the system has reached the corresponding new equilibrium state, a portion of the extra fluid is found in the vessel A, another portion in vessel B. Clearly, the volume change in vessel A in response to a given volume load is smaller when this vessel is part of this system of vessels, as compared to vessel A standing alone and subjected to the same load. We can say, the system is able to stabilize or "buffer" fluid volume in vessel A in the face of increases or decreases of total volume.

This example shows that buffering can be viewed in terms of a partioning process in a system of two complementary compartments. "Fluid volumes" are readily replaced by other physical, chemical or other quantities. For instance, the classic case of H^+ ^buffering can be represented in a straightforward way as the partitioning of H^+ ^ions into the pool of "free" H^+ ^ions (i.e., H^+ ^ions bound to water, corresponding to vessel A) and the complementary pool of "bound" H^+ ^ions (i.e., H^+ ^ions bound to buffer molecules, corresponding to vessel B).

##### A simple criterion of buffering strength

We now formulate a simple quantitative criterion of buffering action, first in terms of fluid volumes: The more of a given fluid volume added to the system of communicating vessels ends up in vessel B, the greater the stabilization or "buffering" of the fluid volume in vessel A. Or in acid-base terms: The more of a given amount of H^+ ^ions (added, for instance, in the form of strong acid) becomes bound by buffer molecules, the more the concentration of "free" H^+ ^ions is stabilized or buffered. We can easily formulate that criterion in a general form, free of reference to any particular quantity: The greater the change of a given quantity in one individual compartment, the greater the buffering of that quantity in the other compartment (Figure 1B). Herein, the magnitude of the change in a compartment may be expressed either relative to the total change, or relative to the complementary change in the other compartment. The example of communication vessels also shows that the magnitude of change (when expressed in either of these ways) is not affected by the direction of the change: it remains the same whether the quantity in question is added to the system, or whether it is subtracted.

Unspectacular and intuitive as it may appear, this criterion will lead to conclusions that differ considerably from established views. For instance, it is usually held (on the basis of Van Slyke's definition of buffering strength [4]) that a weak acid buffers H^+ ^ions most strongly when H^+ ^ion concentration is equal to the acid constant K_A _(i.e., when [H^+^] = K_A_). However, this is not where the fraction of added H^+ ^ions binding to buffers is greatest. Rather, this fraction reaches a maximum when [H^+^] approaches zero (Figure 1C). According to our simple criterion, that is the point of maximum buffering strength (i.e., when [H^+^] = 0). Similarly, when H^+ ^ions are removed from such a solution (e.g. by addition of strong base), the fraction supplied via deprotonation of buffer molecules (as opposed to a decrease of free [H^+^]) is greatest at low total [H^+^]. This classic case illustrates the impact of the various buffering strength units on our perception of buffering strength, and is analyzed in detail, together with several further examples, in the accompanying paper (*Buffering II*^10^). Our concept of buffering results, ultimately, from the systematic application of this simple criterion.

##### Deriving quantitative measures of buffering strength from this criterion

With our simple criterion at hand, all that is left to do in order to quantitate buffering action is to put numbers on the magnitude of the change in the compartment that buffers or stabilizes the other compartment (termed "buffering compartment", corresponding to vessel B in Figure 1A). This can be done in two equally useful ways (Figure 1D):

Firstly, change in the "buffering compartment" can be expressed with respect to the total change in the system. The resulting measure represents a "fractional change", here termed "buffering coefficient b"



The buffering coefficient b thus indicates the proportion between one particular part and the whole.

Secondly, change in the "buffering compartment" can be expressed with respect to the complementary change in the other compartment, termed "target compartment" or "transfer compartment", to indicate that one views this compartment as the one for which the imposed change is "intended" (corresponding to vessel A in Figure 1A). We thus obtain a second measure, here termed the "buffering ratio B":



The buffering ratio B thus indicates the proportion between the two parts of a whole. This measure is completely analogous to the "odds" as used for the quantitation of chance (mainly by epidemiologists) and may therefore be termed synonymously "buffering odds B".

**Figure 2:**
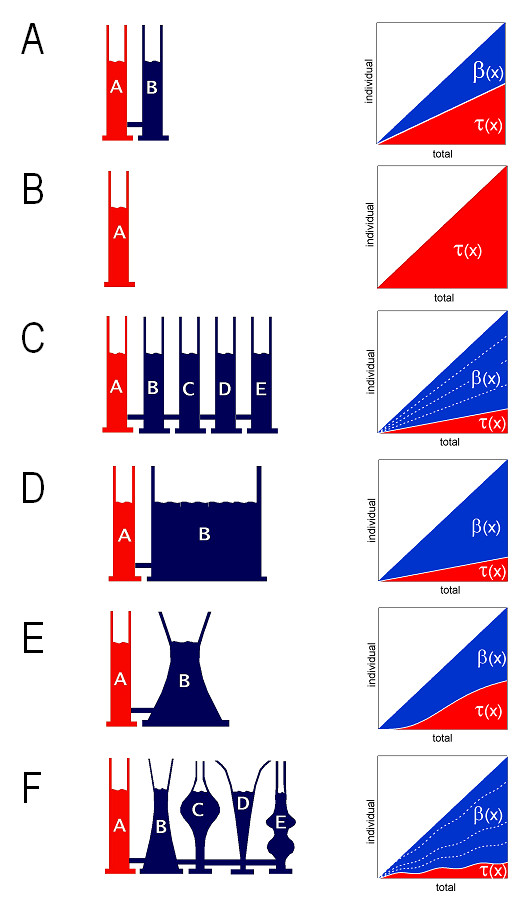
***Communicating vessels as a physical model for a buffered system.*** Total fluid volume is taken as x, fluid volume inside vessel A ("transfer vessel", *red*) as the value of the transfer function τ(x), and aggregate fluid volumes in the other vessels ("buffering vessels", *blue*) represent the "buffering function" β(x). We can describe these systems in terms of our two measures of buffering action, namely the buffering coefficient b(x) = β'(x)/[τ'(x) + β'(x)] and the buffering ratio B(x) = β'(x)/τ'(x) (*see main text for detailed explanation*). ***A, Linear buffering, one buffering vessel. ***The volume changes in A are only half as big as the total volume changes in the system; the volume inside A is "buffered", or, more specifically, "moderated". The degree of moderation is the same at all fluid levels; b(x) = constant = 0.5 and B(x) = constant = 1. ***B, Zero buffering, or perfect transfer. ***Changing total volume in the system translates completely into identical volume changes in vessel A, without "moderation" or "amplification": b(x) = 0 B(x) = 0. ***C, Linear buffering, several buffering vessels. ***Increasing the number of buffering vessels increases buffering action. The four partitioning functions are replaced by a single buffering function β. Buffering parameters are b(x) = 0.8 and B(x) = 4. ***D, Linear buffering, general case. ***Same buffering behavior as in *C*, brought about by a single buffering vessel. ***E, Non-linear buffering, one buffering vessel. ***In this system, the individual volume changes are not linear functions of total volume. Consequently, the proportion between volume flow into or out of vessels A is not a constant, but a variable function of the system's filling state. ***F, Non-linear buffering, several buffering vessels. ***In most buffered systems, buffering is brought about by a multiplicity of buffers (*as in C*) that are non-linear in their individual ways (*as in E*). Buffering coefficient and buffering odds provide overall measures of buffering action that neither require nor deliver any knowledge about the individual components.

In the following section, we illustrate a few characteristic types of buffering, using again fluid-filled communicating vessels as an example (Figure 2).

#### Use of buffering coefficient and buffering ratio for the quantitation of buffering action – some typical examples

##### A simple buffered system

Consider a system of two communicating vessels, both having identical dimensions and constant cross sectional areas (Figure 2A, *left panel*). We consider vessel A our compartment of interest (i.e., the "target" or "transfer compartment"), and ask how much the fluid volume inside it is stabilized or "buffered". To determine the degree of buffering, we titrate the system up and down by adding or removing fluid. We find that the volume changes in A are always only half as big as the changes of total volume in the system; the volume inside A is "buffered".

The behavior of the system is repesented graphically on the right hand of Figure 2A. Total volume is plotted on the abscissa. The individual volumes in vessels A and B at a given total volume are indicated in this "area plot" by the respective heights of the two superimposed areas at that point. Volumes inside vessel A and B are thus expressed as functions of the independent variable "total volume". We denote that variable by the letter x. Moreover, the volume in the transfer vessel A expressed as a function of total volume is termed the "target function" or "transfer function", denoted τ(x), and the volume in the buffering vessel B expressed as a function of total volume is termed the "buffering function", denoted β(x). "Change" in a compartment then can be defined more specifically as the first derivative of the particular function with respect to the independent variable, notated briefly as τ'(x) or β'(x).

The buffering coefficient b, defined above as the ratio of "*volume change in vessel B" *over "*total volume change in the system*", can then be expressed more simply and generally as

b = β'(x)/[τ'(x) + β'(x)].

In this system, total change equals the sum of the individual changes (other systems are covered below), and thus

τ'(x) + β'(x) = 1,

and hence

β'(x)/[τ'(x) + β'(x)] = β'(x)/1 = β'(x).

Because the buffering function β(x) equals 0.5·x in this system, we obtain a dimensionless buffering coefficient of b = 0.5. In words, a buffering coefficient of 0.5 says that of the total change imparted to the system, a fraction of 0.5 (or 50%) is directed to the "buffering compartment".

The buffering ratio B, on the other hand, which was defined above as the ratio of "*volume change in vessel B*" over "*volume change in vessel A*", can then be expressed as

B = β'(x)/τ'(x).

With τ(x) = β(x) = 0.5·x in this system, we find a value of B = 1. In words, a buffering ratio of 1 says that when a certain change is imposed to the system, the change in the target compartment is always associated with a similar sized change in the buffering compartment. In terms of fluid volume: for every drop going into or out of vessel A, another drop goes into or out of vessel B.

##### An unbuffered system

Figure 2B shows a system without a "buffering vessel". Accordingly, changes in total volume are completely translated into exactly equal changes of volume in vessel A. Again, the point here is how to express this type of buffering behavior numerically. Change in the transfer vessel A is given by a transfer function τ(x) = x, and change in the buffering vessel, given its non-existence or zero volume, by a buffering function that has a constant value of zero: β(x) = 0. We compute the buffering coefficient b again as b = β'(x) and find that b = 0, and compute the buffering ratio B as B=β'(x)/τ'(x) and find that B = 0. We see that both measures yield scales with an "absolute zero", i.e., where the position of "zero" does not depend on some arbitrary external reference (as would be the case with electrical or thermodynamical potentials, for instance) or on some similarly arbitrary convention (such as for the Celsius scale for temperature), but follows inescapably from the definition of the unit.

Again, it may appear trivial to find zero values for buffering strength in the absence of buffering. However, this desirable property of a buffering strength unit is not the rule, including the widely used H^+ ^buffering strength unit introduced by Van Slyke. This unit, defined as β = d(Strong Base)/dpH, will always be greater than zero even in the complete absence of buffering; even stranger, the particular numerical value representing the absence of buffering will vary with pH (see detailed discussion in *Buffering II*^10^).

##### Multiple buffering vessels vs. an equivalent single one

Next, as shown in Figure 2C, we add several additional copies of similar buffering vessels (vessels B,C,D,E). Compared to a single buffering vessel B, this alteration results, of course, in increased buffering action. When one compares the initial situation with a single buffering vessel to the system comprising four such vessels, it is reasonable to say that buffering action increases four-fold. However, we are not yet in a position to compute the buffering coefficient of buffering ratio.

In principle, the volumes in these vessels can be expressed by several individual functions which may be termed "partitioning functions". However, what matters with respect to the stabilization or buffering of the volume in vessel A is only their aggregate volume as a function of total volume. This aggregate function, i.e., the four partitioning functions lumped together into a single function, represents our "buffering function β(x)". With respect to buffering, the system in Figure 2C is thus perfectly equivalent to the system in Figure 2D. In both systems, the buffering function has the value of β(x) = 0.8·x, and we thus find a buffering coefficient of b = 0.8, and a buffering ratio of B = 4.

Indeed, the buffering ratio increases accordingly from B = 1 to B = 4. This behavior is typical for a "ratio scale", and is a desired property. Ratio scales not only represent the phenomena under study in a particularly intuitive way, they are also the highest type of scale inasmuch they allow meaningful application of the widest range of mathematical operations, including averaging, expression as percentage, and comparison in terms of ratios.

In contrast, the buffering coefficient changed from 0.5 to 0.8. Evidently, the buffering coefficient does not yield a ratio scale: the four-fold increase in the number of buffering vessels is reflected in an only 1.6-fold increase of the buffering coefficient. Another four-fold increase from 4 to 16 buffering vessels would entail an even smaller increase of the buffering coefficient, from 0.8 to 0.94, an approximately 1.2-fold increase.

##### Systems exhibiting non-constant buffering

In the system depicted in Figure 2E, the cross-sectional area of the buffering vessel is not constant, but varies with fluid level. As a consequence, the individual volumes in vessels A and B changes are not linear functions of total volume of the type y = constant·x, but may be any arbitrary non-linear function. The proportion between the two individual changes in vessels A and B is therefore not constant, but varies depending on the system's filling state. The two measures of buffering action can be computed exactly as indicated above as β'(x) and β'(x)/τ'(x), respectively, but the results are valid only for the given value of x. Consequently, buffering coefficient and buffering ratio must be presented as b(x) and B(x), respectively, where x specifies the filling state of the system. Such variable buffering is found in most buffered systems of scientific interest, including buffering of H^+ ^and Ca^++ ^ions in plasma and cytosol.

##### Non-constant buffering with multiple irregular buffering vessels

Figure 2F carries this more realistic version one step further, inasmuch as buffering is also often brought about by several different buffers each of which may be non-linear in its own way. This situation is replicated by a combination of several, irregularly shaped buffering vessels. A buffering function β(x) is again obtained by lumping together the individual partitioning functions of the buffering vessels into a single aggregate buffering function. Buffering coefficient and buffering ratio are then computed in the known way for a given value of x. Buffering coefficient and buffering ratio provide overall measures of buffering action that neither require nor deliver any knowledge about the individual components, and many different combinations of buffering vessels can bring about identical buffering behavior.

### A formal and general definition of the approach

#### Systems of functions as representations of buffering phenomena

The above examples of systems of communicating vessels (Figure 2) are useful to become familiar with our approach to the quantitation of buffering action. Indeed, this approach is essentially simple, and the principles illustrated by fluid partitioning between two vessels can be applied immediately to other quantities that distribute between two complementary compartments, for instance to the classical case of H^+ ^or Ca^++ ^ions in their complementary pools of "bound" and "free" ions (*Buffering II*^10^).

On the other hand, these examples can illustrate only a fraction of the things one can do in principle with this formal approach to the quantitation of buffering action. This approach has the potential to provide a common language for all types of buffering phenomena, not just for the few cases mentioned. The universal nature of these measures of buffering action, and their various uses can be appreciated and exploited best when the concept is presented in a pure mathematical form. Herein, our buffering concept resembles other formal frameworks such as probability theory or control theory which are, at the core, of purely mathematical nature; specific examples (e.g. flipping coins or control circuit diagrams, respectively) may illustrate these concepts, but cannnot capture them comprehensively and systematically.

Emphasizing those aspects that help to use this approach as a "mathematical tool", the following paragraphs provide such a systematic framework for the quantitation of buffering action. Herein, combinations of communicating vessels (each with its individual fluid volume depending on the common variable "total fluid volume") are replaced by combinations of purely mathematical functions of a common variable. We need the concepts of "partitioned", "two-partitioned" and "buffered systems", of the "sigma function" and the distinction between "conservative" and "non-conservative" partitioned systems, between "moderation" and "amplification", between "inverting" and "non-inverting" buffering, and between "buffering power" and "buffering capacity".

All the definitions and concepts set up here will be applied to specific buffering phenomena in the accompanying article (*Buffering II*^10^). Some interesting theoretical aspects are presented in the Additional files. They touch on the question "What is buffering?" (as opposed to the question "How can we quantitate buffering?"). It will be shown that the definition of "buffering" can be reduced to a set of axioms in almost exactly the same way as the concept of "probability", and therefore an answer to this question is to be sought on the same spot and with the same mathematical and philosophical approaches.

##### Two-partitioned systems

In a system of two communicating vessels, the individual fluid volume in one vessel could be described as a function of total fluid volume, and the volume in the other vessel by another function of the same total fluid volume. We are thus dealing with two functions of a single common independent variable. More precisely, with an "unordered pair" or a "combination" of functions, inasmuch as the two functions are not in a particular order. A combination of two functions of a common independent variable is termed a "two-partitioned system", or ^2^P in brief. Its two functions are termed "partitioning functions" and denoted π_1 _and π_2_. A two-partitioned system can thus be written ^2^P = {π_1_(x), π_2_(x)}, if we let x represent the independent variable. In the following, both functions are assumed to be continuous and differentiable, and x, π_1_(x) and π_2_(x) are all real valued.

Importantly, in order to use the buffering paradigm in a meaningful and correct way, a two-partitioned system is a necessary and sufficient condition. As a consequence, one can apply the buffering paradigm outside pure mathematics to "real world"-phenomena provided these phenomena are represented mathematically by such a combination of functions.

##### Conservative partitioned systems, and the "sigma function"

The examples above obeyed a conservation law, due to physical or chemical constraints: Fluid distributed into various compartments, but its total volume was constant; H^+ ^ions added into a solution were bound by buffers or by water, but their total number did not change. More generally, if the quantity in question is neither created or destroyed in the process, the total change imposed onto the system equals the sum of the two partial changes. Analogously, in terms of functions, we use the term "conservative partitioned system" to designate a system of partitioning functions whose sum equals the value of the independent variable. That condition, termed "conservation condition", can be written as:

[π_1_(x) + π_2_(x)...+ π_n_(x) ] =  = x.

The "sum" of the individual functions, given by the expression , can be used to define a function σ (termed "sigma function") that lumps together all partitioning functions π_i _of a n-partitioned system:

σ: x → .

Using this sigma function, we can rewrite the "conservation condition" briefly as σ(x) = x. Many important phenomena can be represented and analyzed in terms of a conservative partitioned system. Nonetheless, conservation (in this mathematical sense) is an accidental, not a general feature of partitioned systems.

##### Non-conservative partitioned systems

We thus drop the conservation condition σ(x) = x, and allow σ to be a continuous function of any type. This generalization will turn out to be very useful (*Buffering II*^10^). On the one hand, it allows one to express conservative systems in alternative, "parametric" form. As an example, when one describes bound and free H^+ ^ions (expressed in terms of "moles") as a function of total H^+ ^ions (added for instance as strong acid), one may readily measure strong acid in terms of "grams" or "milliliters", instead of "moles". Then, the aggregate "output" does not equal the "input", or σ(x) ≠ x; this inequality characterizes the system as "non-conservative". More importantly, the concept of non-conservative systems allows us to deal with functional relationships between completely heterogeneous physical quantities, and to apply the buffering concept to this class of phenomena. Examples include the buffering of organ perfusion in the face of variable perfusion pressure, or systems level buffering (*Buffering II*^10^).

**Figure 3:**
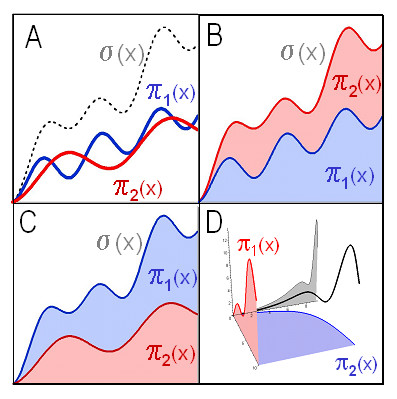
***Graphical representation of two-partitioned systems of functions.*** The unordered combination of two functions π_1_(x), π_2_(x) of a single independent variable x is termed a "two-partitioned system of functions". The two functions may represent the two complementary parts of a whole, e.g. "bound H^+ ^ions" vs. "free H^+ ^ions" in an aqueous solution. The sum of the two functions is termed "sigma function" σ(x) (*see main text for detailed explanation*).. ***A, Family of curves***. The individual functions π_1_(x), π_2_(x), and σ(x) may be plotted individually as a family of curves (this is possible for multi-partitioned systems as well). ***B & C, Area plots. ***The individual partitioning functions of partitioned systems can be plotted "on top of each other" such that the value of each function is represented by the vertical distance between consecutive curves. In a partitioned system, their order is not constrained, and thus two equally valid representations exist for a two-partitioned system (*B,C*). A limitation of area plots is that they do not allow visualization of negative-valued partitioning functions. ***D, Three-Dimensional Space Curve. ***The independent variable x and the values of the partitioning functions π_1_(x), π_2_(x) of a two-partitioned system may be interpreted as x-, y- and z-coordinates, respectively. This results in a three-dimensional space curve. Such a curve can display both positive and negative values. Again, there are two different, equally valid representations. Projections of that curve on the xy-plane (*red*) and xz-plane (*blue*) correspond to the individual partitioning functions π_1_(x) and π_2_(x). Projection of the space curve on the yz-plane (*gray*) corresponds to a plot of the composite relations π_1_(π_2_(x)) or π_2_(π_1_(x)); these projections are not necessarily single-valued functions. The projection on the yz-plane is suited particularly well to assess the proportion between the individual rates of change of the two functions. Importantly, these proportions provide the clue to the quantitation of "buffering action".

Partitioning functions and sigma function can be represented graphically in various ways (Figure 3), e.g. as a family of curves or by an area plot. Moreover, partitioned systems with two partitions π_1 _and π_2 _can be represented by a three-dimensional space curve . For instance, the buffering of H^+ ^ions in pure water or by weak acids is represented as space curve in the accompanying article (*Buffering II*^10^).

##### Buffered systems

In order to talk about buffering with respect to two communicating vessels, it is necessary to decide which vessel would be considered the buffer of the other one. With respect to H^+ ^ions, this assignment is conventionally made in such a way that "free H^+ ^ion concentration" is said to be buffered, and "bound H^+ ^ion concentration" that which brings about buffering. More generally, the two partitioning functions in a two-partitioned system must be assigned two different, complementary roles.

Which is which must be indicated explicitly; here, this shall be done via the particular order: The first partitioning function is taken as description of the quantity that is being buffered, and termed "target" or "transfer function". For clarity, we denote the transfer function by τ(x). The second function is taken as to describe the quantity that brings about buffering, and is termed the "buffering function" β(x). Obviously, two partitioning functions π_1_(x) and π_2_(x) can be arranged in two different ways, with the resulting "ordered combinations" (or "variations") written here {π_1_(x), π_2_(x)} and {π_2_(x), π_1_(x)}. An ordered pair of functions is called a "buffered system". Briefly, a buffered system B can be written B = {τ(x), β(x)}.

#### Quantitative parameters to describe the behavior of buffered systems

**Figure 4:**
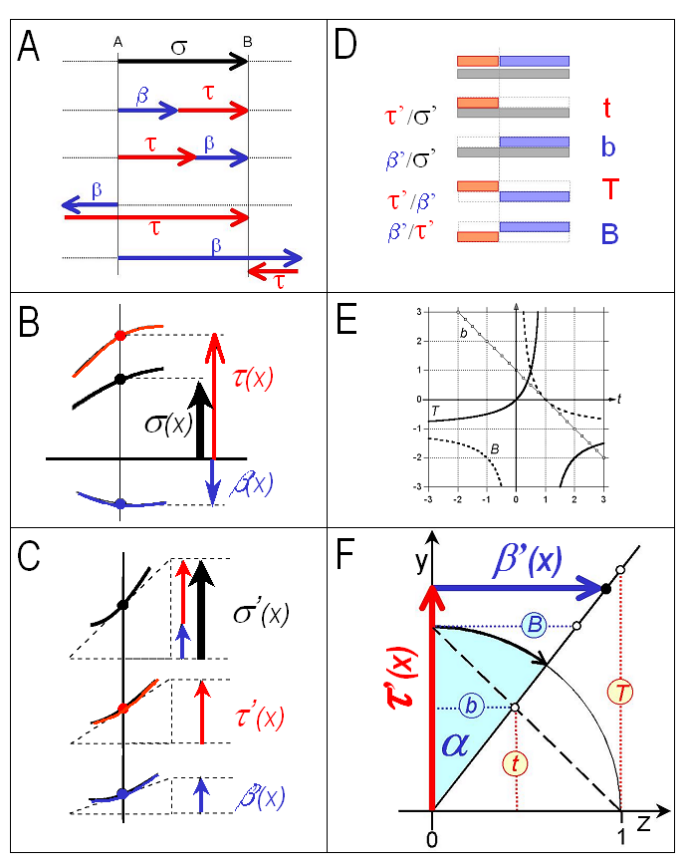
***Proportions in Two-Partitioned Systems.***The buffering measures are dimensionless proportions between two parts of a whole, or between one particular part and the whole. ***A, Bisection of a straight line. ***An oriented line  of length σ (row 1) can be divided in several ways into two parts of lengths τ (red) and β (blue), respectively (rows 2–5). Dividing the line at a point that is lying on the line itself gives rise to an "inner division" (rows 2 and 3), whereas dividing the line outside the interval  yields an "outer divison" (rows 4 and 5). Proportions between the two parts can be expressed in various ways (Figure 4D). For inner divisions, proportions are positive-valued. For outer divisions, "negative proportions" and fractional lengths greater than 1 or smaller than 0 are obtained. ***B, Bisection of a function ***The principle of dividing a quantity into two is also applicable to the values of a function of x at a given value of x. Thus, there are multiple ways to split an entire function σ into two functions τ and β such that the sum of their values τ(x) and β(x) equals the value σ(x) for every x. ***C, Bisection of a slope, or rate of change. ***The quantity to be bisected may as well be the slope σ' of a function σ. Again, there are multiple ways to split a function σ into two functions τ and β such that the sum of their first derivatives τ'(x) and β'(x) equals σ'(x) for every x. For functions and derivatives of functions alike, the proportions between the two parts into which they were split can be expressed by the four measures indicated in Figure 4D. "Buffering" relates to the proportion between such partial rates of change of two complementary processes. ***D, Measures of proportionality between the parts of a bisected slope. ***Proportions among two partial rates of change τ' and β' that result from bisection of a whole rate σ' can be expressed either as fractions of a part with respect to the whole (τ'/σ' and β'/σ') or as as ratio of one part over the other (τ'/β' and β'/τ'). The following terminology is suggested: t, "transfer coefficient"; b, "buffering coefficient"; T, "transfer ratio"; B, "buffering ratio". These parameters serve to quantitate buffering action. ***E, Relation between the four measures of proportion. ***Any single one of the four measures (t,b,T,B) fully determines the other three. The plot shows b, T, and B as functions of t. ***F, Trigonometric measure of proportionality between parts of a bisected slope. ***The two partial rates of change τ'(x) and β'(x) may be interpreted as two perpendicular vectors. Their resultant τ'(x) + β'(x) encloses an "buffering angle α" with τ'(x). The buffering angle and the four buffering parameters (t, b, T, B) are related by four bijections with the buffering angle . A buffering angle α = 0 is equivalent to zero buffering, a buffering angle of 90° to perfect buffering. This representation of buffering behavior does not have discontinuities at "infinite" transfer or buffering odds, and is able to reflect the full range of buffering withing half a unit circle (-45° to 135°). (*See Supplement 5 for further details*)

For every x in an ordered combination of two differentiatable functions τ and β, there are two derivatives τ'(x) and β'(x). The proportions between the two derivatives (i.e., "rates of change") serve to quantitate "transfer" (to the "target compartment") and its complement, "buffering", according to our simple criterion defined above. In general, there are four ways to express the proportions between two parts of a whole (Figure 4). Accordingly, there are four quantitative measures of buffering or transfer in a "buffered system". Herein, we also employ the equivalences y↔τ(x) and z↔τ(x) to facilitate geometrical interpretation in terms of partial derivatives of a space curve (Figure 3D).

##### Transfer coefficient t



Thus, the transfer coefficient expresses the proportion between slope of the transfer function and slope of the sigma function, equivalent to a "fractional" slope or rate of change (taking the slope of the sigma function as "total" slope or rate of change). One might paraphrase the transfer coefficient briefly as the differential . Such a quantity is similar to terms used in specific scientific contexts, such as "yield", "gain", or "compliance".

##### Buffering coefficient b



The buffering coefficient thus expresses, analogous and complementary to the transfer coefficient, a "fractional" rate of change, namely that of the buffering function with respect to the sigma function. Briefly, the buffering coefficient can be paraphrased by the differential . By analogy to the synonyms of the transfer coefficient, one might call the buffering coefficient also "fractional loss" or "uncompliance".

##### Transfer ratio (or transfer odds) T



In words, the transfer odds reflect the proportion between the two complementary slopes or rates of change, or the differential . Possible synonyms roughly matching current buffering terminology are "transfer power" and "transfer strength".

##### Buffering ratio (or buffering odds) B



Thus, the buffering odds similarly reflect the proportion between two slopes or rates of change, but expressed as the inverse of the transfer odds: buffering function slope over transfer function slope, or the differential . Again, possible synonyms corresponding roughly to current buffering terminology are "buffering power" and "buffering strength".

Importantly, the differentials  = b and  = B are useful as measures of buffering action. They are universal and allow one to quantitate both moderation and amplification. Moreover, the buffering odds B yield the desired absolute ratio scale for buffering action.

##### Some properties of the parameters t, b, T, and B

The four parameters are completely interdependent, and any single one of these four parameters completely determines the other three and may be used to express the other ones (Figure 4E and Additional file 3). The parameters t, b, T, and B are defined for positive, negative and zero-values of x, τ(x), β(x), and of the corresponding proportions between their slopes. For space curves (Figure 3D), this property appears trivial. When applied in the context of specific physical sciences, however, this property allows one to describe buffering phenomena that involve negative quantities or zero values. Naturally, this is impossible to achieve with buffering strength units that include logarithmic transforms. In principle, the concept of "buffered systems" can be adapted easily to situations where the system state depends not on one single parameter, but on several of them (Additional File 3).

### Turning the basic approach into a systematic framework

#### Categories of buffered systems

##### Classification according to local behavior

We propose to use the term "buffering" as a general term for all types of behavior of buffered systems. In contrast, the terms "moderation" and "amplification" shall denote specific types of behavior of such systems; namely, "moderation" for buffering with |t(x)| < 1, and "amplification" for buffering with |t(x)| > 1. Furthermore, we can distinguish "inverting buffering" for which t<0, from "non-inverting buffering" for which t>0. Combining these two criteria, one can distinguish "inverting amplification", "inverting moderation", "non-inverting moderation", and "non-inverting amplification". All classical chemical buffers (e.g. for H^+^, Ca^++ ^or other ions) are "non-inverting moderators".

##### Classification according to global behavior

In addition to the classification by local buffering behavior, one may distinguish buffered systems according to their behavior over the entire definition range. Thus, "linear buffers" have transfer and buffering functions that are linear functions of the type τ(x) = a_1_·x + b_1_, and β(x) = a_2_·x + b_2_, where a_1_,a_2_,b_1_,b_2 _are constants. For a linear buffer, the proportion between the two partitions is fixed, and the buffering properties are therefore the same at all states of the system. Examples are the partitioning of fluid between two cylindrical vessels (Figure 2A–D), partitioning of a solute between the two phases in an oil/water emulsion, or taxation according to a fixed tax rate.

"Nonlinear buffers" have nonlinear buffering functions of any other type, and thus the proportion between the two partitioning functions varies with x. Thus, the buffering properties of nonlinear buffers depend on the system state, or the value of the independent variable. Examples include the partitioning of fluid between two irregularly shaped vessels (Figure 2E–F), the buffering of H^+ ^ions by water or by weak acids or bases (*Buffering II*^10^), and taxation according to a progressive tax rate.

##### Buffering capacity vs. buffering power

For the sake of clarity, we would further like to maintain the distinction between *intensity terms *and *capacity terms *in the quantitative description of buffering. As differentials, the parameters t, b, T, and B are intensity terms which describe "fractional rates of change" or "proportions between rates of change". In contrast, a genuine capacity term reflecting an absolute change is obtained by defining a "*buffering capacity*" C_B _as the difference between two particular values z_1 _and z_2 _of the buffering function:

"buffering capacity" C_B _≡ Δz = z_2 _- z_1 _= β(x_2_) - β(x_1_).

A "transfer capacity" C_T _can be defined analogously as the difference between two particular values y_1 _and y_2 _of the transfer function. These "capacities" are either dimensionless numbers, or they are of the same dimension as y and z. For instance, acid-base physiologists and clinicians use the term "total body bicarbonate deficit" (TBBD) in this sense to denote the absolute amount of bicarbonate (indicated in moles or grams) that is needed to increase the present low pH of a patient with metabolic acidosis to the normal value of 7.4.

#### Visualizations: communicating vessels, space curve, buffering angle

Visualizing the elementary partitioning processes that underlie the buffering phenomena not only has great didactic value, but can as well provide a clear and simple representation of buffering phenomena of genuine scientific interest and that are otherwise complicated or abstract; this may help to avoid or to correct misconceptions. Lack of such direct visual equivalents may have contributed to misconceptions and confusion associated with existing buffering strength units in the past [5,6], and to the persisting difficulties of students with that subject.

Fluid-filled communicating vessels can replicate exactly the buffering behavior of virtually all "classical" buffering phenomena, and thus provide visible and tangible "classroom models". In Figure 2, we used this model to illustrate the practical use of the buffering parameters t, b, T, and B. A general method to construct communicating vessel-models of arbitrary buffered systems is described in Additional file 4. The models may be designed in a way that they not only replicate the elementary partitioning process, but also directly visualize buffering strength in terms of the buffering ratio B (or any other buffering parameter, if desired).

Three-dimensional space curves are a more general way to represent two-partitioned and buffered systems graphically (Figure 3D). When such a space curve is projected parallel to the x-axis onto the yz-plane (defined by the two axes that are used to represent τ(x) and β(x), respectively), then a tangent to the curve will enclose a certain angle α with the τ-axis. This "buffering angle" provides another visualization of the proportion between τ'(x) and β'(x) and has useful mathematical properties, analogous to the use of trigonometric representations used in electrical engineering (see Additional file 5 for details, and Figure 4F).

#### Axiomatic foundation

The measures of buffering introduced in this article (t,b,T,B) are essentially proportions between two elements, albeit with some additional specifications: *i*) The elements form an ordered pair; *ii*) The elements and their proportions are not fixed, but may vary as a function of some independent variable; and *iii*) The elements are rates of change (here: derivatives of differentiable functions). Figure 4 illustrated some basic aspects of proportions, including "negative proportions", and various ways to represent such proportions.

In a way, our concept is thus a "play" on proportions, and like any game, it is played in accordance with certain rules. Here, elements and rules are purely mathematical objects. For theoreticians, this situation is an invitation to build the buffering concept from scratch on a minimal set of postulates or axioms. For scientific practitioners, motivation to found the concept of buffering on axioms may spring from the experience that seemingly diverse phenomena may be governed by identical principles, and that these more abstract principles allow all of them to be handled with a single mathematical tool. In this sense, Additional file 6 is an attempt to demonstrate the common principle behind "buffering", "partitioning", and "probability" in an intuitive way, and to point out the desired properties of an axiomatic formulation of these principles.

Additional file 7 then presents such an axiomatic formulation of "buffering" or "partitioning" or "probability". These axioms represent the most concise, definitive, and versatile version of our buffering concept. For many readers, it will also offer the most direct approach, especially if they are already familiar with Kolmogorov's axiomatic foundation of a probability measure.

Importantly, the axiomatic foundation makes the theoretical concept more powerful: Firstly, stringent formalization allows one to decide definitively whether the concept is logically consistent and complete. Secondly, axioms represent the concept in its most general form, and this form is most likely to stimulate free (and correct) use in very diverse, sometimes unanticipated contexts. Moreover, systems of continuous functions are adequate only for a macroscopic description of buffering phenomena. On a microscopic scale, the continuum hypothesis ceases to apply, whereas the quantitative principles that govern these phenomena remain the same. The axioms therefore also provide for systems of discrete functions.

Finally, the axiomatic form exposes the striking formal similarity between the concepts of "probability" and of "buffering" (detailed in Tables 3 and 4 of Additional file 7). This similarity raises deep questions -remaining to be explored- regarding both the interpretation and the mathematical foundation of "probability".

#### Interconversions and practical rules

The general form of the axioms allows one to identify several "technical" aspects of buffered systems that are practically relevant. Firstly, there exist equivalencies with respect to buffering properties between systems that present initially in very different forms. Understanding these equivalencies and being able to interconvert such systems allows one to reduce complexity (Additional file 8). Secondly, buffering phenomena can be formalized as partitioned and buffered systems in more than one, formally correct way. The various options at this step allow one to choose the most suitable formalization, and point to some efficient experimental approaches (Additional file 9).

#### Practical applications

Practical relevance and use of the concept are worked out in the accompanying paper (*Buffering II*^10^), where we apply our definitions and units to various buffering phenomena of genuine scientific interest. These analyses offer some fresh though compelling looks on "classical" buffering phenomena (H^+ ^buffering by pure water or by solutions of weak acids/bases), and demonstrate that our concept affords rigorous quantitative treatment of "non-classical" buffering phenomena for which useful measures of buffering strength have been unavailable so far (redox buffering and blood pressure buffering). Finally, a generalization opens the concept to non-stationary systems and thus allows one to quantitate time-dependent buffering, or "muffling", and "systems level buffering" in an equally rigorous manner.

## Discussion

*The introduction of suitable abstractions is our only mental aid to organize and master complexity. *– Edsger W. Dijkstra

In this article, we introduced quantitative measures of buffering action based on a purely mathematical concept of "buffering". The nucleus of the concept was to describe partitioning processes by means of the proportions between partial and total "changes" or "flows". On this basis, we could define four interrelated, dimensionless measures: transfer coefficient t, buffering coefficient b, transfer ratio T, and buffering ratio B. Together, they allow one to quantitate the behavior of buffered systems in a way that is analogous to the quantitation of chance using "probabilities" and "odds". The magnitude of buffering action may thus be measured using the "buffering coefficient" which provides a relative scale normalized to 1. Alternatively, one may use the "buffering ratio" in order to quantitate buffering action by means of an absolute scale with equal intervals and an absolute zero, the highest scale type possible.

"Buffering" according to this definition turned out to be an entirely mathematical concept. Phenomena encountered in the "real world" may or may not be related to this mathematical concept in exactly the same way in which phenomena may or may not be related to mathematical concepts in general. The concept of exponential decay, for instance, can be stated in purely mathematical terms, but is also exhibited in more or less perfect form by several natural phenomena, such as decaying radioisotopes or chemical reactions on their way to equilibrium. Moreover, our mathematical concept of buffering can also describe amplification phenomena, just as the concept of exponential decay can seamlessly turn into a concept of exponential growth simply by allowing for exponents greater than one.

This section discusses the intrinsic, formal properties of the concept. Some of its – sometimes hidden – connections to previous work, especially to ideas of Henderson, Van Slyke, or Neher & Augustine are exposed and discussed in Additional file 10. Kolmogorov's axiomatic system of probability has been covered extensively elsewhere (see literature in [7,8]). The technical and theoretical implications of our "Non-Kolmogorov probability measure" require further study, but cannot be worked out here. Detailed treatments of specific buffering phenomena are presented in the accompanying paper (*Buffering II*^10^).

### Properties and significance of the general, formal approach to the quantitation of buffering action

The introduction and Additional file 2 listed a number of major problems associated with the present approaches to the quantitation of buffering action. Our formal, general approach and the four buffering parameters t, b, T, and B, provide a theoretically rigorous and practically useful solution to these problems.

#### A ratio scale for buffering strength

The buffering odds B provide an absolute, dimensionless ratio scale for buffering action. This is the highest possible type of scientific scale. The advantages of ratio scales, i.e., equal interval scales with an absolute zero, have been pointed out in the introduction and in Additional file 2. Our buffering strength scale is free of scale artefacts and can be used accurately, not just approximately, for small and large, positive and negative values alike. A further benefit gained with a ratio scale is the possibility to build simple, intuitive models of buffering, e.g. with communicating vessels. Of the previous units for buffering strength, only Neher & Augustine's "Ca^++ ^binding ratio κ_s_" yields a ratio scale.

#### A universal scale for buffering strength

Our definitions of buffering and of measures to quantitate buffering are purely formal, mathematical ones, and the measures t, b, T, and B are all dimensionless numbers. Therefore, this conceptual framework is generally applicable and not arbitrarily limited to buffering phenomena of a particular chemical or physical nature. The concept allows one to handle all classical examples of buffering, such as Ca^++ ^or H^+ ^buffering. In addition, it can be applied to multiple further buffering phenomena encountered in chemistry, biology, physiology, or elsewhere. Application of our concept to familiar examples shows that it is valid, i.e., it reflects faithfully what is qualitatively understood by the word "buffering", and that this will hold generally, not only when certain boundary conditions are met.

We stress the formal aspect of buffering: "Buffering" is a quantitative pattern abstracted from "real" things, not a real thing itself. Therefore, this pattern can be employed freely and in various ways as a tool for the quantitative description of certain aspects of reality. Whether something is the buffer or that which is being buffered is a matter of perspective and thus determined by the analyst, not by reality. Therefore, the definition of a buffer is an operational one: Something that is expressed in terms of a buffering function β constitutes, by definition, a buffer. Conversly, something that cannot be measured by these units cannot and should not be described in terms of buffering terminology. Such an operational definition requires a general, formal concept. In contrast, buffering strength units that are specific for H^+^, Ca^++^, or other particular entities can hardly be wrought into a convincing operational definition of buffering that is not overly exclusive and limited.

#### A scale for moderation and amplification

The mathematical notation of our buffering concept makes it easy to recognize the common pattern behind "moderation" and "amplification", and to accommodate this union formally. A single unit is sufficient to deal with both, and such a unit is also called for frequently when both types of buffering behavior occur in a single system. The association of "buffering" exclusively with "attenuation" or moderation, and the resulting mental divide with respect to the treatment of attenuation vs. amplification phenomena may be related to the historical roots of the buffering concept in acid-base chemistry where "moderation" is the most prominent finding. Overcoming that divide greatly enhances the usefulness of the buffering concept. For instance, we can connect our buffering concept directly to systems and control theory, as detailed in the accompanying article (*Buffering II*^10^). This link can enrich systems and control theory by providing a currently lacking rigorous definition of "systems level buffering" and an accompanying unit to measure this quantity. On the other hand, this link can expand the application range of the buffering concept to all objects and phenomena already studied by systems and control theory. Most importantly, our approach brings together conceptually and technically "buffering" as a homeostatic mechanism, and control theory as the dominant formal language for the description of homeostasis in physiological systems.

#### A standard scale for buffering strength?

The parallel use of multiple, incommensurate scales for buffering strength has engendered ambiguous terminology, misunderstandings and pseudo-problems. Homonymic usage of the term "buffering" might be avoided by agreeing on a certain convention. Ideally, such a convention should codify not just any usage, but the one that affords the highest possible type of scale. Furthermore, a standard scale should cover all possible cases of buffering, and should not arbitrarily exclude some of them. Our concept constitutes such an all-purpose yardstick for buffering strength. It is a valid measure of buffering, universally applicable, and yields a scale of the highest possible type. No other buffering strength unit satisfies these requirements.

Moreover, it is also hard to imagine a more compelling, less arbitrary standard scale than an "absolute ratio scale" as provided by the buffering odds B: The numerical value of the buffering odds B is completely determined by the general definition of B as the ratio of two derivatives and does not depend on any arbitrary scaling factors or units.

In contrast, for instance, the definition of the unit β_H+ _= *d*Base/*d*pH includes a number of additional, unnecessary "rules" that need to be observed in order to obtain the correct value: *i*) Express the concentration of strong base and of free H^+ ^ions in multiples of Avogadro's number per liter (as opposed to absolute numbers, mass, or others); *ii*) Carry out a numerical transformation of one quantity ([H^+^]_free _into pH), but not of the other ([Strong Base]); *iii*) For the transformation, choose a logarithmic one (as opposed to other transformations, e.g. exponential ones); and *iv*) Use the number 10 as the base in this transform (as opposed to e or any other). These procedures are all mere conventions, not compelling formal constraints or the results of particular scientific givens. Another example is de Levie's redox buffer strength which includes multiplication by a factor 1/ln(10) for sheer convenience [9].

## Conclusion

The quantitation of chance can be achieved cleanly and comprehensively by using either probabilities (of occurrence and non-occurrence of an event) or odds (for and against an event). The analogous measures proposed in this article, namely the coefficients (transfer coefficient t and buffering coefficient b) and ratios (transfer ratio T and buffering ratio B), are suited to serve as standard units for buffering action.

## Supplementary Material

Additional File 1 Current Usage of the "Buffering" Paradigm Outside Acid-Base Chemistry Click here for file

Additional File 2 Problems with the Current Approaches to the Quantitation of Buffering Click here for file

Additional File 3 Properties of the Parameters t, b, T, and B Click here for file

Additional File 4 Constructing Communicating Vessel-Models of Partitioned and Buffered Systems Click here for file

Additional File 5 A Trigonometric Representation of Buffering Behavior: The "Buffering Angle" Click here for file

Additional File 6 From Galton Desk to Communicating Vessels – "Partitioning" as a Common Pattern Behind Probability and Buffering Click here for file

Additional File 7 Axiomatic Foundation of the Formal & General Approach Click here for file

Additional File 8 Conservative and Non-Conservative Partitioned Systems – Equivalences and Interconversions Click here for file

Additional File 9 Some Useful General Principles Regarding the Practical Application of the Formal & General Buffering Concept Click here for file

Additional File 10 Historical Note: Origins of the Formal & General Approach Click here for file
