# Supplementary material for: The quantitation of buffering action I. A formal & general approach
Source: Theor Biol Med Model. 2005 Mar 15;2:8. doi: 10.1186/1742-4682-2-8 (PMC1079953; doi:10.1186/1742-4682-2-8)
Supplement: Additional File 1 — Current Usage of the "Buffering" Paradigm Outside Acid-Base Chemistry [file 1742-4682-2-8-S1.pdf]

Theoretical Biology and Medical Modelling

Research

The quantitation of buffering action. I. *A formal and general approach.*

Bernhard M. Schmitt

Supplement 1:

Current usage of the "buffering" paradigm outside acid-base chemistry

Buffering of electrolytes

Similar to H^+ ions, several other electrolytes are stabilized or "buffered" in their respective body fluid compartments, including cytoplasmic calcium [1] and magnesium [2-4]. The definitions of buffering, cast originally in acid-base terminology, were adapted readily to these two ion species. Another important ion associated with buffering is potassium [5-7], even though the physico-chemical processes involved differ profoundly from H^+, Ca^{++} and Mg^{++} buffering, and their elucidation remains a challenge to researchers. Further biologically relevant ions that are buffered include phosphate [8], molybdate [9], iron [10], and ADP/ATP [8,11]. Interestingly, buffering also seems to participate in shaping the spatial and temporal concentration profile of signalling molecules, e.g. those of inositol-1,4,5-trisphosphate [12], or those of neuro-transmitters by binding to "decoy receptors" [13-15]. The buffering of ions is also an important aspect in aquatic, atmospheric and geochemistry. Besides H^+, buffering in these fields was studied for Ca^{++}, and Mg^{++} ions [16], sulfide [17], or phosphate [18].

Buffering of non-electrolyte solutes

An example for an uncharged molecule for which buffering plays an important physiological role is oxygen, with hemoglobin and myoglobin as its major buffers within red blood cells and myocytes, respectively. The fundamental importance of hemoglobin in oxygen transport is well known, including the remarkable affinity switch between source and sink compartments that enhances net transport. The star-nosed mole provides a further example: This small diving mammal exploits oxygen buffering and aerobic metabolism for diving, rather than relying on H^+ buffering and anaerobic glycolysis [19]. An important role for oxygen buffering by myoglobin was also found in a modeling study on capillary networks of aerobic muscles: its contribution to oxygen transport and oxygenation on the tissue level outstripped by far that of vasomotion [20]. Buffering against the cholesterol-raising action of dietary cholesterol could be attributed to a hepatic enzyme, 3-hydroxy-3-methylglutaryl coenzyme A (HMG-CoA) reductase [21]. Buffering against the influx of fat by healthy adipose tissue, but not by

adipose tissue in insulin-resistance was claimed to prevent excessive exposure of other tissues to this influx [22].

Buffering of thermodynamic and hydraulic quantities

A step away from simple solute concentrations, the terms "redox buffering" or "oxidant buffering" were used in biochemistry, pathology, and geochemistry to denote the relative stability of the redox potentials in the face of added reducing or oxidizing equivalents [23-27]. In the context of metabolic control, the thermodynamic potential of intermediates in energy metabolisms was found to be stabilized by "thermodynamic-buffer enzymes" [28], by "energy buffers" that could be deliberately introduced into transgenic plants [29], or by phosphagen systems conveying a "metabolic capacitance" [8].

The term buffering is also associated frequently with hydraulic phenomena such as "blood pressure buffering". By some, this term is used more or less synonymously with "autoregulation" for the stabilization of organ blood flow [30,31]. By others, "blood pressure buffering" is employed in the sense of "blood pressure *variability* buffering" in the face of external or internal disturbances; this has become a popular paradigm in cardiovascular physiology [31-37].

In synovia surrounding joints, hyaluronan chains in the interstitial spaces were found to modify the pressure-flow relationship in a way described as "outflow buffering" of synovial fluid [38,39]. In the plant kingdom, polysaccharide hydrocolloids in leaves were reported to "buffer" leaf water status against environmental fluctuations, due to their high water-binding capacity and ability to act as hydraulic capacitors [40].

Systems level buffering

In evolutionary biology, a recent concept is "phenotypic" or "genetic buffering": The effect of genetic variations on phenotype – and thus on fitness and selection - is minimized or completely intercepted by mechanisms such as redundancy or negative feedback; limitedness of genetic buffering capacity gives rise to threshold behavior and complex patterns of evolutionary stasis and change [41-43]. In the social sciences, "social buffering", "cognitive buffering", or similar hypothetical mechanisms were proposed and studied. According to these hypotheses, the harmful effects of various forms of stress on mental or physical health may be attenuated by social support, cognitive processes, or other factors [44-47].

References

1. RF Abercrombie, CE Hart: **Calcium and proton buffering and diffusion in isolated cytoplasm from Myxicola axons.** *Am J Physiol* 1986, **250:** C391-C405.

2. H Westerblad, DG Allen: **Myoplasmic free Mg^{2+} concentration during repetitive stimulation of single fibres from mouse skeletal muscle.** *J Physiol* 1992, **453:** 413-434.

3. PA Tessman, A Romani: **Acute effect of EtOH on Mg^{2+} homeostasis indf liver cells: evidence for the activation of an Na^{+}/Mg^{2+} exchanger.** *Am J Physiol* 1998, **275:** G1106-G1116.

4. T Gunther, J Vormann, JA McGuigan: **Buffering and activity coefficient of intracellular free magnesium concentration in human erythrocytes.** *Biochem Mol Biol Int* 1995, **37:** 871-875.

5. SN Skatchkov, J Krusek, A Reichenbach, RK Orkand: **Potassium buffering by Muller cells isolated from the center and periphery of the frog retina.** *Glia* 1999, **27:** 171-180.

6. L Vargova, A Chvatal, M Anderova, S Kubinova, D Ziak, E Sykova: **Effect of osmotic stress on potassium accumulation around glial cells and extracellular space volume in rat spinal cord slices.** *J Neurosci Res* 2001, **65:** 129-138.

7. AR Gardner-Medwin: **A new framework for assessment of potassium-buffering mechanisms.** *Ann N Y Acad Sci* 1986, **481:** 287-302.

8. WR Ellington: **Evolution and physiological roles of phosphagen systems.** *Annu Rev Physiol* 2001, **63:** 289-325.

9. AW Schuttelkopf, JA Harrison, DH Boxer, WN Hunter: **Passive Acquisition of Ligand by the MopII Molbindin from Clostridium pasteurianum. Structures of APO and oxyanion-bound forms.** *J Biol Chem* 2002, **277:** 15013-15020.

10. V Picard, S Epsztejn, P Santambrogio, ZI Cabantchik, C Beaumont: **Role of ferritin in the control of the labile iron pool in murine erythroleukemia cells.** *J Biol Chem* 1998, **273:** 15382-15386.

11. A Michailova, A McCulloch: **Model study of ATP and ADP buffering: transport of Ca2+ and Mg2+, and regulation of ion pumps in ventricular myocyte.** *Biophys J* 2001, **81:** 614-629.

12. EA Finch, GJ Augustine: **Local calcium signalling by inositol-1,4,5-trisphosphate in Purkinje cell dendrites.** *Nature* 1998, **396:** 753-756.

13. JM Gershoni, A Aronheim: **Molecular decoys: ligand-binding recombinant proteins protect mice from curarimimetic neurotoxins.** *Proc Natl Acad Sci USA* 1988, **85:** 4087-4089.

14. CF Ware: **Decoy receptors thwart B cells.** *Nature* 2000, **404:** 949-950.

15. A Mantovani, M Locati, A Vecchi, S Sozzani, P Allavena: **Decoy receptors: a strategy to regulate inflammatory cytokines and chemokines.** *Trends Immunol* 2001, **22:** 328-336.

16. K Hyeong, RM Capuano: **Ca/Mg of brines in Miocene/Oligocene clastic sediments of the Texas Gulf Coast: buffering by calcite/disordered dolomite equilibria.** *Geochim Cosmochim Acta* 2001, **65:** 3065-3080.

17. SK Heijs, H van Gemerden: **Microbiological and environmental variables involved in the sulfide buffering capacity along a eutrophication gradient in a coastal lagoon (Bassin d'Arcachon, France).** *Hydrobiologia* 2000, **437:** 121-131.

18. Y Sui, ML Thompson: **Phosphorus sorption, desorption, and buffering capacity in a biosolids-amended mollisol.** *Soil Sci Soc Am J* 2000, **64:** 164.

19. IW McIntyre, KL Campbell, RA MacArthur: **Body oxygen stores, aerobic dive limits and diving behaviour of the star-nosed mole (Condylura cristata) and comparisons with non-aquatic talpids.** *J Exp Biol* 2002, **205:** 45-54.

20. D Goldman, AS Popel: **A computational study of the effect of capillary network anastomoses and tortuosity on oxygen transport.** *J Theor Biol* 2000, **206:** 181-194.

21. GC Ness, CM Chambers: **Feedback and hormonal regulation of hepatic 3-hydroxy-3-methylglutaryl coenzyme A reductase: the concept of cholesterol buffering capacity.** *Proc Soc Exp Biol Med* 2000, **224:** 8-19.

22. KN Frayn: **Adipose tissue and the insulin resistance syndrome.** *Proc Nutr Soc* 2001, **60:** 375-380.

23. R de Levie: **Redox Buffer Strength.** *JChemEd* 1999, **76:** 574-577.

24. ES Jacobson, JD Hong: **Redox buffering by melanin and Fe(II) in Cryptococcus neoformans.** *J Bacteriol* 1997, **179:** 5340-5346.

25. GJ Van Berkel, V Kertesz: **Redox buffering in an electrospray ion source using a copper capillary emitter.** *J Mass Spectrom* 2001, **36:** 1125-1132.

26. E Mosharov, MR Cranford, R Banerjee: **The quantitatively important relationship between homocysteine metabolism and glutathione synthesis by the transsulfuration pathway and its regulation by redox changes.** *Biochemistry* 2000, **39:** 13005-13011.

27. J Kefer, A Rahman, KN Anwar, AB Malik: **Decreased oxidant buffering impairs NF-kappaB activation and ICAM-1 transcription in endothelial cells.** *Shock* 2001, **15:** 11-15.

28. JW Stucki: **The thermodynamic-buffer enzymes.** *Eur J Biochem* 1980, **109:** 257-267.

29. J Farres, N Holmberg, U Schlattner, JE Bailey, T Walllimann, PT Kallio: **Expressing creatine kinase in transgenic tobacco - a first step towards introducing an energy buffering system in plants.** *Transgenic Research* 2002, **11:** 49-59.

30. SJG Semple, HE De Wardener: **Effect of increased renal venous pressure on circulatory "autoregulation" of isolated kidney.** *Circulation Research* 1959, **7:** 643-648.

31. J Jordan, HR Toka, K Heusser, O Toka, JR Shannon, J Tank, A Diedrich, C Stabroth, M Stoffels, R Naraghi et al.: **Severely impaired baroreflex-buffering in patients with monogenic hypertension and neurovascular contact.** *Circulation* 2000, **102:** 2611-2618.

32. BJ Janssen, PJ Leenders, JF Smits: **Short-term and long-term blood pressure and heart rate variability in the mouse.** *Am J Physiol Regul Integr Comp Physiol* 2000, **278:** R215-R225.

33. A Just, U Wittmann, B Nafz, CD Wagner, H Ehmke, HR Kirchheim, PB Persson: **The blood pressure buffering capacity of nitric oxide by comparison to the baroreceptor reflex.** *Am J Physiol* 1994, **267:** H521-H527.

34. B Nafz, CD Wagner, PB Persson: **Endogenous nitric oxide buffers blood pressure variability between 0.2 and 0.6 Hz in the conscious rat.** *Am J Physiol* 1997, **272:** H632-H637.

35. AB Roald, J Ofstad, BM Iversen: **Attenuated buffering of renal perfusion pressure variation in juxtamedullary cortex in SHR.** *Am J Physiol Renal Physiol* 2002, **282:** F506-F511.

36. RP Sloan, PA Shapiro, E Bagiella, MM Myers, JM Gorman: **Cardiac autonomic control buffers blood pressure variability responses to challenge: a psychophysiologic model of coronary artery disease.** *Psychosom Med* 1999, **61:** 58-68.

37. JA Taylor, DL Eckberg: **Fundamental relations between short-term RR interval and arterial pressure oscillations in humans.** *Circulation* 1996, **93:** 1527-1532.

38. PJ Coleman, D Scott, RM Mason, JR Levick: **Role of hyaluronan chain length in buffering interstitial flow across synovium in rabbits.** *J Physiol* 2000, **526 Pt 2:** 425-434.

39. D Scott, PJ Coleman, RM Mason, JR Levick: **Concentration dependence of interstitial flow buffering by hyaluronan in synovial joints.** *Microvasc Res* 2000, **59:** 345-353.

40. SC Clifford, SK Arndt, M Popp, HG Jones: **Mucilages and polysaccharides in Ziziphus species (Rhamnaceae): localization, composition and physiological roles during drought-stress.** *J Exp Bot* 2002, **53:** 131-138.

41. JL Hartman, B Garvik, L Hartwell: **Principles for the buffering of genetic variation.** *Science* 2001, **291:** 1001-1004.

42. M Pigliucci: **Developmental Genetics: Buffer zone.** *Nature* 2002, **417:** 598-599.

43. SL Rutherford: **From genotype to phenotype: buffering mechanisms and the storage of genetic information.** *Bioessays* 2000, **22:** 1095-1105.

44. JL Anthony, WH O'Brien: **An evaluation of the impact of social support manipulations on cardiovascular reactivity to laboratory stressors.** *Behav Med* 1999, **25:** 78-87.

45. JS Gillis: **Stress, anxiety, and cognitive buffering.** *Behav Med* 1992, **18:** 79-83.

46. R Olstad, H Sexton, AJ Sogaard: **The Finnmark Study. A prospective population study of the social support buffer hypothesis, specific stressors and mental distress.** *Soc Psychiatry Psychiatr Epidemiol* 2001, **36:** 582-589.

47. A Steptoe: **Stress, social support and cardiovascular activity over the working day.** *Int J Psychophysiol* 2000, **37:** 299-308.
