# Supplementary material for: The quantitation of buffering action I. A formal & general approach
Source: Theor Biol Med Model. 2005 Mar 15;2:8. doi: 10.1186/1742-4682-2-8 (PMC1079953; doi:10.1186/1742-4682-2-8)
Supplement: Additional File 2 — Problems with the Current Approaches to the Quantitation of Buffering [file 1742-4682-2-8-S2.pdf]

Theoretical Biology and Medical Modelling

Research

The quantitation of buffering action. ***I. A formal and general approach.***

Bernhard M. Schmitt

Supplement 2:

Problems With Current Approaches to the Quantitation of Buffering Action

The paradigm of "buffering" is applied to multiple phenomena of diverse nature. Similarly, a great variety of units for the quantitation of buffering action has been proposed. Fundamental aspects regarding the definition and quantitation of "buffering" remain unclear or unresolved. *i)* Is there a single common principle behind these diverse buffering phenomena? Or is "buffer" an equivocal label for different things that resemble only superficially? *ii)* If there is a common principle, why isn't there a single common scale?

Analogously to other scales, such a scale for buffering strength would provide an *operational definition* of the quantity "buffering strength": Anything that can be assigned a unique position on the scale *is* -by definition- genuine buffering; and reversly, anything that does not fit on the scale is not buffering.

Put differently: If a given phenomenon does not fit on any of the presently available scales, is this because the phenomenon does not constitute genuine buffering, or because the scales are defined too narrowly and simply fail to cover all possible cases of genuine buffering? *iii)* For those phenomena for which multiple competing buffering strength units exist: Do they really all measure the same quantity? Are they equally powerful scientifically, or are there superior and inferior ones?

Need for a consensus regarding the quantitation of buffering action

A clear and uniform understanding of the precise meaning of the terms "buffering" and "buffering strength" is required in order to formulate, treat and discuss such issues scientifically. The lack of such a consensus causes problems, or actually "pseudo-problems" (i.e., misunderstandings on a semantic level, as opposed to the lack of knowledge with respect to the study object). This is illustrated by a recent controversy over the question whether cytoplasmic buffering power increases or decreases with increasing pH

[1,2]. Herein, a common and undisputed basis of raw data was interpreted with the aid of two different buffering strength units, which resulted in two internally consistent, yet mutually conflicting conclusions. The conflict was not related to the technical aspects of measuring cytoplasmic pH, but to the way in which the quantity "buffering power" was obtained from these basic data.

Considering such gross formal inconsistencies and ambiguities produced by the current ways to quantitate buffering action, however, *"…it must be stated that the scientific community is unbelievably forgiving"*, as E. Neher stated in a similar context [3].

Numerous and fundamental aspects of buffering cannot be discussed or decided at present for lack of a single, common definition and scale for buffering strength. Examples are the characterization of individual buffers (e.g. *"At which pH does a weak acid buffer most strongly?"*; see accompanying paper); comparisons between different types of buffering (e.g. *"Is the concentration of free magnesium ions buffered more or less strongly than redox potential?"*); the quantitation of H^+ buffering strength in non-aqueous acid-base systems where the pH scale is not applicable (e.g. *"How strong is H^+ buffering in a pure NH_4^+/NH_3 solution?"*); or the quantitation of buffering strength where units are simply unavailable (e.g. *"How strongly is perfusion of the kidney buffered against blood pressure changes, and how does this compare to blood pressure buffering in other organs?"*).

Systematic criticism of present buffering strength units

Buffering strength units that are incommensurate detect distinct quantities

Instead of a single universally accepted unit, multiple "buffering strength" units exist, many of which are applied to identical phenomena. An impressive example of this redundancy is the buffering of various electrolytes in solution *(see Table 1 in this Supplement)*. This multiplicity differs from that of other scientific units, e.g. for mass (grams, pounds, ounces, stones etc.) insofar as these different buffering strength units cannot be interconverted simply by rescaling (i.e., multiplication with a scaling factor and, if necessary, addition of a constant). In other words, some buffering strength units are mutually "incommensurate".

As a consequence, they will yield quantitatively and qualitatively conflicting descriptions of identical phenomena *(see Table 1)*. For instance:

i) Buffering strength is a positive quantity with some units (e.g., van Slyke's unit β_{H^+} and Neher & Augustine's unit κ_s), and a negative quantity with others (Koppel & Spiro's unit P, Stewart's unit $\Delta[SID]/\Delta[H^+]$).

ii) Moving from "weaker" to "stronger" buffering increases the numerical value of buffering strength according to some units (β_{H^+}, κ_s), but decreases it for others (P, $\Delta[SID]/\Delta[H^+]$, $d[Ca^{++}]_{free}/d[Ca^{++}]_{bound}$).

iii) A given change may produce a linear increase of buffering strength with one unit (e.g., κ_s), a logarithmic change using another (e.g., $dCa^{++}/dpCa^{++}$), and a hyperbolic one in a third (e.g., $d[Ca^{++}]_{free}/d[Ca^{++}]_{total}$; $1\text{-}d[H^+]/d[Acid]$).

iv) Finally, the value of buffering strength should remain constant if buffering remains constant. This behavior is not observed in all cases, however. For instance, in the complete absence of any buffering, the buffering strength β_{H^+} assumes a value of $\beta_{H^+} = \ln(10)\times[H^+]$ rather than of zero. Under such conditions, this measure of buffering strength has lost any correlation with buffering strength proper, but depends instead linearly and completely on $[H^+]$. The unit β_{H^+} then blatantly fails to reflect what is commonly understood by the word "buffering".

The significance of this incommensurability is that incommensurate buffering strength units clearly do not measure the same thing.

Buffering strength units that have different physical dimensions detect different quantities

Similar conclusions follow independently from a dimensional analysis of the various buffering strength units. Some units are dimensionless (e.g. $dAcid/d[H^+]$ or $d[Ca^{++}]_{free}/d[Ca^{++}]_{total}$ while others have the dimension of moles/(liter×pH) (e.g. dBase/dpH). If two units are of different physical dimensions, however, they can impossibly

be measures of the same quantity. One may fail to appreciate the severity of this inconsistency because all these measures depend on and change in some way with buffering strength. Nonetheless, such an argument suffers from the basic flaw that it mistakes correlation for identity. Similarly, length vs. surface area are clearly distinct quantities albeit surface area in a rectangle correlates with the lengths of its sides, and surface area vs. capacitance are distinct quantities albeit the capacitance of a capacitor correlates with its surface area.

Now, if two buffering strength units are incommensurable or dimensionally heterogeneous, the conclusion is inescapable that these units measure different things. The one term “buffering” is thus associated in practice with a number of different things, i.e., it is equivocal, or homonymic. Unequivocal terminology, however, has been an undisputed cornerstone of science, first seen clearly by Aristotle in his *“Analytica posteriora”*.

On the other hand, such lexical specificity is hardly trivial, as is apparent from ongoing debates over other scientific terms such as “species”, “epitope”, or “life” [4-7]. In our context, resolving the mentioned controversy whether buffering strength increases or decreases with pH would primarily require to overcome the lack of semantic specificity of the term “buffering strength” rather than collecting data.

Most buffering strength units yield scales of low scientific standard.

In addition to the criterion of mutual commensurability or incommensurability, scientific scales can be categorized and ranked according to their intrinsic qualities. A common and useful classification by Stevens distinguishes nominal scales, ordinal scales, interval scales, and ratio scales [8].

The most powerful and versatile type of scale is the „ratio scale“, defined as an „equal interval scale“ with an „absolute zero“. Anything that can possibly be done with scales can be done with ratio scales: ranking of values, comparing intervals, adding, subtracting, or averaging, and comparing values by means of ratios or percentages. Other scale types may be more familiar or yield simpler numbers, but allow fewer operations than ratio scales and are therefore less powerful scientifically. Usually, however, ratio scales also offer the most intuitive approach: if there is nothing of the quantity, the value is zero; if there is something, the value is some positive number; if there is more, the value is greater; if the quantity changes by a certain factor, the value changes by that same factor.

Such a behavior is not as trivial as it may seem, and in fact most of the units shown in *Table 1* do not behave in this way. The one notable exception is the “calcium binding capacity” κ_s *(Table 1 of this Supplement, and Buffering I - Supplement 10).*

Equal interval scales

More specifically, the first feature of ratio scales is that of “equal intervals” on the scale for equal increments of the quantity to be measured. This property is obvious in familiar units such as meter, centigrade, or moles per liter. An equal interval scale is the prerequisite for addition, subtraction, and averaging.

Among the buffering strength units, only the calcium binding ratio κ_s provides an equal interval scale in the strict sense. Moreover, Stewart’s $\Delta[SID]/\Delta[H^+]$ and Van Slyke’s unit $\Delta[Acid]/\Delta[H^+]$ become practically equivalent to equal interval scales under conditions of “strong” buffering. For “weak” or completely absent buffering, however, these units approach a value of 1 rather than 0 and become highly non-linear. Problems with these scales are therefore particularly apparent with weakly buffered species such as Mg^{++}. Furthermore, Westerblad & Allen’s unit, or the unit $1\text{-}d[H^+]/d[Acid]$ of Van Slyke are buffer strength units with non-equal interval scales.

Non-equal intervals are particularly conspicuous with logarithmic scales, such as the Richter scale for earthquakes, the decibel scale for sound intensity, and those buffering strength units that are derived from the pH scale (Van Slyke’s unit β_{H^+} or Koppel & Spiro’s unit P).

Scales with an absolute zero

As a second essential feature, ratio scales have an "absolute zero". This means that the absence of the quantity in question corresponds to a value of zero on the scale.

Quantities measured by an equal interval scale can only be compared relative to each other if the scale has an absolute zero. For instance, *"200 °Kelvin is twice as warm as 100 ° Kelvin"* is a meaningful statement, whereas *"200 °Fahrenheit is twice as warm as 100 °Fahrenheit"* is nonsensical. Buffering strength units with an absolute zero include Koppel & Spiro's unit P, the unit 1-$d[H^+]/d[Acid]$ of Van Slyke, and Neher & Augustine's unit κ_s.

In contrast, the buffering strength unit $dAcid/d[H^+]$ will not assume the value of zero in the complete absence of H^+ buffering, but a value of 1. Similarly, the unit $\beta_{H^+}=dBase/dpH$ does not have an absolute zero, but rather assumes the value of $\beta_{H^+}=\ln(10)\times[H^+]$ in the absence of buffering. These properties stem from the properties of the pH scale which does not have an absolute zero itself, rendering unable to properly express the absence of H^+ ions.

The distortion of β_{H^+} by the term $\ln(10)\times[H^+]$ in the proximity of zero buffering has rather unfavorable consequences: When weak buffering phenomena are recorded at different pH values and compared, this scale may fail even at the simple task of ranking them correctly according to buffering strength. Strictly speaking, van Slykes unit β_{H^+} does thus not meet the requirements even for an ordinal scale.

It follows that of the existing measures for buffering strength, only the "calcium binding ratio κ_s" of Neher & Augustine allows one to build a ratio scale. It will become evident *(see main text)* that the unit κ_s represents a formally equivalent, albeit calcium-specific, version of the general, formal buffering unit presented in this article.

Buffering concepts are too narrow due to definitions containing contingent terms.

Present definitions of buffering, buffering strength, and buffering strength units rely on terms and concepts such as „acid", „base", „pH", or „calcium concentration". Because of these terms, however, the definitions limit themselves to certain subsets of buffering phenomena.

These limitations critically affect more the usefulness of the units rather than their formal correctness. Nonetheless, this limitation is a serious disadvantage: If Newton had stated his concept of universal gravitation in terms of „apples" and „earths", instead of using the more general term „mass" (next to measures for length and time), the concept would have been similarly valid, yet practically useless except for studying dropping apples on this particular planet.

Clearly, nothing is gained but much is lost when units for buffering strength (as well as laws and definitions in the empirical sciences in general) are not stated in the most general form possible. The most general form is that which contains no contingent terms. In this sense, the terms „acid" and "strong base" represent contingent aspects of buffering, because they are involved in some cases of buffering, but not in others (e.g., the buffering of calcium or magnesium ions). The same applies to terms such as „calcium", "magnesium", or any other particular solute of interest that exhibits buffering behavior. Yet even the term „concentration" represents a contingent aspect of buffering *(Buffering II)*.

Buffering strength units do not accommodate amplification.

Finally, suppose we actually had a clear definition of a unit to describe the behavior of systems in which the response to a certain disturbance is „buffered", "moderated", or attenuated, as compared to an unbuffered reference. Then it is hard to see why the same unit could or should not be used when the response to a given disturbance is *greater* than in the unbuffered reference.

A fitting term for such a behavior is "amplification". In fact, amplification is a rather common property of buffered systems, either alone or in combination with moderation.

References

1. AM Saleh, G Rombola, DC Batlle: **Intracellular H^+ buffering power and its dependency on intracellular pH.** *Kidney Int* 1991, **39:** 282-288.
2. DJ Goldsmith, PJ Hilton: **Relationship between intracellular proton buffering capacity and intracellular pH.** *Kidney Int* 1992, **41:** 43-49.
3. E Neher: The use of fura-2 for estimating Ca buffers and Ca fluxes. *Neuropharmacology* 1995, 34: 1423-1442.
4. KL Shaw: **Evolution: Do we need a species concept?** *Science* 2002, **295:** 1238-1239.
5. NS Greenspan: **Wishful thinking and semantic specificity.** *The Scientist* 2002, 12.
6. NS Greenspan: **Epitopes, paratopes, and other topes: Do immunologists know what they are talking about?** *Bulletin of the Institute Pasteur* 1992, **90:** 267-279.
7. DEJr Koshland: **Special essay. The seven pillars of life.** *Science* 2002, **259:** 2215-2216.
8. SS Stevens: **Mathematics, measurement, and psychophysics.** In *Handbook of experimental psychology*. Edited by Stevens SS. New York: Wiley & Sons; 1951:1-49.
9. LJ Henderson: **Concerning the relationship between the strength of acids and their capacity to preserve neutrality.** *Am J Physiol* 1908, **21:** 173-179.
10. DD Van Slyke: **On the measurement of buffer values and on the relationship of buffer value to the disociation constant of the buffer and the concentration of the buffer solution.** *J Biol Chem* 1922, **52:** 525-570.
11. A Roos, WF Boron: **The buffer value of weak acids and bases: origin of the concept, and first mathematical derivation and application to physico-chemical systems. The work of M. Koppel and K. Spiro (1914).** *Respir Physiol* 1980, **40:** 1-32.
12. L Michaelis: *Die Wassserstoffionenkonzentration*, 2 edn. Berlin: Verlag von Julius Springer; 1922.
13. L Michaelis: *Hydrogen Ion Concentration, translated from the 2nd German edition by W.A. Perlzweig*. Baltimore: Willliams and Wilkins; 1926.
14. H Westerblad, DG Allen: **Myoplasmic free Mg^{2+} concentration during repetitive stimulation of single fibres from mouse skeletal muscle.** *J Physiol* 1992, **453:** 413-434.
15. PA Stewart: **Modern quantitative acid-base chemistry.** *Can J Physiol Pharmacol* 1983, **61:** 1444-1461.
16. E Neher, GJ Augustine: **Calcium gradients and buffers in bovine chromaffin cells.** *J Physiol* 1992, **450:** 273-301

Table 1: Multiple, competing units to quantitate buffering action in electrolyte solutions.

Multiple units claiming to represent valid measures of a single quantity, namely of the extent by which the concentration of a particular ion (H^+, Ca^{++}, or Mg^{++}) in a solution is buffered.

First column, original notation by the authors;

Second column, the same measure, rewritten systematically where x stands for total ion concentration, y for free ion concentration, and z for the concentration of bound ion;

Third column, equivalent expression in terms of the four buffering measures introduced in this article, and assuming that the quantity indicated by x is a conserved one, i.e., y+z=x;

Fourth column, physical dimension of the unit;

Fifth column, range of values that the measure can assume (times the physical unit, where required);

Sixth and seventh column, values obtained with the parameter when applied to the H^+ buffering in pure water at extremely acidic and extremely alkaline pH values, respectively;

Eighth and ninth column, predictions obtained with the parameter with respect to position and magnitude, respectively, of H^+ buffering in a solution of a weak acid or base;

Tenth column, references.

Table 1

Parameter	***General Notation***	***tbTB-Equivalent***	***Dimension***	***Range***	***Value in very acidic water***	***Value in very alkaline water***	***Weak acid, maximum at:***	***Weak acid, maximum value:***	***Reference***
$\frac{\Delta[Acid]}{\Delta[H^+]_{free}}$	$\frac{\Delta x}{\Delta y}$	$\sim\frac{1}{t}$	-	$[+1,+\infty]$	+1	∞	$[H^+] = 0$	$\max \propto \left(\frac{A_{tot}}{K_d}\right)$	Henderson (1908) [9]
$\frac{d[Acid]}{d[H^+]_{free}}$	$\frac{dx}{dy}$	$\frac{1}{t}$	-	$[+1,+\infty]$	+1	∞	$[H^+] = 0$	$\max \propto \left(\frac{A_{tot}}{K_d}\right)$	Van Slyke (1922) [10]
$P = \frac{d(S - So)}{dpH}$	$\frac{dx}{d\log(s)} - \frac{dx}{d\log(x)}$	-	M	$[0, +\infty]$	0	0	pH=pKa	$\max \propto A_{tot}$	Koppel & Spiro (1914) [11]
$\beta_{H^+} = \frac{d[Base]}{dpH}$	$\frac{dx}{d\log(y)}$	-	M	$[0, +\infty]$	$+\infty$	$+\infty$	$pH=pK_a$	$\max \propto A_{tot}$	Michaelis (1922) Van Slyke (1922) [12,13]
$\frac{d[Mg^{++}]_{free}}{d[Mg^{++}]_{total}}$	$\frac{dy}{dx}$	t	-	[0, 1]	1	0	$[H^+] \to \infty$	$\min \propto \frac{1}{(A_{tot} \times K_d)}$	Westerbad & Allen (1992) [14]
$\frac{\Delta SID}{\Delta[H^+]_{free}}$	$-\frac{dx}{dy}$	$-\frac{1}{t}$	-	]-∞, -1]	-1	-∞	$[H^+] = 0$	$\max \propto -\left(\frac{A_{tot}}{K_d}\right)$	Stewart (1982) [15]
$1 - \frac{d[H^+]_{free}}{d[Acid]}$	$\frac{dz}{dx}$	$1 - \frac{1}{t}$ =b	-	[0, 1]	0	1	$[H^+] = 0$	$\max = \frac{A_{tot}}{(A_{tot} + K_d)}$	Van Slyke(1922) [10]
$\kappa_s = \frac{d[Ca^{++}]_{bound}}{d[Ca^{++}]_{free}}$	$\frac{dz}{dy}$	B	-	$[0, +\infty]$	0	$+\infty$	$[H^+] = 0$	$\max = \frac{A_{tot}}{K_d}$	Neher&Augustin (1992) [16]
