# Supplementary material for: The quantitation of buffering action I. A formal & general approach
Source: Theor Biol Med Model. 2005 Mar 15;2:8. doi: 10.1186/1742-4682-2-8 (PMC1079953; doi:10.1186/1742-4682-2-8)
Supplement: Additional File 3 — Properties of the Parameters t, b, T, and B [file 1742-4682-2-8-S3.pdf]

Theoretical Biology and Medical Modelling

Research

The quantitation of buffering action. I. ***A formal and general approach.***

Bernhard M. Schmitt

Supplement 3:

Notes on the Parameters t, b, T, and B

The four parameters are completely interdependent.

The four parameters represent the four ways to express the proportion between a single combination of two magnitudes *(main text, Figure 4D and E)*. Thus, any single one of these four parameters completely determines the other three and may be used to express the other ones:

$$\begin{pmatrix} t \\ b \\ T \\ B \end{pmatrix} = \begin{pmatrix} t \\ 1-t \\ t/(1-t) \\ (1-t)/t \end{pmatrix} = \begin{pmatrix} 1-b \\ b \\ (1-b)/b \\ b/(1-b) \end{pmatrix} = \begin{pmatrix} T/(1+T) \\ 1/(1+T) \\ T \\ 1/T \end{pmatrix} = \begin{pmatrix} 1/(1+B) \\ B/(1+B) \\ 1/B \\ B \end{pmatrix}$$

Figure 4E of the main text shows the parameters b, T, and B, plotted as functions of t. Inspection of this graph reveals a linear relationship between t and b. In contrast, T and B depend hyperbolically on t, and there are two values of t where either T or B are not defined, namely at $t=1$ and at $t=0$, respectively.

The four parameters allow for negative quantities and negative rates of change.

The parameters t, b, T, and B are defined for positive, negative and zero-values of x, $\tau(x)$, $\beta(x)$, and of the corresponding proportions between their slopes. For space curves, this property appears trivial *(Figure 3D in the main text)*. When applied in the context of specific physical sciences, however, this property allows one to describe buffering phenomena that involve negative quantities or zero values. Naturally, this is impossible to achieve with buffering strength units that include logarithmic transforms.

Parametrization may turn non-differentiable partitioning functions into differentiable ones, allowing to form buffered systems.

The functions $\tau(x)=y(x)$ and $\beta(x)=z(x)$ must be single-valued functions of x in order to be differentiable; thus, the space curve must not „loop back“ with respect to the direction of the x-axis. This condition does not, however, constrain the other relations [i.e., $y\rightarrow x(y)$, $x\rightarrow x(z)$, $z\rightarrow y(z)$, and $y\rightarrow z(y)$] which are thus not necessarily single-valued functions. On the other hand, transfer or buffering functions that are not differentiable with respect to x may be differentiable with respect to another variable when expressed in a suitable parametric form. For instance, a transfer function $\tau(x)$ that loops back and even intersects itself may be transformed into a parametric function $\tau^*(s)$ of arc length s of the space curve. While the derivatives $\tau'(x)$ and $\tau^{*\prime}(s)$ are not identical, the buffering parameters are identical.

For instance, we find for the buffering ratio B that $B(x) = \tau'(x)/\sigma'(x) = \tau^{*\prime}(s)/\sigma^{*\prime}(s)$. Note that the parametric forms of a conservative partitioned systems usually are non-conservative.

The buffering parameters are invariant under linear transformation of scales or units.

As a ratio of differentials, the four parameters for a given point on the curve are invariant with respect to translation or linear transformation of the coordinates, provided that both the y- and the z-axis are transformed together and in the same way. This property, a corollary to the above statements about the parametric form of buffered systems, is useful both practically and theoretically.

Firstly, when the mathematical concept is applied to physical systems, the values of these parameters do not depend on the particular units used. In contrast, units that include the logarithm of a physical quantity (e.g., Van Slyke's buffering value dBase/dpH) must rely explicitly or tacitly on a standard unit in order to convert the physical quantity into a dimensionless number. Thus, our concept can be applied to buffering phenomena involving any type of quantity. Furthermore, if several equivalent units exist that allow to measure and express that quantity, any single one, e.g. the most convenient one, may be used. For instance, the description of H^+ buffering with these parameters will result in identical descriptions of buffering regardless of whether H^+ ions are measured in moles, moles per liter, grams, ounces, or others.

Secondly, only the derivatives, not the absolute values of $\tau(x)$ and $\beta(x)$ are required to determine a system's buffering behavior. This implies that the space curve may or may not pass through the origin. In other words, it is not required to know in absolute terms the amount of the quantity in question within the system and its individual partitions. It is sufficient to know the *changes* of that quantity as the system moves from one state to another. We will exploit this feature, for instance, in our analysis of H^+ buffering in pure water *(Buffering II)* where it is difficult to indicate the absolute amount of H^+ ions in a given water volume, whereas it is straightforward to indicate by how much this amount changes when strong acid or base are added.

Buffered systems of higher dimensionality, "scalar fields"

In principle, the definition of t, b, T, and B may be extended to buffered systems with transfer and buffering functions that are functions of several independent variables $x_1, x_2 \ldots x_n$. For instance, more than one independent variable are required for a full description of physico-chemical buffering when additional factors affecting the buffering process are taken into account, such as position in space, temperature, pressure, osmolarity, ionic strength or others. Such "scalar fields" lead to partitioning functions, or to transfer and buffering functions that are functions of several variables x_i:

$$y = \tau(x_1, x_2, \ldots, x_n)\ ,$$

$$z = \beta(x_1, x_2, \ldots, x_n)\ .$$

Computing the buffering parameters from partial derivatives

Derivatives $\tau'(x)$ and $\beta'(x)$ may be obtained by keeping all variables constant except for one variable x_k, and thereby effectively turning them into functions of a single variable x_k. The buffering parameters (t, b, T, and B) can then be computed from the *partial* derivatives

$$\frac{\partial}{\partial x_k}\tau(x)$$

and

$$\frac{\partial}{\partial x_k}\beta(x)$$

in the same way as shown above for the total derivatives

$$\frac{d}{dx}\tau(x)$$

and

$$\frac{d}{dx}\beta(x)\ .$$

Computing the buffering parameters from total derivatives

Functions of multiple variables possess well-defined *total* derivatives; these may be used as well to compute the buffering parameters. For a function $f(x)$ of a single independent variable, the derivatives indicated the rate of change of the dependent variable with respect to a change in the direction of the independent variable. For functions $f(x_1, x_2, \ldots, x_n)$ of n variables, the derivative similarly indicates the rate of change of the dependent variable, now for a change in a particular direction within the n-dimensional space. This change is given by a displacement vector

$$e=(e_1\cdot\mathbf{x_1}, e_2\cdot\mathbf{x_2}, \ldots, e_n\cdot\mathbf{x_n}) ,$$

where $\mathbf{x_1}, \mathbf{x_2}, \ldots, \mathbf{x_n}$ are the individual components of the n-dimensional unit vector. To specify the point for which the buffering parameters are to be calculated, a single x value does not suffice here; rather, the values of all independent variables x_i are needed. These can be indicated by a position vector

$$\mathbf{r}=(x_1\cdot\mathbf{x_1}, x_2\cdot\mathbf{x_2}, \ldots, x_n\cdot\mathbf{x_n}).$$

The corresponding total derivatives

$$\frac{d}{de}\tau(\mathbf{r})$$

and

$$\frac{d}{de}\beta(\mathbf{r})$$

are vectors. They are, however, linearly dependent. Therefore, the various ratios between these derivatives that define the four buffering parameters t, b, T, and B will, remarkably, again be dimensionless scalars.

The buffering parameters are largely analogous to the measures of probability.

The four measures induced by the signed probability measure t (t, b, T, B) *(Buffering I - Supplement 7)* share most of their formal properties with the four measures induced by Kolmogorov's probability measure (probabilities vs. odds, and for an event vs. against an event). Similarities and differences are summarized in *Table 2* of *Buffering 1 - Supplement 7*. Note that, for finite sample spaces, Kolmogorov's probability measure p can be obtained as a special case of the signed probability measure t by introducing the additional constraints that the partitioning functions should be discrete (the case covered by *Axiom 3; see Supplement 7*) and be linear functions of the type $y=a\cdot x$ and $z = b\cdot x$, with $a,b \in \mathbb{R}^+ \cup \{0\}$ and $x \in \mathbb{N}_0$. These constraints imply that the number of events is a discrete quantity, and that probabilities are independent of the number of trials.
