# Supplementary material for: The quantitation of buffering action I. A formal & general approach
Source: Theor Biol Med Model. 2005 Mar 15;2:8. doi: 10.1186/1742-4682-2-8 (PMC1079953; doi:10.1186/1742-4682-2-8)
Supplement: Additional File 4 — Constructing Communicating Vessel-Models of Partitioned and Buffered Systems [file 1742-4682-2-8-S4.pdf]

Theoretical Biology and Medical Modelling

Research

The quantitation of buffering action. I. ***A formal and general approach.***

Bernhard M. Schmitt

Supplement 4:

Constructing Communicating Vessel-Models of Partitioned and Buffered Systems

Several examples in the main text have shown how the model of communicating-vessels works in principle *(Figures 1 and 2 in the main text of Buffering I)*. To create such models for other buffered systems that one might encounter, we need a general method or algorithm to construct a corresponding model. The model should of course replicate exactly the quantitative aspect of the original system's partitioning behavior. In addition, it would be advantageous to have the model provide a direct, visual read-out of the buffering strength (in terms of "buffering odds" B), i.e., a sort of "buffering power gauge".

All we need to construct such a model is the quantitative relationship between $y \equiv \tau(x)$ and $z \equiv \beta(x)$. This relationship in implicitely defined in any buffered system and can therefore always be expressed explicitely as a composite function $z(y)=\beta(\tau(x))$. To obtain the model then, we set the dimensions of the transfer vessel T such that its cross-sectional area A_T equals 1 at all fluid levels. For instance, this can be achieved by employing a parallel-walled vessel with a 1×1 square base. Thus, fluid level h equals numerically the fluid volume y inside vessel A. Then, the cross-sectional area $A_B(h)$ of the buffering vessel B at a given fluid level h is:

$$A_B(h) = A_B(y) = \frac{dz}{dy} = \frac{dz/dx}{dy/dx} = b/t = B(h) = B(y)$$

In other words, if the transfer vessel has a unit cross sectional area, the cross-sectional area of the buffering vessel provides a direct visual representation of the buffering ratio B *(Figure 1A)*. Alternatively, we may construct the model in a way that visualizes the transfer ratio T by setting h equal to z, i.e., by setting the cross-sectional area of the buffering vessel to 1 for all fluid levels. Then,

$$A_T(h) = A_T(z) = \frac{dy}{dz} = t/b = T(h) = T(z)$$

(Figure 1B). Finally, the transfer coefficient t and buffering coefficient b by setting h equal to $x=y+z$, i.e., by setting the aggregate cross-sectional area A_{Tot} of both vessels to 1 *(Figure 1C)*.

In general, buffered systems can be modeled by two communicating vessels if they display "simple, non-inverting moderation", i.e., if t never becomes smaller than 0 or greater than 1 within the range to

be modeled. Buffered systems with values of t(x) outside the interval [0,1] cannot be modelled with fixed vessels, because this would require vessels with negative volumes, or with volumes that are decreasing with increasing total volume. However, negative buffering can be modelled using mobile vessels *(cf. Figure 1F in Buffering I - Supplement 6).*

Figure 1: General approach to construct communicating-vessels models of buffered systems.

The distribution of fluid volume inside systems of communicating vessels can replicate and visualize partitioning processes and buffering properties of systems that involve conserved quantities. Non-inverting moderation can be modelled using rigid-walled vessels, whereas non-inverting amplification would require moving parts such as the float lever shown in *Figure 1F* of *Buffering I - Supplement 6*. *Red compartment,* "transfer comparment"; *blue compartment,* "buffering compartment".

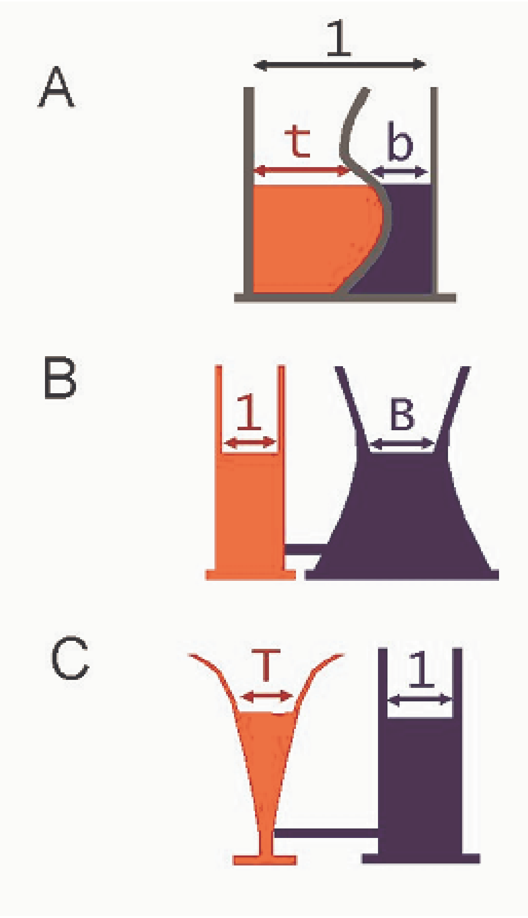

A, Visualizing transfer coefficient t and buffering coefficient b simultaneously.

Cross-sectional areas of transfer and buffering vessel equal t and b, respectively, if their aggregate area is set to 1.

B, Visualizing the buffering ratio B.

Cross-sectional area of buffering vessel equals B if the cross-sectional area of the transfer vessel is set to 1.

C, Visualizing the transfer ratio T.

Cross-sectional area of transfer vessel equals T if that of the buffering vessel is set to 1.
