# Supplementary material for: The quantitation of buffering action I. A formal & general approach
Source: Theor Biol Med Model. 2005 Mar 15;2:8. doi: 10.1186/1742-4682-2-8 (PMC1079953; doi:10.1186/1742-4682-2-8)
Supplement: Additional File 5 — A Trigonometric Representation of Buffering Behavior: The "Buffering Angle" [file 1742-4682-2-8-S5.pdf]

Theoretical Biology and Medical Modelling

Research

The quantitation of buffering action. I. ***A formal and general approach.***

Bernhard M. Schmitt

Supplement 5:

A Trigonometric Representation of Buffering Behavior: The "Buffering Angle"

The buffering parameters can be expressed trigonometrically, i.e., in terms of a "buffering angle" α. This representation has the advantage that it allows one to represent *any* type of buffering behavior by a finite angle (between –45° and 135°), without any discontinuities. In contrast, infinite amplification (inverting or non-inverting) cannot be represented by a finite transfer coefficient t or buffering coefficient b, perfect transfer cannot be represented by a finite transfer ratio T, and perfect buffering cannot be represented by a finite buffering ratio B. Moreover, the buffering angle has a intuitive meaning with respect to the representation of buffered systems by a space curve in R^3 *(see below)*.

The buffering angle can be introduced geometrically: A plot of buffering coefficient b versus transfer coefficient t in a system of rectangular coordinates yields a straight line (because, by definition, t-b=0). Each point on that line may be represented by positional vector $\vec{v} = \begin{pmatrix} t \\ b \end{pmatrix}$. The buffering angle α is the angle between the t-axis and the positional vector $\vec{v}$ *(Figure 1)*.

Figure 1: Buffering Angle

See text of this Supplement for explanation.

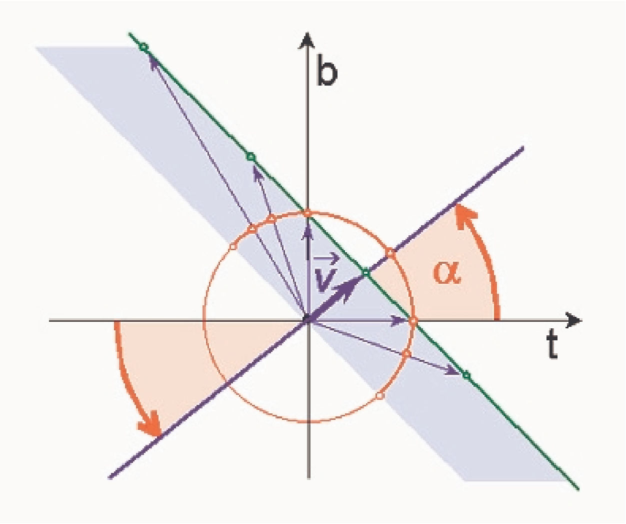

The relation between t and α is a bijection between $\mathbb{R}$ and the interval]-45°,135°[. Specifically, for any transfer coefficient t ($t \in \mathbb{R}$), the unique

corresponding buffering angle α is defined by a mapping of ℝ to the interval]-45°,135°[

$$\alpha : t \rightarrow \arccos\left(\frac{t}{\sqrt{t^2 + b^2}}\right)$$ for t≤1, and

$$\alpha : t \rightarrow -\arccos\left(\frac{t}{\sqrt{t^2 + b^2}}\right)$$ for t>1.

Reversely, for every angle α from the interval]-45°,135°[, the unique corresponding real number t (t∈ℝ) is defined by the mapping of this interval to ℝ:

$$t : \alpha \rightarrow \left(\frac{\cos\alpha}{\cos\alpha + \sin\alpha}\right).$$

Similarly, a given buffering angle unambiguously defines the other buffering parameters:

$$b : \alpha \rightarrow \left(\frac{\sin\alpha}{\cos\alpha + \sin\alpha}\right)$$

$$T : \alpha \rightarrow \left(\frac{\cos\alpha}{\sin\alpha}\right)$$

$$B : \alpha \rightarrow \left(\frac{\sin\alpha}{\cos\alpha}\right)$$

A buffering angle α=0° is equivalent to zero buffering, a buffering angle of 90° to perfect buffering. Further equivalences between transfer coefficient t and buffering angle α are listed in *Table 2 of Buffering 1 – Supplement 7*.

Interestingly, the buffering angle has a direct geometrical meaning with respect to the space curve that represents the buffered system in R^3 *(Figure 3 in the main text of Buffering I)*. The space curve can be projected parallel to the x-axis onto the yz-plane. Then, the buffering angle is the angle enclosed by the tangent to the projected space curve on the one hand, and the y-axis on the other.
