# Supplementary material for: The quantitation of buffering action I. A formal & general approach
Source: Theor Biol Med Model. 2005 Mar 15;2:8. doi: 10.1186/1742-4682-2-8 (PMC1079953; doi:10.1186/1742-4682-2-8)
Supplement: Additional File 6 — From Galton Desk to Communicating Vessels – "Partitioning" as a Common Pattern Behind Probability and Buffering [file 1742-4682-2-8-S6.pdf]

Theoretical Biology and Medical Modelling

Research

The quantitation of buffering action. I. ***A formal and general approach.***

Bernhard M. Schmitt

Supplement 6:

From Galton Desk to Communicating Vessels – "Partitioning" as a Common Pattern Behind Probability and Buffering

"Every axiomatic (abstract) theory admits, as is well known, of an unlimited number of concrete interpretations besides those from which it was derived. Thus we find applications in fields of science which have no relation to the concepts of random event and of probability in the precise meaning of these words." A.N. Kolmogorov [1]

The concept of buffering can be introduced in its most general, mathematical form by means of several axioms; these are presented in *Supplement 7* . The approach taken there for the axiomatic foundation of a measure for buffering action closely resembles in form and content the approach taken by mathematical probability theory for the axiomatic foundation of a measure of „probability", as first proposed by A. Kolmogorov [1-4]. From this slim yet firm foundation rise the theories of mathematical probability theory and the extensive body of rules of applied statistics.

Here, we show that the probabilities observable with a Galton desk can be represented equivalently by a system of communicating vessels, but the latter one exhibits additional properties that cannot be grasped any more by Kolmogorov's axioms. The required modifications result in a set of axioms and a novel measure that expresses a „relative rate of change". Relative rates of change can be used again to quantitate chance, but also to describe buffering action.

A Galton desk as an illustration of Kolmogorov probabilities

Consider the receptacles E_1, E_2, E_3 of the Galton desk shown in *Figure 1A* of this Supplement. Every single one of them can be associated with a "probability" $p(E_i)$ that satisfies the axioms of Kolmogorov's system of probability, or KSP in short [1]. Thus, the probability p is always a nonnegative number between 0 and 1, the probability associated

with the ensemble of all receptacles is unity, and the probability associated with any subset of receptacles equals the sum of the individual probabilities of the receptacles in the subset. Briefly, these axioms of KSP are written:

i) $0 \le p(E) \le 1$

ii) $p(E_1) + p(E_2) + \ldots = 1$

iii) $p(E_1 \cup E_2 \cup \ldots) = \Sigma p(E_i)$.

KSP characterizes and constrains the measure p rigorously on a purely mathematical level, but provides no bridge to the "real world": Not only is it left open how the particular value of $p(E_i)$ can be identified in a given situation. More importantly, the elements E_1, E_2, … and their unions or intersections are entirely abstract, set theoretic entities in KSP, and it is not specified which aspects of the "real world" should be represented by these elements. Usually, these entities are interpreted as "events". This interpretation has been extremely fruitful, yet it is not the only possible one. In fact, neither the term nor concept of an "event" are part of the axioms of KSP.

Kolmogorov probabilities can be interpreted in various ways.

Moreover, the nature of what is quantitated by the measure p and attributed to some element has remained a matter of an ongoing and profound debate among scientists and philosophers. Importantly, KSP itself is compatible with a variety of conflicting interpretations (for detailed discussion and literature, see [2-4].

Firstly, these include the "classical interpretation" of probability which is based on the ratio of the number $|E|$ of cases favorable over the total number $|C|$ of cases which are all assumed to be equally probable:

$$p(E) = \frac{\text{number of favorable cases}}{\text{number of all cases}} = \frac{|E|}{|C|}.$$

The major shortcoming of this concept is its restriction to *a priori* equiprobable events.

Secondly, the "proportional interpretation" of probability defines probability of A as the proportion between a subset A and a set Ω. Herein, the proportion may be computed from the cardinality of the respective sets. When the sets are viewed as geometric objects, proportions can as well be based on some geometric measure S such as area or volume of the respective geometric objects:

$$p(A) = \frac{\text{"size" of subset}}{\text{"size" of set}} = \frac{S(A)}{S(\Omega)}.$$

Such "geometric probabilities" are used, for instance, to treat "Buffons needle problem", or to approximate the transcendent number π by so-called Monte-Carlo Methods.

Finally, the "frequency interpretation" [5] which underlies most of applied statistics views probability as the limit of a relative frequency of a particular event (i.e., the ratio of the number n_i of occurences of a particular event E_i over the total number of events N as the number of trials approaches infinity:

$$p(E_i) = \lim_{N \to \infty} \frac{n_i}{N}.$$

The reference to an "infinite" number of trials implies theoretical and practical limitations of this definition.

Other axiomatic definitions of a probability measure - "Non-Kolmogorov systems"

Importantly, the KSP reflects only partially the original properties of these individual interpretations. With respect to proportionality, for instance, the KSP is unable to replicate the simple case of geometrical proportions that assume values outside the interval [0,1], including real numbers that are greater than 1 or negative *(compare Figure 4 in the main text of Buffering I)*. The limitations follow from one of the KSP axioms which postulates that the probability measure is a nonnegative number.

Problems of this type are inherent to axiomatic theories in general, and it is recognized that alternative axioms exist and are often better suited to treat certain aspects studied by the same discipline. A striking example is geometry: Euclid's axiomatics were equated with geometry in general, until first the logical existence and then the practical power of several "Non-Euclidean" geometries had

to be acknowledged. Similarly, the KSP enjoys a "canonical" status, yet variations of its axiomatics are logically possible, and several such "Non-Kolmogorov" axiomatic systems of probability have been proposed [3,4,6-9]. These considerations can justify on formal grounds our following attempt to gradually introduce several modifications into KSP in order to derive a "Non-Kolmogorov" system of probability. On practical grounds, we justify this attempt by the fact that the resulting system is, unlike KSP, suited to quantitate buffering action.

Figure 1: Modifications of a Galton desk, suggesting modifications of Kolmogorov's concept of probability

(detailed explanation in the text of this Supplement).

A, Classical Galton desk
Spheres dropped from the top have fixed probabilities p_i of ending the i-th box, with $0 \leq p \leq 1$. The number of spheres droppped (i.e., of trials) is a natural number and thus a discrete quantity.

B, Fluid percolating through a system of branching pipes
Probabilities are as in *A*. When fluid volume is thought to be divisible infinitely, the "number of trials" n now is a continuous quantity, with $n \in \mathbb{R}^+$.

C, Fluid welling up a system of branching pipes
Probabilities as in *A* and *B*.

D, Fluid entering a system of communicating vessels
Probabilities as in *A*, *B*, *C*. Regularly shaped communicating vessels can replicate fixed probabilistic behavior.

E, Communicating vessels of irregular shape
With irregularly shaped communicating vessels, the distribution between the compartments depends on the amount of fluid in the system, giving rise to *variable probabilities*.

F, Communicating fluid compartments separated by a mobile float lever
At rising fluid levels, the lever progressively diminishes the volume of the compartment to its right side. Thus, adding fluid to the system paradoxically decreases fluid volume in one compartment, giving rise to *negative probabilities*.

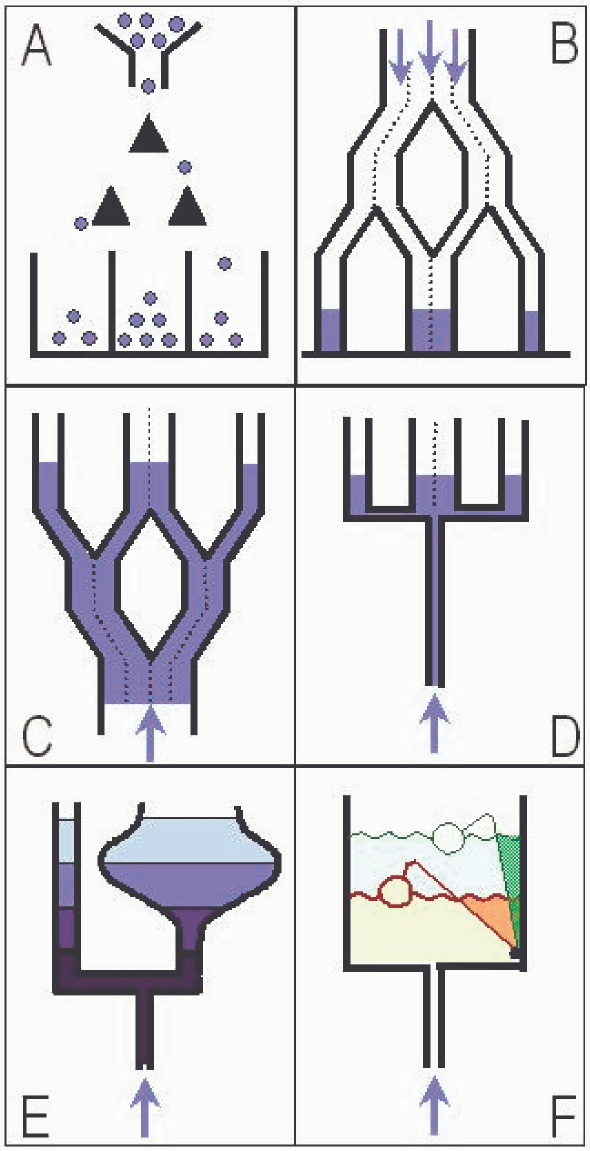

As a conclusion from *Figure 1*, an axiomatic system different from Kolmogorov's is required in order to handle *continuous events* and *variable* or *negative probabilities*. This article presents such an axiomatic system (see *Buffering I - Supplement 7*).

Fluid cascading – from discrete to continuous events

Instead of dropping spheres down a Galton desk, we now pour a fluid down a system of branching pipes. We may continue to think in terms of individual objects (here: individual molecules), and find that the arrangement shown in *Figure 1B* is completely analogous to *Figure 1A* with respect to probabilities. It is simpler and more common however, to express the amount of fluid in terms of a volume rather than in terms of individual molecules. In order to find the probability of the event "added fluid ends up in the i-th vessel", one then accordingly computes the limit of a "relative volume" v_i/V (where v_i and V represent volumes in the i-th vessel and in the entire system, respectively). The probability calculated in this way is of course identical to the probability that is obtained with the limit of the relative frequency n_i/N (where n_i is the number of molecules in the i-th vessel, and N the total number of molecules in the system):

$$\lim_{N\to\infty}\frac{n_i}{N}=\lim_{V\to\infty}\frac{v_i}{V}.$$

There is, however, one momentous difference between these two approaches: Spheres come in numbers that are natural numbers ($n_i,N\in\mathbb{N}$), whereas fluid volume may be any nonnegative real multiple of an appropriate volume unit ($v_i,V\in\mathbb{R}$). Thus, as a first important modification suggested by the fluid model, we define the "number of occurences" of an event as a potentially continuous, infinitely divisible quantity, rather than a discrete, quantitized one (as suggested by the individual spheres).

Fluid welling up – blending probability and proportionality

We now turn the pipework shown in *Figure 1B* upside down and push the fluid up from below *(Figure 1C)*. Surprisingly or not, the proportions according which the fluid distributes between the three vessels do not change. The distribution process is governed again solely by the cross-sectional areas of the individual vessels relative to each other. Because the number of individual molecules is high, the "aleatoric" or "probabilistic" aspect vanishes. This situation is intuitively understandable in terms of a geometric probability: the fluid appears to partition regularly according to the dimensions of the vessels.

Communicating cylindrical vessels – a continuous-event equivalent to Galton's board

Because fluid distribution depends exclusively on the cross-sectional areas of the vessels themselves, but not on the geometry of the pipes that feed the fluid from below, any arbitrarily shaped feeding pipe may be used to represent a given system, e.g. a simple straight one *(Figure 1D)*. With respect to probability, all four systems shown in *Figure 1A-D* are thus equivalent. It follows that the probabilistic properties of a Galton desk can be replicated exactly by an appropriate system of communicating vessels. The communicating vessels-model is more general, however, inasmuch it allows one to model not only discrete "events", but as well processes where the number of "events" is a continuous quantity.

Communicating vessels of irregular shape – inconstant probability can be described by a rate of change.

A derivative yields the local probability for nonlinear, continuous events.

Next, we consider two communicating vessels one of which is irregularly shaped *(Figure 1E)*. The cross-sectional area of one vessel relative to the other (or relative to the whole) will now vary according to the fluid level. By the same token, the probability of a single molecule to enter a particular vessel (or the fraction of added fluid that does so) will depend on the fluid level. Then, the proportions between *absolute* quantities do not any longer reflect correctly these probabilities or proportions, neither the absolute frequencies (n_i and N) used to compute relative frequencies, nor the absolute volumes (v_i and V) used to compute relative volumes. In the case of a vessel that is tapering off towards its upper end, for instance, the estimates obtained by these approaches will be too large, and in the case of a widening vessel, they will be too small. More

irregular vessel shapes will result in under- or overestimation of the true probability in a complicated way.

In contrast to the ratios of absolute frequencies or volumes (i.e., n_i/N or v_i/V), the corresponding differential dv_i/dV yields the correct probability at any fluid level:

$$p(E_i, V) = \left(\frac{dv_i}{dV}\right).$$

This differential is here equal to the ratio a_i/A of individual to total cross-sectional area of the vessels at the given fluid level. For systems of regularly shaped vessels with constant cross-sectional areas (e.g. cylinders), the ratio a_i/A is constant and equal to the ratio v_i/V of the absolute volumes. In all other cases, these ratios vary, and probability becomes a function of two variables: the probability $p(E_i,V)$ depends on both the particular event E_i (here: fluid entering the i-th vessel) and the total quantity of events (here represented by total volume V). Similar to these proportions or probabilities, numerous physical quantities are defined most efficiently as differentials.

A slope between two points of a function yields the probability for discrete processes.

If we wish to compute the probabilities in this situation in terms of individual spheres or molecules, we notice that the corresponding functions are discrete rather than continuous. Therefore, the corresponding differentials do not exist. On the other hand, there certainly exists a specific probability with which the corresponding events will occur. For continuous functions, we could compute a rate of change for a single point, namely as the derivative of that function. For discrete functions, a rate of change can only be computed for a pair of different points of the function. Such a rate of change can be used to compute probabilities for discrete processes.

In a plot of absolute frequency n_i of molecules in the i-th vessel against the number N of molecules in the system, the rate of change between two consecutive points N, $N+1$ equals the slope of the line that connects these points:

$$p^*(E_i, N, N+1) = \left(\frac{n_i(N+1) - n_i(N)}{1}\right).$$

For a single "experiment" in which the system is successively filled with fluid molecules, the value of p* will be either 0 or 1.

Alternatively, one may perform a greater number k of such experiments, and then plot the average number $\hat{n}_i(N)$ of molecules in the i-th vessel against N. Then, the slope of the line through two consecutive points can assume any nonnegative real value. Moreover, if one then lets k approach infinity, the slope becomes equal to the probability that a molecule added on top of N molecules already present in the system will go into the i-th vessel:

$$p(E_i, N, N+1) = \lim_{k\to\infty}\left(\frac{\hat{n}_i(N+1) - \hat{n}_i(N)}{1}\right)$$

where

$$\hat{n}_i = \frac{1}{k}\sum_{j=1}^{k} n_{ij}\ .$$

More generally, we can consider not just consecutive points N, $N+1$ of the discrete function $N \to n_i$, but any two points that are apart by any distance $\Delta N \in \mathbb{Z}$, and compute a probability p^* of an event for that specific interval, either for a single experiment:

$$p^*(E_i, N, N+\Delta N) = \left(\frac{\Delta n_i}{\Delta N}\right) = \left(\frac{n_i(N+\Delta N) - n_i(N)}{\Delta N}\right),$$

or for the average from multiple experiments:

$$\begin{aligned} p(E_i, N, N+\Delta N) &= \lim_{k\to\infty}\left(\frac{\Delta \hat{n}_i}{\Delta N}\right) \\ &= \lim_{k\to\infty}\left(\frac{\hat{n}_i(N+\Delta N) - \hat{n}_i(N)}{\Delta N}\right). \end{aligned}$$

The slope $\Delta\hat{n}_i/\Delta N$ may be interpreted as a probability of the i-th event for the interval $[N, N+\Delta N]$, or as the average fraction of molecules that go into the i-th vessel if a number ΔN of

molecules are added to N molecules already present in the system.

Probabilities of an event as nonconstant functions

Note that we cannot simply use the frequentist approach to probability and compute probability by letting N approach infinity, because we allowed n_i to be a nonlinear function of N, corresponding to variable probabilities. Variable probabilities may also be observed in classical probabilistic experiments, e.g. when rolling dice. Ideal dice, on the one hand, are endowed with probabilities that are not only equal for every possible event {1,2,3,4,5,6}, but that are also constant no matter how many times the dice are rolled. For real dice, on the other hand, the different outcomes are almost certainly not exactly equiprobable. Furthermore, they are subjected to uneven wear and tear, or parts may break off. As a consequence, real dice exhibit probabilities that are drifting or jumping as a function of how many times it is rolled. The measures $p^*(E_i, N, N+\Delta N)$ and $p(E_i, N, N+\Delta N)$ can be applied to the analysis of dice with such drifting or jumping probabilities.

In sum, we retain from these examples the notion that the probability associated with a given element of the event space need not be constant, but may vary as a strict function of the number of events.

Communicating vessels with mobile separating wall - negative probabilities

Positive total flow into a system can be associated with a negative flow into an individual compartment.

Systems of communicating vessels may be constructed in such a way that the volume inside a particular vessel does not increase monotonically with increasing total volume in the system, but can actually decrease. For instance, the wall separating two communicating compartments can be built to form a mobile float lever *(Figure 1F)*. When adding fluid to the empty system, the compartment to the right will then fill initially. As the fluid level rises, the separating wall will increasingly compress that compartment and ultimately displace the fluid inside entirely. Volume will thus flow *out* of the right compartment, reflected in a negative sign of the differential dv_i/dV (or in a negative slope $\Delta n_i/\Delta N$). Moreover, flow into the left compartment will be greater than net flow into the entire system.

"Signed measures"

In mathematical measure theory, a measure whose range comprises both negative and nonnegative real numbers is termed a "signed measure". The underlying concept is simple and ubiquitous in science and everyday life. A familiar example of a signed measure is electric charge: An object such as a membrane protein may carry various negative or positive charges distributed over its volume, such that each volume element is associated with a negative or positive measure of charge. These partial charges may exceed the net charge of the entire protein. Other examples include the buoyancy of a composite body (e.g. a submarine or a ballon), and various types of partial flows that add up to a net flow (charge flow across a cell's membrane, daily expenses and revenues of a company, emigration from and immigration into a country, etc.).

Negative rates of change can be interpreted as negative probabilities.

In the previous examples which involved nonnegative quantities, we saw that the differential dv_i/dV reflects the proportion of partial to total volume flow. The numerical value of this proportion represents, however, also the correct value of the associated probability that addition of a fluid molecule to the system will increase the total number of fluid molecules in a particular compartment. Moreover, when the differential dv_i/dV becomes negative, one can well continue to interpret it as a "probability". Not only is such a usage of the term free of contradictions, but it also makes sense intuitively: Probabilities associated with positive flow are perceived to be greater than those with zero flow, and probabilities associated with zero flow to be greater than probabilities associated with negative flow. "Negative probabilities" are unfamiliar because they do not exist in KSP. In fact, however, they allow for a more natural and intuitive numerical representation of probability than probability measures that –by act of postulate- uniformly assign "zero probability" to any flow that is equal to or smaller than zero flow.

Formally, negative probabilities can be accomodated simply by omitting the corresponding postulate of KSP that probability should be a nonnegative quantity. The resulting signed measure differs in several respects from the nonnegative probabilities according to KSP, but is consistent internally *(see Supplement 7)*.

Several compartments with similar properties – from sets to "multisets" or "bags"

Kolmogorov's system of probability postulates that the event space be a set, i.e. a collection of strictly different elements. When rolling a die, for instance, the event space is a set {1,2,3,4,5,6} of six different numbers. Assuming that all outcomes are equiprobable, KSP allows us to compute the probability of the outcome "1" as p=1/6. Now, assume that we change the labeling of the six faces of the die in such a way that some numbers occur more than once, e.g. using the number 2 twice, and the number three thrice. This changes the event space to [1, 2, 2, 3, 3, 3].

On the one hand, we correctly feel that there must again exist such a thing as the probability of the outcome "1", and we would not hesitate to say that is 1/6 as before. Clearly, however, this event space is not a set, but a "multiset" or "bag", i.e., a collections of not necessarily different elements. Consequently, a set theoretic approach such as KSP cannot be applied to this system. In order to derive the probability of the outcome "1" within the framework of KSP, one might transform the event space into a classical set by introducing more or less construed distinctions between the otherwise identical outcomes "2" or "2" and "3" or "3" or "3".

More often, set theory seems to be traded tacitly for other approaches such as the "classical" interpretation of probability. The classical interpretion readily handles bag-type event spaces because it only requires that the outcomes be equiprobable, but not necessarily distinguishable or different in the sense of a set. This is one of many examples showing that KSP integrates only some, but not all aspects of probability [3,4].

Bags are ubiquitous and natural data structures.

Event spaces that constitute bags but not sets are not merely a curiosity, but actually rather frequent, and our mind frequently structures collections of objects automatically in bag-type fashion. A literal shopping bag containing two apples and five potatoes provides a simple example for a collection of elements that is a bag and not a set. Multiple copies of identical elements are found in libraries and stamp collections. The body comprises numerous cells (~1-5×10^{12}), but of relatively few different types (~250). A given organ such as blood contains billions of individual cells, but a blood count represents them clearly as a bag (i.e., type of cell and number of copies per microliter of blood) and not as a set. Similarly, bags readily represent the organelles of a single cell *(nucleus×n_1, mitochondria×n_2, lysosome×n_3, etc.)*, the organs of an organism *(heart×1, lung×2, parathyroid×4, etc.)*, the entire biochemical warehouse of a cell, or the very compounds that act as H^+ buffers *(bicarbonate×n_1, histidine×n_2, phosphate×n_3, etc.)*, and so on. Many examples are also found in this and the accompanying article. Formally, bag data types are more general than sets, and include genuine sets as a special case. Taken together, a final modification of the original KSP shall accommodate event spaces that are bags rather than sets.

Required modifications to KSP

As shown above, the differential dv_i/dV provides a signed measure that can be used to express the proportion between partial and total rates of change, and "probability" is one possible and useful interpretation of that measure. Due to some modifications, however, this measure is not covered any longer by the KSP axioms. The modifications became necessary to accommodate

i) events as a continuous quantity,

ii) probabilities that vary as a function of the number of events,

iii) negative probabilities, and

iv) measurable spaces of the bag type. By means of several "Non-Kolmogorov" axioms, we can put this measure on its own firm ground.

References

1. AN Kolmogorov: *Grundbegriffe der Wahrscheinlichkeitsrechnung*. Berlin: Springer; 1933.
2. H Primas: **Basic elements and problems of probability theory.** *J Scientific Exploration* 1999, **13:** 579-613.
3. AY Khrennikov: **Interpretations of probability and their p-adic extensions.** *Theory Probab Appl* 1998, **46:** 256-273.
4. AY Khrennikov: *Interpretations of probability*. VSP, Zeist, Netherlands: 1999.
5. Rv Mises: **Grundlagen der Wahrscheinlichkeitsrechnung.** *Mathematische Zeitschrift* 1919, **5:** 52-99.
6. K Svozil. Some remarks on generalized probabilities. 2003. http://tph.tuwien.ac.at/~svozil/publ/2001-cesena.htm
7. A Renyi: **A new axiomatic theory of probability.** *Acta Mathematica Academia Scientiarum Hungaricae* 1955, **6:** 85-335.
8. RP Feynman: **Negative probability.** In *Quantum implications. Essays in honour of David Bohm*. Edited by Hiley BJ, Peat FD. London: Routledge and Kegan Paul; 1987:235-248.
9. PAM Dirac: **The physical interpretation of quantum mechanics.** *Proc Roy Soc London Ser A* 1942, **180:** 1-39.
