# Supplementary material for: The quantitation of buffering action I. A formal & general approach
Source: Theor Biol Med Model. 2005 Mar 15;2:8. doi: 10.1186/1742-4682-2-8 (PMC1079953; doi:10.1186/1742-4682-2-8)
Supplement: Additional File 7 — Axiomatic Foundation of the Formal & General Approach [file 1742-4682-2-8-S7.pdf]

Theoretical Biology and Medical Modelling

Research

The quantitation of buffering action. I. *A formal and general approach.*

Bernhard M. Schmitt

Supplement 7:

Axiomatic Foundation of the Formal and General Approach

"The theory of probability, as a mathematical discipline, can and should be developed from axioms in exactly the same way as Geometry and Algebra. (…) The postulational basis of the theory of probability can be established by different methods in respect to the selection of axioms as well as in the selection of basic concepts and relations."

A.N. Kolmogorov [1]

Overview

In the *Supplement 6*, we saw that the probabilities observable with a Galton desk can be represented equivalently by a system of communicating vessels, but the latter one exhibits additional properties that cannot be grasped any more by Kolmogorov's axioms. The required modifications will result in a different set of axioms and a novel measure that expresses a „relative rate of change". Relative rates of change can be used again to quantitate chance, but also to describe buffering action. The axioms can therefore be considered the general form of the concept of buffering.

Here, we present this system of axioms. The axioms define a „signed measure" on a particular „measurable space". The approach taken is a variation on Kolmogorov's axiomatic definition of a probability measure [1], and can be considered a generalization thereof [2-4].

Preliminaries: bags or multisets

A bag (or multiset) is a collection of elements that may contain duplicates *(Figure 1)*. Bag algebraic definitions and notation used here largely follow the system proposed by Albert [5]. We can notate

a bag A that contains a finite number of elements as a list of its elements between brackets, such as A=[a,a,b,b,b,c], or by listing the different elements, preceded by a counter, such as A=[2a, 3b,1c]. In general, we can notate a finite bag A as $A=[n_i,s_i]$, where s_i denotes the i-th element of A and n_i denotes the number of copies (or multiplicity) of s_i in A. As this notation suggests, one may define a bag as a mapping A from a fixed set S of elements s_i on the set of the natural numbers including zero: $A: S \rightarrow \mathbb{N}_0$. The set S is also termed the "universe". In this view, sets form a particular class of bags, namely of those bags that do not contain any duplicates, i.e., of those bags $A=[n_i,s_i]$ for which $n_i \in [0,1]$.

Figure 1: Bags & Partitioned Systems

A classical ***set*** contains only different elements. In contrast, a ***multiset*** or ***bag*** may contain multiple copies of each element. A ***partitioned system*** over a bag M consists of several ***subbags*** whose concatenation equals M. Accordingly, a ***two-partitioned system*** consists of exactly two such subbags or ***partitions***. Among others, there are ***bags of functions***, e.g. of functions of a common independent variable x. One may construct two-partitioned systems of functions over a given bag of functions. By definition, a partition containing several individual functions is equivalent to a partition containing a single ***partitioning function* π(x)** whose value equals the sum of the values of all individual functions. ***Partitioned systems of functions*** are suited to represent mathematically in a simple and direct way systems and processes in which a single global variable determines the state of the various individual parts. For instance, total fluid volume in a system of communicating vessels determines the individual volumes inside each vessel (*cf. Figures 2 in the main text of Buffering I*).

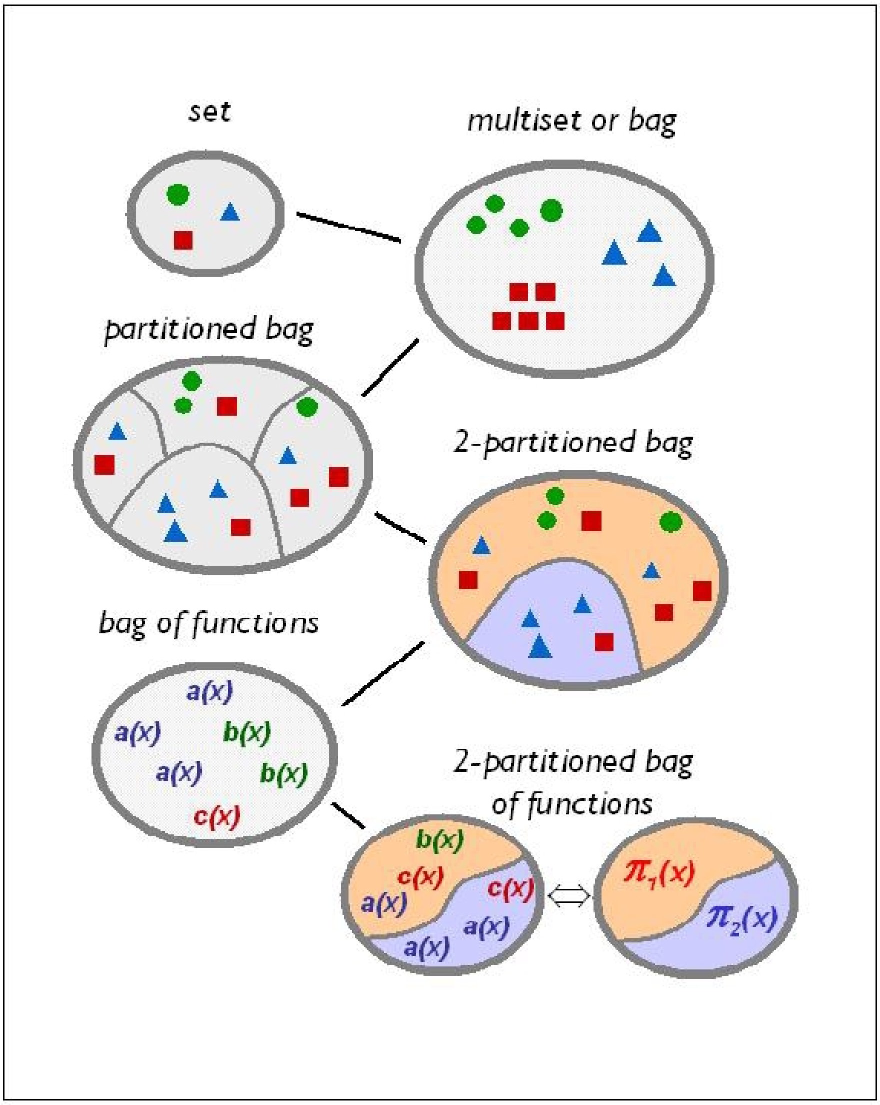

Similarly, there are bag-type "event spaces" in probability theory; such event spaces cannot be handled by set-theoretical axiomatic systems of probability.

If we represent symbolically the operation that returns the number n_i of copies of an element s_i from a bag A as $(s_i \in\in A)=n_i$, we can write down the definitions of some basic bag operations as follows:

Bag containment:

$A \subseteq B \equiv s \in A \Rightarrow (s \in\in A) \leq (s \in\in B)$

Bag concatenation:

$A \sqcup B \equiv$ the bag C such that $s \in S$

$\Rightarrow s \in\in C = (s \in\in A) + (s \in\in B)$

Bag union ("minimal union"):

$A \cup B \equiv$ the smallest bag C such that

$A \subseteq C$ and $B \subseteq C$

Bag difference:

$A \backslash B \equiv$ the bag $C \subseteq A$ such that

$s \in S \Rightarrow s \in\in C = \max(s \in\in A - s \in\in B, 0)$

Bag intersection:

$A \cap B \equiv$ the largest bag such that $C \subseteq A$ and $C \subseteq B$.

The operations indicated by the symbols "$\subseteq, \cup, \backslash, \cap$" are natural extensions of the respective set theoretic operations to bags. In other words, when these operations are applied to sets, they have their usual meaning. Bag concatenation $\sqcup$ has no set theoretic origin; when applied to sets with no elements in common, bag concatenation becomes equivalent to set union $\cup$.

Partitioned systems, partitions and partitioning functions of a bag, sigma function

A collection of subbags S_i from a universe F whose concatenation equals F is called a "partitioned system" Π of a bag F:

$$\text{"partitioned system } \Pi \text{ of } F\text{"} = \{\Pi = [n_i, S_i] \mid \coprod_{S \in \Pi} S = F\}.$$

The subbags $S_i \in \Pi$ are then termed "partitions" of F *(Figure 1)*. Among the "multi-partitioned" or "n-partitioned" systems, the "two-partitioned" systems will be of particular relevance for the quantitation of buffering action. Partitioned systems in bag theory are analogous to partitioned systems or "decompositions" in set theory. However, in contrast to the partitions of a set, the partitions of a bag are not necessarily "disjoint", i.e., their pairwise intersections are usually nonempty. If the bag F is a set, the bag theoretical definition of a partitioned system becomes equivalent to the standard set theoretical definition.

The elements in the partitions of a bag may be functions f_i of a single common variable x. We can then define as "partitioning function" π_S of a partition S that function whose value equals the sum of all individual functions in S *(Figure 1)*:

$$\pi_S : x \rightarrow \sum_{f \in S} f(x)$$

If the elements of S are bags of functions rather than individual functions, the partitioning function of S is the function whose value equals the sum of the functions in the bag that is obtained by the concatenation of these bags of functions. Higher degrees of nesting require concatenations on every level of nesting in order to generate a "flat" bag of individual functions f_i.

Furthermore, we term the partitioning function of the universe F the "sigma function" $\sigma(x)$:

$$\sigma : x \rightarrow \sum_{f \in F} f(x) = \pi_F(x).$$

Definition of a signed probability space: *Sample space, σ-algebra, signed measure*

A triple (M,**P**,t) is called a "signed probability space" if it satisfies the following conditions:

1. Sample space

Let F be a finite bag of functions f_i of a single variable x from a domain $\mathbb{D}$:

$F = [n_i, f_i]; \quad n \in N_0,\ f_i: x \rightarrow f_i(x),\ x \in \mathbb{D},\ f_i(x) \in \mathbb{R}$.

Furthermore, let x_1 and x_2 be elements of the domain $\mathbb{D}$. Then, the sample space M is given by the cross product of F and the two x values:

$M = F \times x_1 \times x_2$.

Thus, the sample space M is a finite bag of ordered triples $m_i = (f_i, x_1, x_2)$. The triples will serve to study the average changes of the individual functions f_i over a particular interval $[x_1, x_2]$, given as the ratio $\Delta y/\Delta x$ with $\Delta x = x_2 - x_1$ and $\Delta y = f_i(x_2) - f_i(x_1)$. In the limiting case, the interval $[x_1, x_2]$ may become infinitely small; then, the average rate of change approaches the derivative $df_i(x)/dx$ at that point.

2. σ-algebra

Let A denote a subbag of M: $A \subseteq M$. In other words, the bag A is a collection of not necessarily different ordered triples $m_i=(f_i, x_1, x_2)$ out of the sample space M thus that A is contained in M.

The "power set" of M, denoted **P**(M), is the set that contains all different subbags A of M:

$$\mathbf{P}(M) = \{A \mid A \subseteq M\} = \{S \times x_1 \times x_2 \mid S \times x_1 \times x_2 \subseteq M\}$$

Because the bag M is finite, the power set **P**(M) is finite. Analogous to σ-algebras over genuine sets, the finite power set **P**(M) over a bag M constitutes what we may call a "bag σ-algebra in M" inasmuch it has the following properties:

a) The empty bag is in **P**(M):

$$\emptyset = \emptyset \times x_1 \times x_2 \in \mathbf{P}(M)$$

b) If A is in **P**(M), the difference M\A (or complement of A in M) also belongs to **P**(M):

$$A = S \times x_1 \times x_2 \in \mathbf{P}(M) \Rightarrow M \backslash A = (F \backslash S) \times x_1 \times x_2 \in \mathbf{P}(M)$$

c) If the bags A_1 and A_2 are in **P**(M), their intersection is in **P**(M):

$$A = S_1 \times x_1 \times x_2 \in \mathbf{P}(M) \wedge A_2 = S_2 \times x_1 \times x_2 \in \mathbf{P}(M)$$
$$\Rightarrow A_1 \cap A_2 \in \mathbf{P}(M)$$

d) For every collection of elements of **P**(M), their minimal union belongs to **P**(M):

$$J = [n_i, A_i] = [n_i, S_i \times x_1 \times x_2] \wedge A_i = S_i \times x_1 \times x_2 \in \mathbf{P}(M)$$
$$\Rightarrow \left(\bigcup_{A \in J} A \right) \in \mathbf{P}(M) .$$

In principle, other "bag σ-algebras" can be built over M. However, all of them are contained in the power set **P**(M) which can thus serve as a universal bag σ-algebra. The bag M together with the bag σ-algebra **P**(M) constitutes a "measurable space" (M, **P**(M)). Note that *not* all properties of a "classical σ-algebra" (i.e., one that is derived from a set–type sample space) will hold in a "bag σ-algebra" (i.e., one that is derived from a bag–type sample space), except for the trivial case that the bag M is a set.

3. Signed Measure

Axiom 1: The measure t is a mapping of **P**(M) on the set of the real numbers:

$$t: \mathbf{P}(M) \rightarrow R .$$

Thus, the function t assigns a real number r to every subbag $A \in \mathbf{P}(M)$. The following notations of t are considered equivalent:

$$t(A) = t(S \times x_1 \times x_2) = t(S, x_1, x_2)$$

Axiom 2: If A is the empty bag, the measure t of A has a value of zero:

$$A = \emptyset \times x_1 \times x_2 = \emptyset \Rightarrow t(A) = t(\emptyset, x_1, x_2) = 0.$$

Axiom 3: For any nonempty bag $A = S \times x_1 \times x_2 \in \mathbf{P}(M)$ for which $x_1 \neq x_2$ (i.e., $\Delta x \neq 0$) and $A, S \neq \emptyset$, the value of t(A) is equal to the difference between the values of the partitioning function of A at x_1 and x_2, divided by the difference between the corresponding values of the sigma function:

$$A, S \neq \emptyset \wedge x_1 \neq x_2 \;\; \Rightarrow \;\; t(A) = t(S, x_1, x_2)$$
$$= \left(\frac{\Delta \pi_S}{\Delta \sigma} \right) = \left(\frac{\pi_S(x_2) - \pi_S(x_1)}{\sigma(x_2) - \sigma(x_1)} \right) = \left(\frac{\sum_{f \in S} (f(x_2) - f(x_1))}{\sum_{f \in M} (f(x_2) - f(x_1))} \right).$$

In words, the measure t expresses the change (or rate of change) of π_S within an interval $[x_1, x_2]$ normalized with respect to change (or rate of change) of σ. This corresponds to the proportion between a part and a whole, specifically: between a partial change and a total change, or to a "relative change" (or "relative rate of change"). This axiom is applicable regardless of whether the functions in F are discrete or continuous.

Axiom 4: If the functions f_i in F are differentiable and $x_1 = x_2 \equiv x$ (i.e., $\Delta x = 0$), then the value t(A) of a nonempty bag $A \in \mathbf{P}(M)$ or triple (S, x_1, x_2) is equal to the first derivative of the partitioning function of S, divided by the first derivative of the sigma function:

$$A, S \neq \emptyset \wedge (x_1 = x_2) \wedge \lim{}^{+}_{\Delta x \to 0} \left(\frac{\Delta \pi_S}{\Delta \sigma} \right) = \lim{}^{-}_{\Delta x \to 0} \left(\frac{\Delta \pi_S}{\Delta \sigma} \right)]$$
$$\Rightarrow t(A) = t(S, x_1, x_2) = \lim_{\Delta x \to 0} t(S, x_1, x + \Delta x)$$

$$= \lim_{\Delta x \to 0} \left(\frac{\Delta \pi_S}{\Delta \sigma} \right) = \left(\frac{d\pi_S(x)}{d\sigma(x)} \right) = \frac{\left(\frac{d\pi_S(x)}{dx} \right)}{\left(\frac{d\sigma(x)}{dx} \right)} = \frac{\pi_S'(x)}{\sigma'(x)} .$$

In this special case of $x_1 = x_2$, the measure t expresses again the "relative change" (or "relative rate of change") of π_S relative to σ, but for a single point rather than for a finite interval. When the values of

x_1 and x_2 are identical, we can simply write $t(S,x)$ as an equivalent to $t(S,x_1,x_2)$.

The map t: $\mathbf{P}(M) \rightarrow \mathbb{R}$ constitutes a signed measure within the measurable space $(M,\mathbf{P}(M))$. The triplet $(M,\mathbf{P}(M),t)$ is a measure space that can be viewed as a "Non-Kolmogorov probability space".

Corollaries to axioms 1-4:

a) Normalization: The measure associated with the universe $M \in \mathbf{P}(M)$ equals unity, irrespective of the values of x_1 and x_2:

$$t(M)=t(F,x_1,x_2)=\left(\frac{\Delta\sigma(x)}{\Delta\sigma(x)}\right)=1.$$

b) σ-additivity: For any pair (x_1,x_2), the measure of the entire sample space equals the sum of the measures of its individual partitions:

$$t(\Pi,x_1,x_2)=\sum_{S\in\Pi} t(S,x_1,x_2)\,.$$

This statement holds not only for Π, but also for subbags of Π.

c) Domain: The map t is not defined if the denominator is zero in Axiom 3 $[\Delta x \neq 0 \wedge \Delta\sigma = 0]$ or in Axiom 4 $[\Delta x = 0 \wedge \sigma'(x) = 0]$.

Table 1 compares this signed probability measure (or SPM in brief) with Kolmogorov's system of probability (KSP).

Table 1: Comparison between Kolmogorov probabilities and the signed probability measure t

KSP, *Kolmogorov's axiomatic system of probability;* SPM, *the signed probability measure t as defined by the above axioms.*

	**KSP**	**SPM**
universe:	a set Ω	a finite multiset/bag M
Elements of universe:	undefined<br>*(e.g. "elementary events")*	triples (S,x_1,x_2)<br>*(where S is a collection of functions)*
elements of σ-algebra:	subsets of the universe Ω<br>*(e.g. "events" E)*	subbags of the universe M
range of measure:	$p\in[0,1]$<br>*(an axiom)*	$t\in\mathbb{R}$<br>*(negative probabilities possible)*<br>*(an axiom)*
normalization:	$p(\Omega)=1$<br>*(an axiom)*	$t(M)=1$<br>*(a corollary)*
σ-additivity:	for elements of a partitioned system<br>*(i.e., mutually exclusive sets)*<br>*(an axiom)*	for elements of a partitioned system<br>*(i.e., bags with possible identical elements)*<br>*(a corollary)*
Quantity of occurrences of an event	a discrete variable<br>$(n\in\mathbb{N})$	a continuous variable<br>$(x\in\mathbb{R})$

Measures that are useful for the quantitation of buffering action

The signed probability measure t describes the relative rate of change of partitioning function π_S within an interval $[x_1,x_2]$. We can define a "buffered system" as the ordered combination of the partitioning function π_S of S and the partitioning function $\pi_{F\backslash S}$ of the complement F\S of S. For notational simplicity and clarity, we can write the ordered combination (π_S, $\pi_{F\backslash S}$) equivalently as (τ, β), where τ is called the "transfer function", and β is called the "buffering function". Here, we define in a purely formal way several measures that serve to describe the behavior of buffered systems. Buffered systems can be interpreted fruitfully as formal representations of "buffering phenomena" in the common sense of the term, and the measures serve to quantitate buffering action. These specific interpretations are discussed in detail in the main text.

Definition of buffering parameters as functions of a partition A

We define the following "buffering parameters" (see main text for further explanation):

"Transfer coefficient t":

$$t(A) \equiv t(A)$$

"Buffering coefficient b":

$$b(A) \equiv t(M\backslash A)$$

"Transfer ratio T" or "transfer odds T":

$$T(A) \equiv t(A)/b(A)$$

"Buffering ratio B" or "buffering odds B":

$$B(A) \equiv b(A)/t(A)$$

In this notation, the buffering parameters are expressed as functions of A, e.g. $t: A \rightarrow t(A)$. In other words, the subbag S of F in the expression $A=S\times x_1\times x_2$ is allowed to vary, and the pair (x_1,x_2) is assumed to be constant.

Definition of buffering parameters as functions of x

Alternatively, we may fix the subbag S and furthermore limit ourselves to the special case of $x_1=x_2\equiv x$ *(see Axiom 4)*; the buffering parameters can then be expressed as functions of a single variable x, e.g. $t: x \rightarrow t(x)$. We obtain the following equivalences:

Transfer coefficient:

$$t(x) \equiv t(S,x)$$

Buffering coefficient:

$$b(x) \equiv t(F\backslash S,x)$$

Transfer ratio:

$$T(x) \equiv \frac{t(x)}{b(x)} = \frac{t(S,x)}{t(F\backslash S,x)}$$

Buffering ratio:

$$B(x) \equiv \frac{b(x)}{t(x)} = \frac{t(F\backslash S,x)}{t(S,x)}$$

Trigonometric equivalent of buffering parameters: "buffering angle"

The buffering parameters can furthermore be expressed trigonometrically, i.e., in terms of a "buffering angle" α. The buffering angle can be introduced geometrically: A plot of buffering coefficient b versus transfer coefficient t in a system of rectangular coordinates yields a straight line (because, by definition, t-b=0). Each point on that line may be represented by positional vector $\vec{v} = \begin{pmatrix} t \\ b \end{pmatrix}$. The buffering angle α is the angle between the t-axis and the positional vector $\vec{v}$ (*Figure 4 in Buffering I - main text, and Buffering I – Supplement 5*).

The relation between t and α is a bijection between $\mathbb{R}$ and the interval]-45°,135°[. Specifically, for any transfer coefficient t ($t\in\mathbb{R}$), the unique corresponding buffering angle α is defined by a mapping of $\mathbb{R}$ to the interval]-45°,135°[

$$\alpha : t \rightarrow \arccos\left(\frac{t}{\sqrt{t^2+b^2}}\right) \qquad \text{for } t\leq 1,$$

and

$$\alpha : t \rightarrow -\arccos\left(\frac{t}{\sqrt{t^2+b^2}}\right) \qquad \text{for } t>1.$$

Reversely, for every angle α from the interval]-45°,135°[, the unique corresponding real number t ($t\in\mathbb{R}$) is defined by a mapping of this interval to $\mathbb{R}$:

$$t : \alpha \to \left(\frac{\cos\alpha}{\cos\alpha + \sin\alpha} \right).$$

The buffering angle has a direct geometrical meaning with respect to the space curve that represents the buffered system in R^3 (*Figure 3D* in the main text of *Buffering I*). The space curve can be projected onto the **yz**-plane parallel to the **x**-axis. Then, the buffering angle is the angle enclosed by the tangent to the projected space curve on the one hand, and the **y**-axis on the other.

Equivalences between transfer coefficient **t** and buffering angle α are listed in *Table 2*.

Table 2: Equivalences between transfer coefficient t and buffering angle α.

t	α	Buffering characteristics
1	0°	zero buffering
0 to 1	90° to 0°	non-inverting moderation
0	90°	perfect buffering
-1 to 0	116.57° to 90°	inverting moderation
-1	116.57°	perfect inversion
-∞ to –1	135° to 116.57°	inverting amplification
-∞	135°	infinite amplification, inverting
1 to +∞	0° to –45°	non-inverting amplification
+∞	-45°	infinite amplification, non-inverting

References

1. **AN Kolmogorov: *Grundbegriffe der Wahrscheinlichkeitsrechnung*. Berlin: Springer; 1933.**
2. H Primas: **Basic elements and problems of probability theory.** *J Scientific Exploration* 1999, **13:** 579-613.
3. AY Khrennikov: **Interpretations of probability and their p-adic extensions.** *Theory Probab Appl* 1998, **46:** 256-273.
4. AY Khrennikov: *Interpretations of probability*. VSP, Zeist, Netherlands: 1999.
5. J Albert: **Algebraic properties of bag data types.** *Proceedings of the 17th International Conference on Very Large Data Bases, September 3-6, 1991, Barcelona,*Eds. GM Lohman, A Sernadas, and R Camps, Morgan Kaufmann 1991. http://www.sigmod.org/vldb/conf/1991/P211.PDF or http://www.vldb.org/conf/1991/P211.PDF
6. A Roos, WF Boron: **The buffer value of weak acids and bases: origin of the concept, and first mathematical derivation and application to physico-chemical systems. The work of M. Koppel and K. Spiro (1914).** *Respir Physiol* 1980, **40:** 1-32.
