# Supplementary material for: The quantitation of buffering action I. A formal & general approach
Source: Theor Biol Med Model. 2005 Mar 15;2:8. doi: 10.1186/1742-4682-2-8 (PMC1079953; doi:10.1186/1742-4682-2-8)
Supplement: Additional File 8 — Conservative and Non-Conservative Partitioned Systems – Equivalences and Interconversions [file 1742-4682-2-8-S8.pdf]

Theoretical Biology and Medical Modelling

Research

The quantitation of buffering action. I. ***A formal and general approach.***

Bernhard M. Schmitt

Supplement 8:

Conservative and non-conservative partitioned systems: equivalences and interconversions

The axioms define the basic data structure in which buffering terminology makes sense.

Apparently, the buffering concept and the associated terminology are applicable if and only if the system under study has been structured in a particular way. Namely, the elements of a whole must be grouped into exactly two separate groups both of which can be condensed into a single function of a common variable. In the axiomatic definition of the signed probability measure t and the other three buffering parameters b, T, B *(Supplement 7)*, this grouping is reflected in an ordered system of a subbag and its unique complement.

For instance, if F is a bag of functions f_i, then choosing a particular subbag S out of F induces a complement S^C. Together, S and S^C define a unique ordered two-partitioned system {S, S^C}, where the two partitions S and S^C each possess unique partitioning functions $\tau(x)$ and $\beta(x)$, respectively. The ordered pair of functions can be represented as a buffered system $\{\tau(x), \beta(x)\}$.

Analogously, if M is a bag of triples (S,x_1,x_2), where S is a subbag from F, and x_1,x_2 are two elements of the common domain of the functions f_i, then a particular subbag A out of M induces, together with its complement A^c, a buffered system $\{A,A^c\}$ in M.

In one extreme case, the functions $\tau(x)$ and $\beta(x)$ have a single *constant* value; then, however, their derivatives are zero, and the buffering parameters are not defined. In another special case, transfer and buffering functions are *linear functions* of the type $f_i\colon x \rightarrow a_i \times x$ (where a_i is a constant with $a_i \in \mathbb{D}$ and $a_i \neq 0$); in that case, the buffering parameters are constant over the entire domain. This case corresponds to the standard situation in probability theory (if one chooses to interpret the measures t, b, T, B in terms of probabilities, with the independent variable x representing the number of events): Every possible "outcome" is associated with a probability that is assumed to be constant and independent from the number of events. For instance, the probability of the outcome "head" is supposed to remain the same, how ever often the coin is flipped.

This assumption of constant partitioning is usually inadequate when the measures t, b, T, and B are interpreted in terms of chemical buffering, with x representing the total quantity in question to be buffered. For instance, in a solution of a weak acid, the addition of H^+ ions is associated with progressively decreasing buffering strength.

The basic dichotomous structure is obvious in classic buffering phenomena.

Multi-partitioned system can be transformed in multiple ways into a formally correct buffered systems. The resulting two functions (transfer and buffering function) posit two complementary roles, namely of that which is being buffered, and of that which is buffering. Whether these roles are meaningful and useful depends on extrinsic criteria, not on formal ones.

In H^+ buffering, for instance, this basic dichotomic structure is evident as the dichotomy between bound vs. free H^+ ions. In physiological solutions, several H^+ buffer species are usually present simultaneously, each binding H^+ ions in its own way. In principle, these binding processes may be described individually by respective functions, each with total H^+ ions as the independent variable. Moreover, the amount of free H^+ ions (i.e., H^+ ions bound to water) is described by another function of total H^+ ions. Of the buffer species, two or more may happen to have identical binding characteristics and concentrations; then, the collection of functions contains duplicates and therefore constitutes a bag, not a set. Alternatively, we may wish to describe the buffering process in terms of individual buffer molecules, which exist in multiple copies with identical properties. These copies are described by identical functions, and thus the resulting collection of functions is again not a set.

Bags (or multisets) offer themselves naturally as a convenient data structure in order to formalize the physical or chemical processes in these ways for the computation of H^+ buffering strength. The functions are grouped into two bags of functions that describe H^+ binding to water and to other molecules, and are lumped together into "transfer function" and "buffering function", respectively. Buffering of Ca^{++} or other ions is treated analogously.

Buffering phenomena can present as non-conservative, multi-partitioned systems.

For H^+ buffering, the classic case of buffering, the dichotomy of "bound vs. free" provided an intuitive criterion for grouping the elements of the whole into two meaningful, complementary groups. For other buffering phenomena, such as redox buffering or blood pressure buffering, analogous criteria are less obvious. Moreover, buffering phenomena do not necessarily correspond to the partitioning of a conserved quantity into two partitions, i.e., to "conservative" two-partitioned systems. Rather, many scientifically relevant buffering phenomena present in some other, non-conservative form.

Four basic categories of partitioned systems can be distinguished: *i)* Conservative, two-partitioned systems, denoted ${}^2\Pi_c$. An example are H^+ ions in an aqueous solution of a single weak acid; *ii)* Conservative multi- or n-partitioned systems; an example are H^+ ions in a solution of several buffer species of different concentration and with different K_d values. For these systems, we use the shorthand ${}^n\Pi_c$; *iii)* General two-partitioned systems, including both conservative and non-conservative ones; those systems are denoted by ${}^2\Pi$; *iii)* Multi-partitioned systems in the general sense, including both conservative and non-conservative ones; they are denoted by ${}^n\Pi$. The terms "multi-partitioned system" and "collection of functions of a commmon independent variable" are synonymous.

Equivalences between conservative and non-conservative, or between multi-partitioned and two-partitioned systems

Importantly, there are certain transformations or interconversions between these four classes of partitioned systems that are invariant with respect to the buffering parameters t, b, T, and B. Multi-partitioned systems (${}^n\Pi$ or ${}^n\Pi_c$) can be transformed into two-partitioned systems (${}^2\Pi$ or ${}^2\Pi c$) and *vice versa*; two-partitioned systems (${}^2\Pi$) can be transformed into conservative (${}^2\Pi_c$) or even into standardized two-partitioned systems and *vice versa (see below)*. For any *non-conservative* two-partitioned system there exists at least one *conservative* two-partitioned system system that is equivalent in terms of the four buffering parameters. Conservative systems, on the one hand, are simple

and provide a kind of standard which makes it easy to compare different systems. Non-conservative systems, on the other hand, may offer important advantages of their own. It is of practical interest to know how the systems of these various classes are related and can be interconverted.

From multi-partitioned to two-partitioned systems: $^n\Pi \rightarrow {}^2\Pi$ transformations

Buffering and the four buffering parameters t, b, T, and B are defined for systems of exactly two functions, but not for systems of more functions. We can transform, however, any n-partitioned system, conservative or not, into a two-partitioned system. Reduction of n functions to 2 functions is achieved by concatenating ("fusing" or "merging") the many partitions until there are only two, not necessarily nonempty, partitions left. Their ordered combination yields a buffered system.

Obviously, concatenation and ordering can be done in several, different ways, corresponding to different views of what is considered "that which is buffering" as opposed "that which is being buffered". In general, the maximum number of two-partitioned systems that can be formed from one n-partitioned system is 2^n; this number is smaller than 2^n if not all n elements of the system are different, i.e., if the bag F is not a set.

Such $^n\Pi \rightarrow {}^2\Pi$ transformations are possible and necessary before buffering terminology can be applied in a meaningful way to a multi-partitioned system. It is important to keep in mind that this transformation is not unique: several equally valid two-partitioned systems can be derived from one n-partitioned system, and these will have different buffering properties. The axiomatic definition of the buffering parameters is compatible with any of these two-partitioned systems, and preference of a particular one must therefore be justified on other grounds. For instance, it is usually meaningful to lump together all the chemically heterogeneous buffer species in physiological solutions into a single virtual buffer as opposed to the solvent water in order to express overall H^+ or Ca^{++} buffering strength.

Buffering in non-conservative two-partitioned systems

Buffered systems derived from conservative two-partitioned systems satisfied the "conservation condition" $\sigma'(x)=1$; they thus corresponded to plane space curves in R^3 that lie inside the standard plane x-y-z=0, or in planes that are parallel to the standard plane *(Figure 1D)*. In principle, however, our measures of buffering can be computed for *any* ordered pair of differentiatable functions, conservative or not. For instance, the buffering ratio B is given by ratio of two first derivatives $\tau'(x)/\beta'(x)$, and that ratio clearly exists for any combination of differentiable functions. Similarly, we can always compute the parameters t, b, and T.

The existence of these parameters suggests that we can talk about "buffering" in non-conservative systems in the same meaningful and exact way as in conservative systems. In terms of space curves, buffering parameters are defined, meaningful and measurable in systems that lie within the standard plane, in parallel planes, in non-parallel planes, or in fact even in certain non-planar surfaces. In a particular scientific context, such non-conservative systems of functions can represent the "partitioning of a non-conserved quantity". This novel and therefore somewhat unfamiliar concept will become very useful for the treatment of "dimensionally heterogeneous" systems such as blood pressure buffering *(Buffering II)*.

Linear or non-linear "distortion" engender non-conservative systems.

The simplest way to obtain a space curve that falls outside the standard plane is to add a non-zero constant c as an "offset" to the sigma function, such that $\sigma(x)=x+c$ and $\sigma(0)=c$. By definition, a system is non-conservative if $\sigma'(x)\neq 1$. In our case, we find $\sigma'(x)=1$, and therefore the system again conservative in the strict sense.

Simple non-conservative systems are obtained by scaling up or down all y and z values of a standardized partitioned system by a constant factor. We speak of a "linearly distorted" system if the corresponding sigma function has the form $\sigma(x)=d\times x$, where d is a constant real number different from zero and from 1. The corresponding

Figure 1: Equivalences between different partitioned systems.

A, x-equivalents (column) and yz-equivalents (row), including their respective standard forms

Various two-partitioned systems of functions are represented as area plots showing the values of two partitioning functions π_1 and π_2 *(ordinate)* of a common independent variable *(abscissa)*. The partitioning functions of a given system *(center panel)* can be transformed in multiple ways such that the proportion between their individual rates of change (i.e., buffering) is preserved for any value of x *(top and bottom panels)*, or alternatively for any y or z or pair (y,z) *(left and right panels)*. One particular x-equivalent, termed the "canonical" or "standard" partitioned system, has a sigma function $\sigma(x)=x$. Analogously, there are canonical y- and the z-equivalents for which $\sigma(x)=x$. Among the systems that are equivalent in both y *and* z, the canonical one satisfies the slightly more general condition $\sigma(x)=x+c$, where c is a constant.

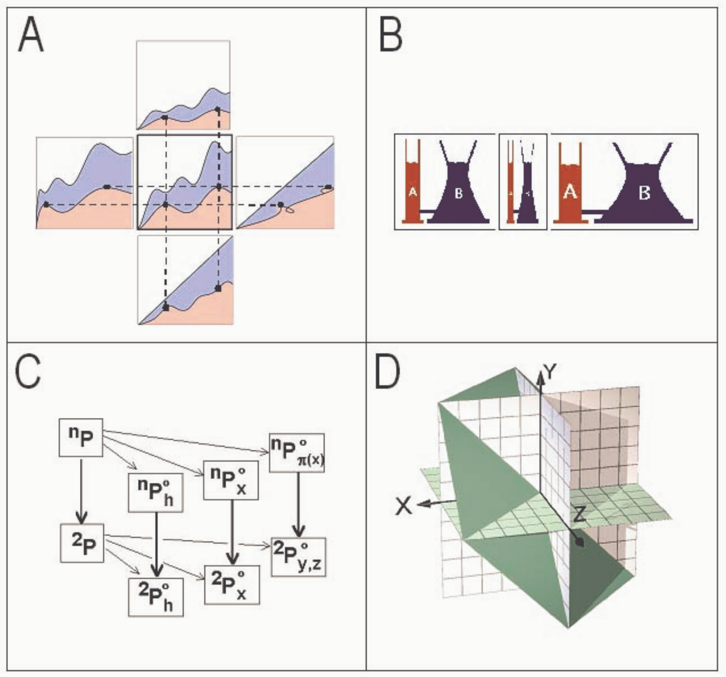

C, Interconversions and standardization of partitioned systems

Multi-partitioned systems *(upper row)* need to be converted *(vertical arrows)* into two-partitioned systems in order to form buffered systems *(lower row)* in which buffering can be quantitated. A given n- or 2-partitioned system can be transformed into a "canonical" or "standardized" system that is equivalent with respect to the proportion between the rates of change of the individual partitioning functions at all values of x or y or z or some other parameter h. The order in which the two transformations are carried out that lead from a n-partitioned system to a standardized two-partitioned system does not affect the result, i.e., these transformations are commutative.

B, Parametric forms, or h-equivalents

Partitioned systems may be compared directly at similar x values, but also for points that correspond with respect to any arbitrary parameter h. In this figure, two-partitioned systems are visualized as systems of communicating vessels. Total fluid volume is represented by the independent variable x, and individual fluid volumes in A and B by the values of the partitioning functions π_1 and π_2. Rates of change become equal to volume flows. When there is a net flow into the entire system, the proportion between flow into A vs. B at similar total volumes *(x)* or similar individual volumes *(y* or *z)* is different between the three systems. However, when expressed in parametric form as functions of fluid level h, the four buffering parameters t(h), b(h), T(h) and B(h) are identical in the three systems for a given value of h.

D, Standard plane

Within each equivalence class formed by those partitioned systems that exhibit similar buffering behavior at same values of x (or y or z or h), there is exactly one partitioned system corresponding to a space curve in R^3 that crosses the origin and falls into the plane described by the equation $x-y-z=0$. This curve is called the "canonical" or "standard" representative of the entire class, and the plane is termed "standard plane".

space curves lie within a plane given by the equation $[d\times x - y - z] = 0$. In more involved distorted systems, the factor d may not be constant, but vary as a function $d(x)$ of x. Then, the sigma function becomes a non-linear function of x, and we speak of a "non-linearly distorted" system. Space curves corresponding to non-linearly distorted systems lie within a non-planar surface given by the equation $[\sigma(x) - y - z] = 0$; this equation describes a surface that is symmetrical with respect to the plane that goes through the origin of the axes and is perpendicular to the standard plane. In principle, the sigma function may be any differentiable function, including periodic and chaotic ones.

Thus, a two-partitioned system has a plane space curve as geometrical equivalent if it is conservative. On the other hand, if $\sigma(x)$ is a sine wave, for instance, the two-partitioned system has as geometrical equivalent a space curve within a surface that resembles a tilted tin roof. Parametrization can thus turn conservative systems into complicated non-conservative systems without altering buffering behavior. Reversly, partitioned systems that present initially in a rather involved form can be transformed into the much simpler conservative systems.

"Dimensional heterogeneity" engenders non-conservative systems.

Another factor comes into play as one moves from pure numbers to „real world phenomena" where it is often necessary to employ scientific units of a particular physical dimension. The case is simple if x, $\tau(x)$, and $\beta(x)$ are all of identical physical dimension. Such systems are termed here „dimensionally homogeneous" systems. In other cases, however, the independent variable x is of one dimension, while transfer and buffering functions are of another. Then, we say the system is „dimensionally heterogeneous" (yet other systems that are of different dimensions in transfer vs. buffering function rarely make sense and are not considered here).

In dimensionally heterogeneous systems, transfer, buffering, and sigma functions implicitly include a conversion factor K that brings about the difference between their physical dimension and particular unit on the one hand, and dimension and unit of the independent variable x on the other. In simple cases, this factor is a constant K: $\sigma(x) = x\times K$. Then, K can be written as the product of a dimensionless scaling factor d and a unit {D} of a certain physical dimension: $K=d\times\{D\}$. For instance, expressing distance l as a function $l{:}t\rightarrow l(t)$ of time requires a conversion factor K with the dimensions of a velocity={length/time}. Various combinations of scaling factor d and unit {D} may yield a given conversion factor K; for instance, K=1 meter/second ↔ 1.093×{yards/second} ↔ 39.37×{inches/second}.

Importantly, the four buffering parameters t, b, T, and B remain dimensionless even in dimensionally heterogeneous systems. This property follows from the way in which these parameters were defined, and turns the parameters t, b, T and B into extremely versatile scientific units. For instance, the changes of total, bound and free H^+ ions that constitute classical acid-base buffering can be expressed either exclusively in terms of molar concentrations, or one may combine molar units (for added H^+ ions) with grams, equivalent charges, or absolute numbers (for free and bound H^+ ions). The four measures will invariably assume the same values, irrespective of the particular representation chosen. On the other hand, the possibility to handle dimensionally heterogeneous systems provides the key to a quantitative treatment of several important buffering phenomena, such as blood pressure buffering and systems level buffering *(Buffering II)*.

Non-conservative systems that are both distorted and dimensionally heterogeneous

In the simplest case of a dimensionally heterogeneous system, the conversion factor K is equal to a physical unit 1×{D}, with the scaling factor d being equal to 1. The sigma function is then $\sigma(x)=K\times x=\{D\}\times x$, and its derivative $\sigma'(x)=K=\{D\}$. If {D} is a genuine physical unit (and not, trivially, a dimensionless number), then the first derivatives $\tau'(x)$, $\beta'(x)$, or $\sigma'(x)$ can never equal unity. Therefore, dimensionally heterogeneous systems are always

"non-conservative". The converse statement is not true, however: not all non-conservative systems are dimensionally heterogeneous.

As with dimensionally homogeneous sytems, we can carry the generalization of heterogeneous systems even further and drop the constraint that the scaling factor d should be a constant. Thus, such a system only needs to satisfy the condition $\sigma'(x) = d(x) \times \{D\} \times x$ where $d(x)$ may stand for any dimensionless, continuous, differentiatable function. Such systems may be termed „dimensionally heterogeneous, nonlinearly distorted systems". Again, blood pressure buffering and systems level buffering are examples for such systems *(Buffering II)*. Dimensionally homogeneous and heterogeneous systems have similar geometrical equivalents.

Interconversions between conservative and non-conservative systems

Conservative and non-conservative systems may exhibit identical buffering behavior.

The parameters t, b, T or B put a constraint only on the proportion between $\tau'(x)$ and $\beta'(x)$, i.e., the rates of change of transfer and buffering function, but not on the absolute rates. Thus, scaling these rates of change jointly up or down will change $\sigma'(x)$ proportionally to a value different from 1, but will not change the buffering parameters. Reversly, two functions of a non-conservative system (i.e., with $\sigma'(x) \neq 1$) can always be rescaled to yield a corresponding conservative system with identical buffering parameters. Put geometrically, for any arbitrary space curve there is a corresponding curve within the standard plane thus that the proportion between the two slopes dy/dx and dz/dx is identical at any two corresponding points of the two curves.

Conservative two-partitioned systems provide the simplest possible representation of buffered systems.

We can thus think of a given conservative two-partitioned system as the "original" form of a two-partitioned system with particular buffering properties, and of the equivalent non-conservative partitioned systems as the results of a "deformation" of that system. Among these equivalent systems, conservative two-partitioned systems are distinguished by the fact that the corresponding space curves are confined to a plane in R^3. In fact, they are purely two-dimensional entities inasmuch they can be represented by a linear combination of only two independent unit vectors, e.g. $\lambda_1=(1,1,0)$ and $\lambda_2=(1,0,1)$. The plane defined by these two vectors was termed "standard plane". This mapping from non-conservative to conservative system is a "projection" because it maps a three-dimensional system into two dimensions (i.e., a map $\mathbb{R}^3 \rightarrow \mathbb{R}^2$). With respect to the number of dimensions, the two-dimensional representation provides the most simple way to formulate a two-partitioned systems of certain buffering properties. On the other hand, there exists an infinite number of such projections. In order to be useful, this collection of systems needs to be structured further.

Partitioned systems can be compared under different aspects.

Consider the concept of a "vector": Among all possible "arrows" in a particular space, all those are said to be identical whose length and direction are identical. This definition is an abstraction that ignores some aspects (position), and compares different elements solely under selected aspects (length and direction). Elements that are similar with respect to these selected aspects form an "equivalence class" that is induced by an "equivalence relation". For vectors, this equivalence relation is "*A has same length and direction as B*". Note that the equivalence classes induced by a particular equivalence relation in a set of elements form a partitioned system.

Similarly, the comparison of partitioned systems of functions under a particular aspect is a fundamental and useful scientific operation. For instance, one usually compares H^+ buffering strength in different samples at similar values of pH_i or $[H^+]_i$, corresponding to similar values of the transfer function $\tau(x)$, as elaborated in detail in the accompanying paper *(Buffering II)*. In other situations, it is more appropriate to compare different systems at similar values of the independent variable x. For instance, it makes sense to compare the buffering of organ perfusion in the

face of variable perfusion pressure in different organs (e.g. in kidney and brain) at similar perfusion pressures (i.e., similar x values) rather than at similar blood flows (i.e., similar values of the transfer function $\tau(x)$) *(Buffering II)*. In yet other situations, it may be most natural or useful to compare systems under an aspect that is not reflected by any one of the parameters x, $\tau(x)$ or $\beta(x)$, but by some further parameter. For instance, we may want to compare the buffering behavior of different systems of communicating vessels at similar fluid levels h which is related only indirectly to x.

Comparing partitioned systems under a particular aspect induces equivalence classes.

Obviously, systems may be similar under one aspect, and different under another one. The question whether buffering is similar for "corresponding points" of different systems can thus only be answered after specifiying a criterion that specifies unambiguously which are the corresponding points. The systems that exhibit similar buffering at "corresponding points" form an equivalence class. For instance, the criterion "*same buffering behavior at same x*" (e.g. perfusion pressure) induces equivalence classes which we term "x-equivalents" *(Figure 1A, panels in same column)*, the criterion "*same buffering at same y*" (e.g. pH) induces equivalence classes which we term "y-equivalents" (*Figure 1A, panels in same row)*, and the criterion "*similar buffering behavior at similar values of parameter h*" (e.g. fluid level) induces equivalence classes which can be termed "h-equivalents" *(Figure 1B)*. Analogously, other parameters used to express a system in parameteric form will induce other equivalence classes.

An entire equivalence class can be represented by a unique "canonical" element.

Vectors that are similar have similar direction and length, but may be located at different positions in space. Among all similar vectors, the one that starts at the origin of the axes can be represented in a particularly clear and simple form, both algebraically and graphically. This "canonical" form allows one to study the relevant properties of all members of the equivalence class in a convenient way. In topology, the reduction of an equivalence class into a single representative element is termed "identification".

Similarly, we can identify classes of equivalent partitioned systems with a single "canonical" or "standard" system. We define as canonical that element $\Pi°$ of a class of equivalent partitioned systems Π whose sigma function has a unity slope and whose individual partitioning functions have zero value at $x=0$:

$$\Pi° = \{\Pi \mid \sigma'(x)=1 \wedge \pi_i(0)=0\}$$

Put in geometrical terms and for a two-partitioned system, of all space curves in an equivalence class, the standard or canonical one is that unique space curve that falls into the standard plane and crosses the origin of the three axes.

The second condition is not applicable, of course, to those partitioning functions whose absolute values are used to define the class. This is the case, for instance, with y- and z-equivalents; the canonical system then is the one that satisfies the weaker condition $\sigma(0)=0$. Furthermore, the *y-and-z-*equivalents of a two-partitioned system are fully constrained in both the y- and z-values, such that $\sigma(0)$ may have any value. The same constraints guarantee, however, that there is only a single conservative system that can be used as standard equivalent in this case.

The conversion of non-conservative systems into standard conservative systems is termed a $\Pi \rightarrow \Pi°$ transformation. Note that standardization of dimensionally heterogeneous systems will turn them into dimensionally homogeneous systems.

Example: finding the canonical x-equivalent, or "standardization in x"

To illustrate the basic principles, consider a simple ${}^2\Pi \rightarrow {}^2\Pi°$ transformation, namely the transformation of a conservative two-partitioned system ${}^2\Pi$ with offset into a conservative two-partitioned system ${}^2\Pi°$ without offset. Geometrically, this means translating a plane space curve into the standard plane from some other, parallel plane. In order to obtain an x-equivalent,

this translation furthermore must not change the four buffering parameters at any value of the independent variable x, such that only the dependent variables y and z may be changed.

Analytically, this type of standardization is achieved by replacing each partitioning function $\pi_i(x)$ by a corresponding standard partitioning function $\pi°_i(x)$ that satisfies the equation $\pi°_i(x) = \pi_i(x) - \pi_i(0)$. In geometrical terms, this standardization simply removes an offset by translating the entire curve parallel to the yz-plane towards the standard plane such that the curve crosses the origin. Such a translation is defined by a vector $-\vec{v}°$ with

$$\vec{v}° = \begin{pmatrix} y_0 \\ z_0 \end{pmatrix} = \begin{pmatrix} \pi_1(0) \\ \pi_2(0) \end{pmatrix}.$$

The resulting unique two-partitioned system $\Pi^o_x = \{\pi°_1(x), \pi°_2(x)\}$ is said to be the "canonical" or "standard x-equivalent" of ${}^2\Pi$, of which there is exactly one. Carrying out this transformation in the reverse direction (i.e., by using a vector $\vec{v}°$ of similar length and opposite direction) transforms the standard system Π^o_x into a "non-standard x-equivalent" of Π^o_x. For each standard partitioned system there is, however, an infinite number of ${}^2\Pi$ systems that are, with respect to the buffering parameters at a given x, equivalent to Π^o_x as well as to each other.

Example: finding the canonical y,z-equivalent, or "standardization in the dependent variables"

Alternatively, one can carry out the standardization such that the buffering parameters are preserved for any given pair (y,z). Analytically, this is achieved by substituting a given non-standardized partitioning function $\pi_i(x)$ with a standard partitioning function $\pi°_i(x)$ for which $\pi_i(x) = \pi°_i[x+\pi_1(0)+ \pi_2(0)] = \pi°_i[\sigma(x)]$ (to be read as a function $\pi°_i$ of the argument $[\sigma(x)]$ – a nested function, not a product). Geometrically, this corresponds to a classical parallel projection of the original space curve onto the standard plane along lines parallel to the x axis. The resulting unique two-partitioned system $\Pi^o_{y,z}$ $\{\pi°_1(x), \pi°_2(x)\}$ is said to be the "standard y,z-equivalent". Again, carrying out such transformations in the reverse direction results in a family of non-standardized ${}^2\Pi$ systems that are "non-standard equivalents in x" of the standard system as well as of each other.

Standardization in the dependent variables: non-linearly distorted systems & general form

Analytically, the $\Pi^o_{y,z}$ transformation of a system of two arbitrary functions $y=\pi_1(x)$ and $z=\pi_2(x)$ is achieved by substituting for π_1 a partitioning function $\pi°_1$ that satisfies the condition

$$\pi_1(x) = \pi°_1[\pi_1(x)+ \pi_2(x)] = \pi°_1[\sigma(x)],$$

and for π_2 a partitioning function $\pi°_2$ for which

$$\pi_2(x) = \pi°_2[\pi_1(x)+ \pi_2(x)] = \pi°_2[\sigma(x)].$$

Standardization in the dependent variables for general multi-partitioned systems is achieved by substituting each function π_i with a standard partitioning function $\pi°_i$ that satisfies the condition

$$\pi_1(x) = \pi°_1[\pi_1(x)+ \pi_2(x)\ldots+ \pi_n(x)] = \pi°_1[\sigma(x)].$$

Geometrically, only standardizations of two-partitioned systems (i.e., $\Pi^o_{y,z}$ transformations) have equivalents in R^3. Here, this standardization corresponds to a classical parallel projection of the space curve onto the normal plane parallel to the x axis. In vector notation, this projection is given as

$$\begin{pmatrix} x \\ \pi_1(x) \\ \pi_2(x) \end{pmatrix} \rightarrow \begin{pmatrix} \sigma(x) \\ \pi_1(x) \\ \pi_2(x) \end{pmatrix}.$$

When $n>2$, the ${}^n\Pi \rightarrow {}^n\Pi°$ standardizations in the dependent variables lack a geometrical equivalent in R^3.

Standardization in x: Linearily distorted systems without and with offset

Next, consider a two-partitioned system for which $\sigma(x)=a\times x$. Except for the trivial case of $a=1$, such a system is non-conservative because $\sigma'(x)\neq 0$, and can thus be classified as a "linearily distorted partitioned system without offset". Standardization of such systems in x is achieved by replacing each

partitioning function π_i by a corresponding standardized partitioning function π_i° for which

$$\pi_i^\circ(x) = \frac{\pi_i(x)}{a}.$$

Geometrically, this standardization is equivalent to projecting each point of the original curve onto the standard plane along it's shortest connection with the x-axis.

For a linearily distorted partitioned systems with offset (i.e., with $\sigma(x)=a\times x+c$ and $c\neq 0$), the "standard x-equivalent" is obtained by substituting for any given function π_i a standard partitioning function π_i° for which

$$\pi_i^\circ(x) = \frac{\pi_i(x)-\pi_i(0)}{a}.$$

Geometrically, this corresponds to the composition of a translation (forcing the entire curve through the origin) and projection of each point onto the normal plane along it's shortest connection with the x-axis.

Standardization in x: non-linearly distorted systems & general form

Next, we need to consider the standardization in x of a general, non-conservative two-partitioned systems with an arbitrary nonlinear sigma function $\sigma(x)$; in brief, these are called $^2\Pi\to\Pi_x^\circ$ transformations.

Analytically: In general, the standardization in x of a two-partitioned system can be achieved by transforming π_I into a standardized partitioning function π°_I such that

$$\pi^\circ_1(x) = \int_0^x \left(\frac{\pi'_1(x)}{\pi'_1(x)+\pi'_2(x)} \right) dx = \int_0^x \left(\frac{\pi'_1(x)}{\sigma'(x)} \right) dx$$

and π_2 into a function $\pi^\circ_2(x)$ such that

$$\pi^\circ_2(x) = \int_0^x \left(\frac{\pi'_2(x)}{\pi'_1(x)+\pi'_2(x)} \right) dx = \int_0^x \left(\frac{\pi'_2(x)}{\sigma'(x)} \right) dx \quad .$$

Multipartitioned, or $^n\Pi$ systems, with more than two functions can be standardized analogously. Thus, $^n\Pi\to{}^n\Pi^\circ$ transformations in x are carried out by substituting each function $\pi_i(x)$ with a standard partitioning function

$$\pi^\circ_i(x) = \int_0^x \left(\frac{\pi'_i(x)}{\pi'_1(x)+\pi'_2(x)\ldots+\pi'_n(x)} \right) dx = \int_0^x \left(\frac{\pi'_i(x)}{\sigma'(x)} \right) dx \quad .$$

Geometrically: $\Pi\to\Pi_x^\circ$ transformations do not correspond to any of the classical projections. For two-partitioned systems represented geometrically as space curves in R^3, they can be visualized in two ways.

Firstly, a $^2\Pi\to{}^2\Pi_x^\circ$ transformation can be achieved by dividing the non-standardized space curve into n linearized segments of length Δx, rescaling them individually in y and z directions by a common factor such that $(\Delta y+\Delta z)=\Delta x$ while keeping the ratio $\Delta y/\Delta z$ constant, joining the rescaled elements tip to end, and the forcing the resulting space curve through the origin by translating it along a vector that is perpendicular to the x-axis. This procedure is carried out with infinitely small and infinitely many segments ($n\to\infty$ and $\Delta x\to 0$).

Secondly, $^2\Pi\to{}^2\Pi_x^\circ$ transformations may be thought of as an iterative projection, illustrated here for a buffered system for which $x\in\mathbb{R}^+$: *i)* Division of the curve into n individual points $P_i=P(x_I)$, $P(x_2)$ … with $x_i= x_0,x_I,\ x_2$ …and $x_i<x_{i+1}$ and $n\to\infty$; *ii)* Translation of the entire space curve along a vector

$$\vec{v}^\circ = \begin{pmatrix} y_0 \\ z_0 \end{pmatrix} = \begin{pmatrix} \pi_1(0) \\ \pi_2(0) \end{pmatrix};$$

this forces the curve through the origin, and $P(x_0)$ into the standard plane; *iii)* Translation of all points $\{P(x_i)|\ x_i>x_0\}$ along a line that is parallel to the yz-plane and has a slope $d\pi_2(x_I)/d\pi_I(x_I)$ until the point $P(x_I)$ falls into the standard plane; *iv)* Projection of all points $\{P(x_i)|\ x_i>x_I\}$ (these points have been translated twice by now) along along a line that is parallel to the yz-plane and has a slope $d\pi_2(x_2)/d\pi_I(x_2)$ until the $P(x_2)$ falls into the standard plane; *v)* This procedure is continued to the end of the curve. The process thus successively unrolls and compresses (or stretches) the space curve onto the standard plane.

Some rules for transformations

Standardization in x always yields single-valued partitioning functions, but standardization in the dependent variables not necessarily so, even

when the individual partitioning functions $\pi_i(x)$ are all single-valued. Moreover, standardization in y,z does not force the space curve through the origin.

$^n\Pi \rightarrow {}^2\Pi$ and $\Pi \rightarrow \Pi^\circ$ transformations of systems of functions can be carried out sequentially, either in the order $^n\Pi \rightarrow {}^n\Pi^\circ \rightarrow {}^2\Pi^\circ$ or $^n\Pi \rightarrow {}^2\Pi \rightarrow {}^2\Pi^\circ$. In either case, the overall result is a $^n\Pi \rightarrow {}^2\Pi^\circ$ transformation. Provided that the two transformations are either both in x, or both in y and z, the result is independent from the path taken. In other words, these transformations are commutative.
