# Supplementary material for: The quantitation of buffering action I. A formal & general approach
Source: Theor Biol Med Model. 2005 Mar 15;2:8. doi: 10.1186/1742-4682-2-8 (PMC1079953; doi:10.1186/1742-4682-2-8)
Supplement: Additional File 9 — Some Useful General Principles Regarding the Practical Application of the Formal & General Buffering Concept [file 1742-4682-2-8-S9.pdf]

Theoretical Biology and Medical Modelling

Research

The quantitation of buffering action. I. *A formal and general approach.*

Bernhard M. Schmitt

Supplement 9:

Some Useful General Principles Regarding the Practical Application of the Formal and General Buffering Concept

From n-partitioned systems to buffered systems

From multi-partitioned to two-partitioned systems: a necessary but indetermined step.

The above axioms defined the "signed probability measure t" based on a bag of functions, and implied the grouping of the bag's elements into two complementary subbags. In the vessel model, the equivalent to this simple bag algebraic operation is to replace several individual vessels by a single vessel of the same aggregate cross-sectional areas *(Figures 2 C and D in the main text of Buffering I)*. Obviously, both "transfer functions" and „buffering function" may be the sum of several, possibly identical partitioning functions. This regrouping, termed ${}^{n}\Pi \rightarrow {}^{2}\Pi$ transformation, can be done in various ways. For instance, we might group together the fluid volumes inside vessels B and D on the one hand, and in vessels A, C, and E on the other, resulting in a system B5 given as B5 = { $\tau(x)=0.4\times x$, $\beta(x)=0.6\times x$ }.

From two-partitioned to buffered systems: also a necessary, indetermined step.

Above, we further defined „buffered systems" as an ordered pair of functions. The formal aspect that the pair of functions requires an order structure in order to speak meaningfully about buffering can again be illustrated by the two communicating vessels shown in *Figure 2D* of the main text of *Buffering I*. Instead of taking vessel A as the „buffering vessel", we might as well ask how strong the fluid volume inside vessel B is buffered against fluid entering or leaving the entire system. To answer that question, we assign the role of „transfer vessel" now to vessel B, resulting in a buffered system B4 = { $\tau(x)=x\times 0.8$, $\beta(x)=x\times 0.2$ }. Clearly, B3 and B4 are two different, yet formally equally correct buffered systems.

The simple yet important conclusion is, in other words: Who is the buffer, and who is being buffered never follows automatically from the mathematical representation of a given phenomenon, i.e., an unordered system of functions. Furthermore, a buffer is never constituted by some intrinsic material property. Rather, a buffer is constituted by its assigned position in an ordered pair of functions. How this assignment is done depends on the particular interests and circumstances. Among the various formally correct solutions, not all are equally use- and meaningful scientifically.

In acid-base chemistry, for instance, it is a reasonable convention to regard the binding H^+ ions to solvent molecules (termed "free H^+" graphically, though not chemically precise) as the parameter that is being buffered ("transfer function"), and the binding of H^+ ions to other molecules as buffering ("buffering function"). In other cases, however, this dichotomy of bound vs. free is not applicable, or induces no meaningful two-partitioned system.

Consider, for instance, electrons equilibrating via redox reactions between various chemical compounds *(Buffering II – Supplement 3)*. A scientist interested in a particular redox-regulated enzyme is likely to treat anything as a "buffer" that stabilizes this enzyme's oxidization state in the face of added redox equivalents, including for instance Fe^{++} or Fe^{+++}, but excluding the enzyme itself. Another scientist, however, may be interested in iron metabolism, and the cytoplasmic levels of Fe^{++} in particular. To him, anything is a "buffer" that stabilizes the concentration and oxidization state of Fe^{++}, and these buffers will include redox-regulated enzymes, but exclude Fe^{++} itself. In either case, it would not make much sense to treat the particular species of interest as it's own buffer, as some units imply [1].

From non-conservative systems to standardized systems – $^n\Pi \rightarrow {}^n\Pi°$ transformations.

Non-standardized systems may offer advantages over standardized ones

So far, we have described the volume V_i in an individual vessel as a function $V_i(x)$ of the total volume x inside the system of communicating vessels. Such an approach results in a conservative partitioned system. Conservative systems are conceptionally simple, but have the disadvantage that the partitioning functions depend on the entire system, not only on the individual vessel itself. Consequently, when vessels are added or removed, the function will change, too. Modelling and rearranging systems of communicating vessels (and any other systems) would be easier if we had a single invariant, system-independent function to describe a given vessel. These requirements can be met by describing volume V_i as a function of the fluid level h, i.e., $V_i = \tau^*_i(h)$. Such functions are known as "parametric functions". In this example, the parametric equivalent $\tau^*_i(h)$ of the partitioning function -itself a partitioning function-, provides a kind of generic building block for larger systems. They are practically important and common. For instance, the binding characteristics of ligands to various "buffers", e.g. those of Ca^{++} ions to various chelating agents, are usually expressed as functions of free ligand concentration (corresponding to a parametric function), and not as functions of total ligand concentration (corresponding to a standardized partitioning function).

Standardization with fixed dependent variables – comparing systems at similar fluid levels

Let us consider next the reverse transformation. Combining several such building blocks, i.e., vessels V_i with their individual functions $\tau^*_i(h)$, into a system of communicating vessels, results in a system of functions $^nP\{\tau^*1(h), \tau^*2(h), \ldots, \tau^*n(h)\}$ that is not a conservative partitioned system: the independent variable is given in terms of "fluid level h", and the two dependent ones in terms of "fluid volumes". In this form, the relation between total fluid volume inside the system and buffering is not immediately obvious. An explicit expression of buffering as a function of total volume x is obtained upon a $^n\Pi \rightarrow \Pi^\circ_{y,z}$ transformation or "standardization in the dependent variables". The yz-equivalent $\Pi^\circ_{y,z}$ of the "parametric" system $^n\Pi$ can be computed as outlined above.

In other cases, it may become necessary to compare the buffering properties of two systems at a given fluid level h, irrespective of the total fluid

volume inside the system. In that case, one would need to perform the reverse transformation, i.e., from a conservative two-partitioned system (expressed exclusively in terms of fluid volumes) into a non-conservative system (fluid volumes expressed as functions of fluid level).

Figure 1: Indicators, titrants, and system boundaries: Exploiting the "isohydric principle".

"Buffered systems" (enclosed by box) are abstractions that can be applied in various ways to the "real world", including physical systems (fluid filled communicating vessels); boundaries of physical system and of buffered system need not be identical.

A, Indicator.
In this system, the "transfer vessel" (A) is assumed to be inside a "black box" thus that the volume inside A cannot be determined directly. However, if an "indicator vessel" (I) that equilibrates in a known way with the "transfer vessel" is connected with it and accessible from the outside, one can determine the volume in A from the known volume in I and its known relationship to the volume in A. The indicator itself is not considered part of the system of interest.

B, Indicator and unknown buffer.
When a "buffering vessel" of unknown dimensions is connected to the transfer vessel, the buffering properties of the system inside the black box can be determined from the known total volume changes in the system and the observed volume changes in the indicator vessel, if the relation
between the volumes in indicator and transfer vessel is known.

C, Titrant.
Changes in the system of interest (inside box) can be brought about by connecting a "titrant vessel" (T) and letting it subsequently equilibrate. Physically, the titrant vessel is a part of the system of communicating vessels. It is not part of the "buffered system" of interest, however, and can be separated from it conceptually and computationally.

D, Titrant and indicator.
The known relationship between the volume in I and A together with the known volume changes in I and T allow a complete description of the buffered system inside the black box. The principles illustrated in the present Figure are generally valid, including the buffering of other quantities such as Ca^{++} ions, blood pressure, and systems level buffering *(Buffering II)*.

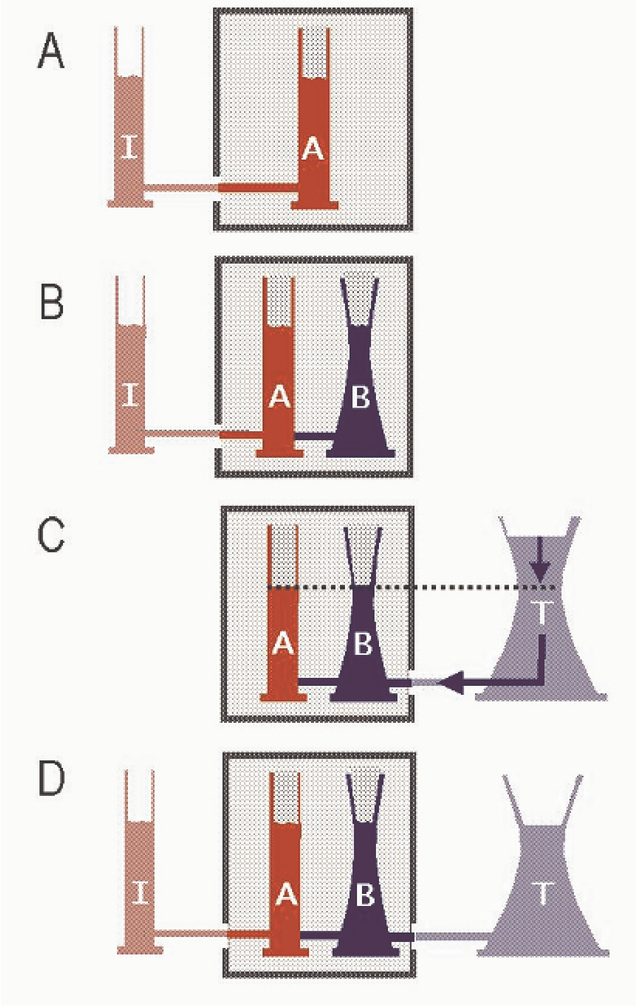

Standardization in the independent variable – comparing systems at similar volume content

Another type of standardization is required in order to compare the buffering properties of systems for a given value of the transfer or buffering function (in terms of the vessel model: for a given fluid volume inside the transfer or the buffering vessel). This comparison may be complicated if the systems employ different scales or units to express the dependent variables as a function of the same independent variable. Consider, for instance, two systems of communicating vessels that are geometrically equivalent, but calibrated in different units (e.g.liters vs. gallons). When titrating these systems using a beaker that is calibrated in liters, the systems will yield different numerical values of the respective transfer and buffering function, and the system becomes "linearly distorted without offset".

In order to compare buffering at a given filling state of the transfer or buffering vessel, it is necessary to express all variables in terms of liters. The transformation that achieves this was termed "standardization in the independent variable x". In this particular case of a linearly distorted system without offset, this transformation is achieved by replacing the original functions $\pi_i(x)$ of the system calibrated in gallons by standardized partitioning functions given as $\pi_i°(x) = \frac{\pi_i(x)}{K}$, with $K = d_{liters}/d_{gallons} \sim 0.264$. Partial buffering ratios B and partial buffering capacities BC are additive – a general form of the isohydric principle.

Reconsider *Figure 2* of the main text. The transfer vessel has a cross-sectional area $A_T = 1$, and the area A_B of the buffering vessel is therefore equal to the buffering ratio B. In *Figure 2D*, A_B corresponds to the sum of the individual cross-sectional areas of four such buffering vessels shown in *Figure 2C*. As a general rule, the buffering ratio B_{tot} of a system of communicating vessels simply equals the sum of all "partial" buffering ratios B_i that are observed when each buffering vessel individually is combined with that transfer vessel:

$$B_{tot} = \sum_{i=1}^{n} B_i .$$

An analogous relation exists for the buffering capacities: total buffering capacity BC_{tot} in a given interval $[y_1, y_2]$ is the sum of the "partial" buffering capacities:

$$BC_{tot}(y_1, y_2) = \sum_{i=1}^{n} BC_i(y_1, y_2) .$$

For systems of communicating vessels, the relation between the fluid volume V_T in the "transfer vessel" and the volume V_i in another, communicating vessel is not altered when other vessels are connected to or disconnected from this system.

The relations $B_{tot} = \sum_{i=1}^{n} B_i$ and $BC_{tot}(y_1, y_2) = \sum_{i=1}^{n} BC_i(y_1, y_2)$ are corollaries to the axioms that define the signed probability measure. They represent a generalized version of the "isohydric principle" well-known in acid-base chemistry. Stated in acid-base terminology, that principle says that the quantitative relationship between "free" H^+ ions and H^+ ions bound to a given chemical buffer is constant and not affected by additional chemical buffers that equilibrate with the same pool of free H^+ ions. In practice, this corollary makes it easy to compute the buffering ratios or capacities of buffer mixtures, a frequent situation in physiology and biochemistry. The additivity of buffering ratios and buffering capacities constitutes a fundamental property of buffered systems that will hold outside these particular fields.

Reference

1. R de Levie: **Redox Buffer Strength.** *J Chem Ed* 1999, **76:** 574-577.
