# Supplementary material for: The quantitation of buffering action I. A formal & general approach
Source: Theor Biol Med Model. 2005 Mar 15;2:8. doi: 10.1186/1742-4682-2-8 (PMC1079953; doi:10.1186/1742-4682-2-8)
Supplement: Additional File 10 — Historical Note: Origins of the Formal & General Approach [file 1742-4682-2-8-S10.pdf]

Theoretical Biology and Medical Modelling

Research

The quantitation of buffering action. I. *A formal and general approach.*

Bernhard M. Schmitt

Supplement 10:

Historical Note:
Origins of the Formal & General Approach

Henderson's „neutralizing power" - a dimensionless unit for buffering

The first quantitative measures of buffering strength was developed in acid-base physiology before the introduction of Sørensen's logarithmic unit for H^+ ion concentration. L.J. Henderson compared the buffering strength of weak acids with different dissociation constants by means of a measure termed „neutralizing power". He defined as neutralizing power of solution as the amount of strong base that is required to bring a unit volume from a particular acidic value to neutrality, divided by the difference between initial and final H^+ concentrations [1]. Slightly more general, we can rewrite this measure as the ratio of total H^+ ions added over a finite change in free H^+ ions. Neutralizing power is thus given as

$$\text{„neutralizing power"} = \left(\frac{\Delta[H^+]_{total}}{\Delta[H^+]_{free}}\right),$$

or, in the terminology of the present article, as

$$\text{„neutralizing power"} = \left(\frac{\Delta\sigma(x)}{\Delta\tau(x)}\right).$$

Henderson can thus be credited for suggesting the first dimensionless measure of buffering action.

Early versions of "buffering coefficient" and "buffering ratio" - Van Slyke against the man

Several years after Henderson, Van Slyke published his classical article *„On the Measurement of Buffer Values"* [2]. This was long after the advent of both Sørensen's logarithmic transform of free H^+ concentration into pH values in 1909 and of Koppel and Spiro's buffering strength unit in 1914 [3] which was based on Sørensen's novel scale. Van Slyke's article is well-known for the introduction of a buffering strength unit that subsequently became the widely adopted standard in acid-base chemistry or physiology. He defined this „buffering value β_{H^+}"

as a differential

β_{H^+} = d[Strong Base]/dpH

or, equivalently, as

$\beta_{H^+} = -d[H^+]_{total}/dpH$.

In the same article, albeit in smaller print *(pages 544-545)*, Van Slyke explored two further ways to quantitate buffering action, and these are of interest here because they do *not* employ Sørensen logarithmic transform of free H+ concentration. The first measure is essentially identical to Henderson's „neutralizing power", but expressed now as a genuine differential

$$\left(\frac{d[\text{Strong Acid}]}{d[H^+]}\right) \leftrightarrow \left(\frac{d[H^+]_{total}}{d[H^+]_{free}}\right).$$

In our formalism, this unit is equivalent to

$$\frac{d\sigma(x)}{d\tau(x)} = \frac{1}{t}.$$

As the inverse of the transfer coefficient, the expression 1/t is related to our system, but was not given an explicit role in it. Analogously, in probability theory, the inverse 1/p of Kolmogorov's probability p is not in use.

Van Slyke went on to suggest the following expression as the analytical solution for this measure of H+ buffering in a solution of a weak acid:

$$\left(\frac{d[H^+]_{total}}{d[H^+]_{free}}\right) \cong \frac{[A_{Tot}]\times K_A}{(K_A + [H^+])^2}$$

where $[A_{Tot}]$ is total concentration of weak acid, and K_A the acid constant. This equation is remarkable for two reasons. Firstly, as Van Slyke was fully aware, the description of H+ buffering by weak acids using this buffer value is greatly at odds with the description obtained using the unit β_{H^+}=d[Strong Base]/dpH. In particular, the differential $d[H^+]_{total}/d[H^+]_{free}$ is always greatest for $[H^+]_{total}=0$, whereas the buffering value β_{H^+} has a maximum which is located at $[H^+]_{free} = K_A$. Secondly, this expression is *not* the exact analytical solution for the differential $\left(\frac{d[H^+]_{total}}{d[H^+]_{free}}\right)$, contrary to Van Slyke's claim: Rather than using the exact analytical expression for $[H^+]_{total}$, Van Slyke had tacitly approximated $[H^+]_{total}$ by the amount of H+ bound to the buffer, given as

$$[H^+]_{bound} = \frac{[A_{Tot}]\times[H^+]}{K_A + [H^+]}.$$

Indeed, the difference between $[H^+]_{total}$ and $[H^+]_{bound}$ is negligible under most conditions encountered by the acid-base physiologist, where buffer-bound H+ ions outnumber free H+ ions by orders of magnitude. Importantly, however, what Van Slyke had thus actually presented under the name „buffering value" is the analytical solution for the differential

$$\frac{d[H^+]_{bound}}{d[H^+]_{free}} = \frac{dz}{dy},$$

and thus nothing else but our buffering ratio B under the wrong label. The differential dz/dy reappeared later in another ion-specific form, namely as Neher & Augustine's „calcium binding ratio κ_s"

$$\kappa_S = \frac{d[Ca^{++}]_{bound}}{d[Ca^{++}]_{free}}$$

(see below).

The second measure explored by Van Slyke was the expression

$$\left(1 - \frac{d[H^+]}{d[\text{Strong Acid}]}\right) \leftrightarrow \left(1 - \frac{d[H^+]_{free}}{d[H^+]_{total}}\right).$$

When expressed in our formalism as 1-dy/dx, it becomes obvious that, in such a conservative system where x=σ(x), this expression is identical with our buffering coefficient b:

$$\left(1 - \frac{d[H^+]_{free}}{d[H^+]_{total}}\right) = \left(1 - \frac{dy}{dx}\right) = (1-t) = b.$$

Van Slyke observed that this second measure yields values between 0 and 1 when applied to H+ buffering by weak acids, and that these values are

again at odds with the description afforded by the unit β_{H^+}.

Taken together, Van Slyke had come up with several possible ways to quantitate buffering action, one logarithmic and two non-logarithmic ones that correspond to our buffering coefficent b and buffering ratio B. He chose to advocate the logarithmic unit $\beta_{H^+}=d[H^+]_{total}/dpH$, and to discourage usage of the other two. Certainly consequential for acid-base physiology, this decision was by no means compelling on scientific grounds. Partly it rested on flawed arguments, partly on subjective preferences concerning mathematical notation.

With respect to the conflicts between the numerical values that are obtained with the various units, Van Slyke ruled in favor of the logarithmic buffering value β_{H^+}, saying that *„experience has led experimenters to decide that weak acids act most efficiently as buffers when they are present about half as free acid, [H⁺], half as salt, [Ba] …"* [2]. Here, Van Slyke was obviously misjudging the true nature of the conflict. „H^+ buffering" and „pH buffering" are different and incommensurate quantities, and as such, they cannot be in logical conflict with each other. Invoking experimental findings in this context merely leads to a circular argument, since these „findings" depend completely on the particular unit chosen. Interpretation of a given empirical data set according to the non-logarithmic units would undoubtedly show buffering values that are monotonously growing as H^+ concentration decreases; such things follow from algebra, not from titration.

The second criticism concerns Van Slyke's enthousiasm towards the logarithmic unit. This enthousiasm stemmed from a fascination by the perceived elegance and economy with which logarithmic notation handles numbers that vary over many orders of magnitude. A similar attitude among scientists might have promoted the subsequent wide acceptance of this buffering strength unit. A closer look at this argument suggests that it is no more convincing than the first one. Not only is notational elegance and economy paid for with an unability to express zero and negative numbers, but also is another, better solution provided by scientific notation (i.e., notation employing powers to the basis 10). For good reasons, and without sacrificing convenience or precision, scientific (!) notation is routinely used to express, for instance, distances from subatomic to astronomic ranges, or light intensities from single photons to entire sun systems.

The buffering coefficient β_c –Van Slyke reinvented.

Van Slyke was so successful in discouraging the use of his own non-logarithmic buffering strength units that he was not even credited when one of them, the dimensionless differential $d[H^+]_{total}/d[H^+]_{free}$, was reintroduced considerably later by Saleh and coworkers under the name *„buffering coefficient β_c"* [4]. The authors stayed behind Van Slyke insofar they failed to realize how profoundly this unit affects the description of buffering by weak acids or bases, and they did not provide analytical solutions. Importantly, however, the authors demonstrated the practical usefulness of this unit by detailing the experimental determination of β_c via NH_4 pulses and of the CO_2/HCO_3 dependent buffering component.

A main finding in this study was that cytoplasmic buffering power increased with increasing pH. The authors correctly pointed out that opposite conclusions are obtained in this case with the then „traditional" unit $d[H^+]_{total}/dpH$, and could clearly show that the conflict can be attributed entirely to distortions introduced by the logarithmic unit. On the other hand, the unit β_c does not yield a genuine ratio scale and is itself subjected to distortion under conditions of weak buffering.

The Ca^{++} binding ratio κ_s - Van Slyke's hidden jewel in a new mounting.

In a study on Ca^{++} gradients and Ca^{++} buffers in chromaffin cells, Neher and Augustine employed a measure for buffering strength [5] which was originally termed *"Ca^{++} binding capacity"* [5], now *"Ca^{++} binding ratio κ_s"* [6]. This measure was defined

as the ratio of change of bound Ca^{++} over change of free Ca^{++}:

$$\kappa_s = \left(\frac{d[Ca^{++}]_{bound}}{d[Ca^{++}]_{free}} \right).$$

Expressed in our notation, this turns into

$$\kappa_s = \frac{dz}{dy} = B .$$

Thus, the calcium binding ratio κ_s is the calcium-specific equivalent of the buffering ratio B introduced in this article. As detailed above, Van Slyke had introduced in 1922 the H^+ specific equivalent of the buffering ratio, namely the expression

$$\frac{[A_{Tot}] \times K_A}{(K_A + [H^+])^2} .$$

As specialized versions of the buffering ratio, both the Ca^{++}-specific and the H^+-specific measure yield dimensionless scales with equal intervals and an absolute zero for buffering.

The Ca^{++} binding ratio κ_s was adopted successfully by others in subsequent experimental studies. Less activity was recorded on a more theoretical level: Thus, the unit κ_s was never formulated in a general form that could have detached it from this particular ion species. Probably due to the lack of such an explicit formal generalization, the unit κ_s has remained confined to Ca^{++} research until today, failing to penetrate even into closely related areas such as Mg^{++} buffering.

Furthermore, the considerable impact of the unit κ_s on the description of buffering was neither pointed out explicitely nor worked out formally. For buffering of ions or quantities other than Ca^{++}, e.g. H^+ buffering in water or for redox buffering, this impact may have escaped attention due to the aforementioned lack of a general definition. Yet the description of Ca^{++} chelation itself using the unit κ_s differs considerably from the conventional ones, e.g. with respect to points of maximum buffering.

The generalized form of the unit κ_s was presented in this article. The impact of that unit on the quantitative description of various buffering phenomena is analyzed in the accompanying paper.

References

1. LJ Henderson: **Concerning the relationship between the strength of acids and their capacity to preserve neutrality.** *Am J Physiol* 1908, **21:** 173-179.
2. DD Van Slyke: **On the measurement of buffer values and on the relationship of buffer value to the disociation constant of the buffer and the concentration of the buffer solution.** *J Biol Chem* 1922, **52:** 525-570.
3. A Roos, WF Boron: **The buffer value of weak acids and bases: origin of the concept, and first mathematical derivation and application to physico-chemical systems. The work of M. Koppel and K. Spiro (1914).** *Respir Physiol* 1980, **40:** 1-32.
4. AM Saleh, G Rombola, DC Batlle: **Intracellular H+ buffering power and its dependency on intracellular pH.** *Kidney Int* 1991, **39:** 282-288.
5. E Neher, GJ Augustine: **Calcium gradients and buffers in bovine chromaffin cells.** *J Physiol* 1992, **450:** 273-301.
6. E Neher: **The use of fura-2 for estimating Ca buffers and Ca fluxes.** *Neuropharmacology* 1995, **34:** 1423-1442.
